# NEURD offers automated proofreading and feature extraction for connectomics

**DOI:** 10.1038/s41586-025-08660-5

**Published:** 2025-04-09

**Authors:** Brendan Celii, Stelios Papadopoulos, Zhuokun Ding, Paul G. Fahey, Eric Wang, Christos Papadopoulos, Alexander B. Kunin, Saumil Patel, J. Alexander Bae, Agnes L. Bodor, Derrick Brittain, JoAnn Buchanan, Daniel J. Bumbarger, Manuel A. Castro, Erick Cobos, Sven Dorkenwald, Leila Elabbady, Akhilesh Halageri, Zhen Jia, Chris Jordan, Dan Kapner, Nico Kemnitz, Sam Kinn, Kisuk Lee, Kai Li, Ran Lu, Thomas Macrina, Gayathri Mahalingam, Eric Mitchell, Shanka Subhra Mondal, Shang Mu, Barak Nehoran, Sergiy Popovych, Casey M. Schneider-Mizell, William Silversmith, Marc Takeno, Russel Torres, Nicholas L. Turner, William Wong, Jingpeng Wu, Szi-chieh Yu, Wenjing Yin, Daniel Xenes, Lindsey M. Kitchell, Patricia K. Rivlin, Victoria A. Rose, Caitlyn A. Bishop, Brock Wester, Emmanouil Froudarakis, Edgar Y. Walker, Fabian Sinz, H. Sebastian Seung, Forrest Collman, Nuno Maçarico da Costa, R. Clay Reid, Xaq Pitkow, Andreas S. Tolias, Jacob Reimer

**Affiliations:** 1 Center for Neuroscience and Artificial Intelligence, Baylor College of Medicine, Houston, TX USA; 2 Department of Neuroscience, Baylor College of Medicine, Houston, TX USA; 3 Department of Electrical and Computer Engineering, Rice University, Houston, TX USA; 4 Research and Exploratory Development Department, Johns Hopkins University Applied Physics Laboratory, Baltimore, MD USA; 5 Department of Ophthalmology, Stanford University, Stanford, CA USA; 6 Byers Eye Institute, Stanford University, Stanford, CA USA; 7 Stanford Bio-X, Stanford University, Stanford, CA USA; 8 Wu Tsai Neurosciences Institute, Stanford University, Stanford, CA USA; 9 Department of Mathematics, Creighton University, Omaha, NE USA; 10 Princeton Neuroscience Institute, Princeton University, Princeton, NJ USA; 11 Electrical and Computer Engineering Department, Princeton University, Princeton, NJ USA; 12 Allen Institute for Brain Science, Seattle, WA USA; 13 Computer Science Department, Princeton University, Princeton, NJ USA; 14 Brain and Cognitive Sciences Department, Massachusetts Institute of Technology, Cambridge, MA USA; 15 Institute of Molecular Biology and Biotechnology, Foundation for Research and Technology Hellas, Heraklion, Greece; 16 Department of Physiology and Biophysics, University of Washington, Seattle, WA USA; 17 UW Computational Neuroscience Center, University of Washington, Seattle, WA USA; 18 Institute for Bioinformatics and Medical Informatics, University Tübingen, Tübingen, Germany; 19 Institute of Computer Science and Campus Institute Data Science, University Göttingen, Göttingen, Germany; 20 Neuroscience Institute, Carnegie Mellon University, Pittsburgh, PA USA; 21 Department of Machine Learning, Carnegie Mellon University, Pittsburgh, PA USA; 22 Department of Computer Science, Rice University, Houston, TX USA; 23 Institute for Artificial and Natural Intelligence, Pittsburgh, PA USA; 24 Human-Centered Artificial Intelligence Institute, Stanford University, Stanford, CA USA; 25 Department of Electrical Engineering, Stanford University, Stanford, CA USA

**Keywords:** Neural circuits, Software, Data processing, Machine learning

## Abstract

We are in the era of millimetre-scale electron microscopy volumes collected at nanometre resolution^1,2^. Dense reconstruction of cellular compartments in these electron microscopy volumes has been enabled by recent advances in machine learning^3–6^. Automated segmentation methods produce exceptionally accurate reconstructions of cells, but post hoc proofreading is still required to generate large connectomes that are free of merge and split errors. The elaborate 3D meshes of neurons in these volumes contain detailed morphological information at multiple scales, from the diameter, shape and branching patterns of axons and dendrites, down to the fine-scale structure of dendritic spines. However, extracting these features can require substantial effort to piece together existing tools into custom workflows. Here, building on existing open source software for mesh manipulation, we present Neural Decomposition (NEURD), a software package that decomposes meshed neurons into compact and extensively annotated graph representations. With these feature-rich graphs, we automate a variety of tasks such as state-of-the-art automated proofreading of merge errors, cell classification, spine detection, axonal-dendritic proximities and other annotations. These features enable many downstream analyses of neural morphology and connectivity, making these massive and complex datasets more accessible to neuroscience researchers.

## Abstract (web summary)

Neural Decomposition (NEURD) is a software package that decomposes neuronal data from high-resolution electron microscopy volumes into feature-rich graph representations to facilitate analysis for neuroscience research.

## Main

To understand the morphological features of individual neurons and the principles governing their connectivity, the use of large-scale electron microscopy and reconstruction of entire neural circuits is becoming increasingly routine. For example, the Machine Intelligence from Cortical Networks (MICrONS) Consortium published a millimetre-scale open source dataset of mouse visual cortex^2^ (the MICrONS dataset, comprising approximately 80,000 neurons and 500 million synapses) and a team at Harvard published a similar reconstructed volume of human temporal lobe^1^ (the H01 dataset, comprising approximately 15,000 neurons and 130 million synapses). These reconstructions offer opportunities for analysis of neural morphology and synaptic connectivity at a scale that was previously inaccessible. However, effective use of these massive and complex datasets for scientific discovery requires a new ecosystem of software tools.

Here, we describe NEURD, a Python software package that extracts useful information from the 3D mesh and synapse locations for each neuron, and implements workflows for automated proofreading of merge errors, morphological analysis and connectomic studies. NEURD decomposes the 3D meshes of neurons from electron microscopy reconstructions into a richly annotated graph representation with many pre-computed features. These graphs characterize the neuron at the level of non-branching segments in the axonal and dendritic arbor and can support powerful queries spanning spatial scales from the geometry of the neuropil to the morphology of boutons and spines.

We begin by demonstrating the utility of this framework in an automated proofreading pipeline that is highly effective at correcting merge errors using heuristic rules. Hereafter, the term proofreading in reference to our pipeline will refer to merge error correction and not include the task of extending false splits. We focus on merge errors because of their catastrophic effects on connectivity analyses. We next show how the pre-computed features extracted by NEURD can enable us to recapitulate and extend a variety of previous observations about neural morphology and geometry, taking advantage of the diverse feature set computed on thousands of reconstructed neurons spanning all cortical layers in these volumes. Finally, we examine the potential of the NEURD workflow to yield novel scientific insights about neural circuit connectivity, including higher-order motifs in which we observe at least three nodes (neurons) and edges (synapses) in a specific graph arrangement. NEURD includes a fast workflow to identify axonal–dendritic proximities (regions where the axon of one neuron passes within a threshold distance of a postsynaptic dendrite).

Similar to other open source software packages that have supported the widespread adoption of other complex data modalities such as calcium imaging (CaImAn^7^ and Suite2P^8^), Neuropixels recordings (KiloSort^9^ and MountainSort^10^), label-free behavioural tracking (DeepLabCut^11^, MoSeq^12^ and SLEAP^13^) and spatial transcriptomics (Giotto^14^ and Squidpy^15^), the goal of NEURD is to make ‘big neuroscience data’ (in this case, large-scale electron microscopy reconstructions) accessible to a larger community. As more large-scale electron microscopy reconstructions become available, tools such as NEURD will become increasingly essential for exploring principles of neural organization across multiple species.

The following tables are provided in the supplementary information for further reference: Supplementary Table 1, cell-type subclass abbreviations and excitatory/inhibitory classification glossary; Supplementary Table 2, dataset sizes and relevant sample numbers for all figures and statistics; and Supplementary Table 3, a comprehensive guide to the pre-computed features provided by NEURD.

## Summary of large-scale reconstructions

Data collection for the MICrONS and H01 dataset has been described previously^1,2^. The tissue preparation, slicing procedure and imaging resolution (4–8 nm × 4–8 nm × 30–40 nm) was roughly similar in both cases. However, the imaging and reconstruction workflows for the two datasets were very different. The MICrONS volume was collected with transmission electron microscopy (TEM)^16^, whereas the H01 volume was collected with scanning electron microscopy (SEM)^17^, and different reconstruction pipelines were used^1,2,6^. However, all volumetric reconstructions produce similar 3D meshes as a common data product downstream of the segmentation process. The capabilities of NEURD are focused at the level of these mesh representations, which are much more lightweight than the original electron microscopy data, but still capture rich information about the microscale anatomy of neurons that can be useful for a variety of downstream analyses, including comparative analyses of neural circuitry across species, volumes and reconstructions.

## Preprocessing of neuronal meshes

**Fig. 1:**
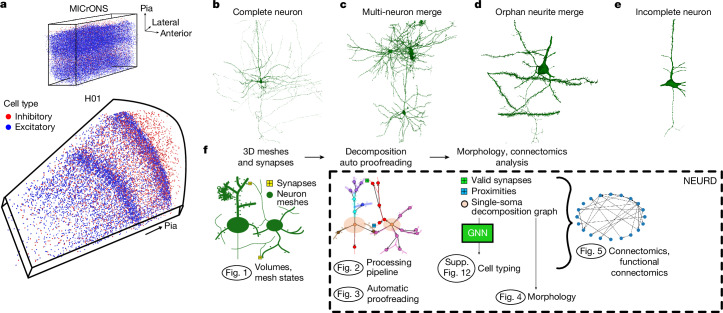
Working with neuronal meshes from large-volume electron microscopy segmentations. **a**, The MICrONS Minnie65 volume is an approximately 1,300 × 820 × 520 μm^3^ rectangular volume from mouse visual cortex; H01 is a wedge-shaped volume from human temporal cortex with a longest dimension of 3 mm, a width of 2 mm and a thickness of 150 μm. **b**–**e**, The range of accuracy across neural reconstructions in the MICrONS and H01 volumes. **b**, Example of a nearly complete (manually proofread) single neuron. **c**, A mesh containing two merged neurons from the MICrONS volume. **d**, Example of an orphan merge error with a piece of dendrite incorrectly merged onto a neuron mesh. **e**, An incompletely reconstructed neuron from the H01 volume. **f**, An overview of the NEURD workflow: starting from volumes and their initial mesh states (Fig. 1), to the processing pipeline (Fig. 2), automatic proofreading (Fig. 3), and cell typing (Supplementary Fig. 12), which then enables the analysis of morphology (Fig. 4), connectomics, and functional connectomics (Fig. 5).

**Fig. 2:**
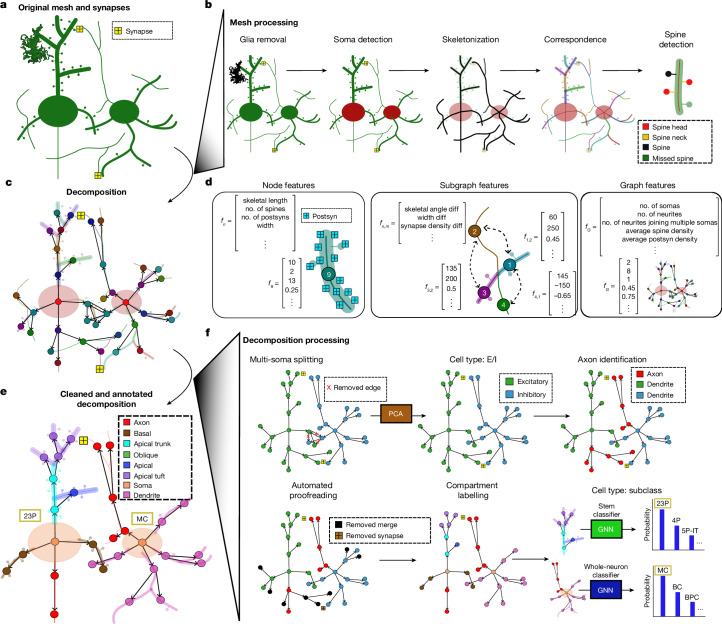
Decomposition, feature extraction and graph annotation. **a**, The input data (mesh and synapses) required for the NEURD workflow. **b**, The reconstructed meshes are pre-processed through a number of steps including decimation, glia and nuclei removal, soma detection and skeletonization. Mesh features are projected back onto the skeleton and spines are detected. **c**, Decomposition graph object composed of two neurons merged together. The decomposition compresses the skeleton, mesh and synapse annotations of a non-branching segment into a single node in a graph, with directed edges to the downstream segments connected at a branch point. The soma is the singular root node of this tree. **d**, NEURD automates computation of features at multiple levels. Node (non-branching segment)-level features include basic mesh characteristics (for example, diameter of the neural process or number of synapses per skeletal length). Subgraph features capture relationships between adjacent nodes such as branching angle or width differences. Graph features capture characteristics of the entire neuron and are computed by weighted average or sum of node features, or by counting subgraph motifs. Postsyn, postsyaptic region. **e**, The final product is a cleaned and annotated decomposition object with a single soma that can be fed into a variety of downstream analyses. **f**, NEURD supports a variety of operations and manipulations on the decomposition objects. Multi-soma splitting is performed with heuristic rules. The entire decomposition graph is classified as excitatory or inhibitory and one subgraph is identified as the axon. Automated proofreading is performed to remove probable merge errors (see Fig. 3). A set of heuristic rules is implemented to label neural compartments, followed by a finer-scale cell-type classification using graph neural networks (GNNs) (Supplementary Fig. 12). PCA, principal components analysis.

Electron microscopy reconstructions yield neural meshes with varying levels of completeness, and with different kinds of merge errors (Fig. 1b–e). Merge errors include multiple whole neurons connected together (Fig. 1c) and disconnected pieces of neurite (orphan neurites) merged onto different neural compartments (Fig. 1d). Merge errors may also include glia or pieces of blood vessels merged onto neurons (Fig. 1f). We take advantage of existing tools for mesh processing^18–21^ and apply them in an initial workflow that is agnostic to the identity of the mesh object (Fig. 2 and Supplementary Methods). Systematic inspection by manual proofreaders confirmed the high accuracy of the soma, axon, dendrite, glia, compartment and spine annotations generated during the mesh processing workflow (Supplementary Figs. 1 and  2).

## Graph decomposition

We decompose skeletons of axonal and dendritic processes into a directed tree graph (NetworkX object in Python^22^; we provide a step-by-step online tutorial on how to export these as SWC files). In these graphs, the root node is the soma and the other nodes are individual non-branching segments. Edges project downstream away from the soma, and subgraphs downstream of the soma are a stem. Multiple soma nodes are split apart if more than one soma is detected, and any cycles in the graph are broken during the decomposition process (Fig. 2f and Supplementary Methods). Previous work has emphasized the utility of this kind of graph representation of each neuron, which facilitates flexible queries and analyses of features and annotations at different scales^23,24^.

**Fig. 3:**
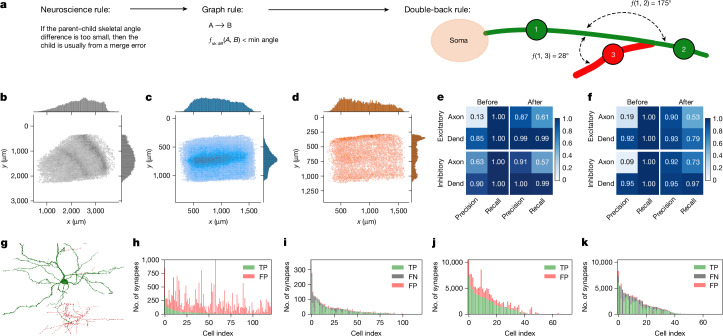
NEURD graph decomposition enables automated proofreading. **a**, Implementing domain knowledge as subgraph rules to automatically remove merge errors (see Supplementary Fig. 3 for more rules). **b**, Laminar distribution of merge errors (H01). The inhomogeneity of errors across different layers, possibly due to differences in neuropil density. The pial surface is to the right and slightly up (see Supplementary Fig. 7 for more details). **c**, Increased frequency of axon edits is observed in layer 5 of cortex (MICrONS). Pial surface is up. **d**, Dendritic errors in the MICrONS dataset increase near the top layers of the volume, where fine excitatory apical tufts lead to more frequent merges (see Supplementary Fig. 6 for more details). **e**,**f**, MICrONS (**e**) and H01 (**f**) synapse validation quantified by synapse precision and recall compared with manual proofreading (ground truth). ‘Before’ describes the accuracy of the raw segmentation prior to any proofreading. The substantial increases in precision ‘After’ automated proofreading (especially for axons) indicates that the cleaned neurons have good fidelity. The reduction in ‘After’ recall indicates the loss of some valid synapses in the automatic proofreading process (mostly concerning axons), while still retaining the majority of correct synapses (see also Supplementary Fig. 9 restricted to single somas). Dend, dendrite. **g**, An excitatory neuron from the MICrONS dataset in the 75th percentile of merge error skeletal length; identified merge errors are shown in red. **h**–**k**, Number of true-positive (TP) and false-positive (FP) axonal synapses from individual excitatory (**h**,**i**) or inhibitory (**j**,**k**) neurons in the validation set before (**h**,**j**) and after (**i**,**k**) automated proofreading, illustrating the large number of false-positive (red) synapses in the raw segmentation that are removed by automated proofreading (see Supplementary Figs. 8 and  9 for more details on the MICrONS dataset and Supplementary Fig. 10 for similar validation on the H01 dataset).

**Fig. 4:**
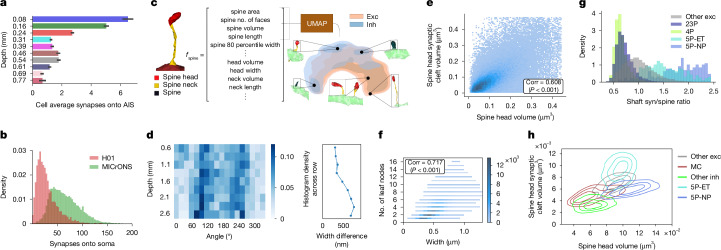
Morphological analysis enabled by NEURD feature extraction. **a**, Average number of synapses onto the AIS of cells at different laminar depths (mean ± s.d.) for MICrONS (*n* = 22,955). **b**, Distribution of the number of soma synapses per cell. As expected, neurons in the MICrONS volume have more identified synapses onto their soma, despite the smaller surface area compared to human somas (see Supplementary Fig. 16 for more AIS and soma synapse results). **c**, An example spine segmentation with the features extracted for each spine submesh followed by a kernel density estimation of the UMAP embedding of these features for spines sampled from the MICrONS dataset (see Supplementary Fig. 19 for more details). Exc, excitatory; inh, inhibitory. **d**, Histograms showing the distribution of the mean skeletal angle of the thickest basal stem as a function of volume depth (see Supplementary Fig. 17 for more details). **e**, Spine head synapse size and spine head volume joint distribution for the H01 dataset, showing a positive Pearson’s correlation coefficient (*P* < 10^−300^; see Supplementary Fig. 20 for more details). **f**, Histogram for all the non-apical dendritic stems of every neuron in the MICrONS volume comparing the initial width of the stem to the number of leaf nodes (blue scaling indicates the number of dendritic stems for a given bin), showing a positive Pearson’s correlation coefficient (corr; *P* < 10^−300^; see Supplementary Fig. 18 for more details). **g**, The ratio of non-spine synapses to spine counts varies across cell types. **h**, Distribution of spine characteristics for different cell types, comparing the volume of each spine head and the size of the largest synapse on that spine head, where outlines indicate the quartile boundaries for each distribution (see Supplementary Fig. 15 for more cell-type distributions). See Supplementary Table 2 for all associated *n* values.

**Fig. 5:**
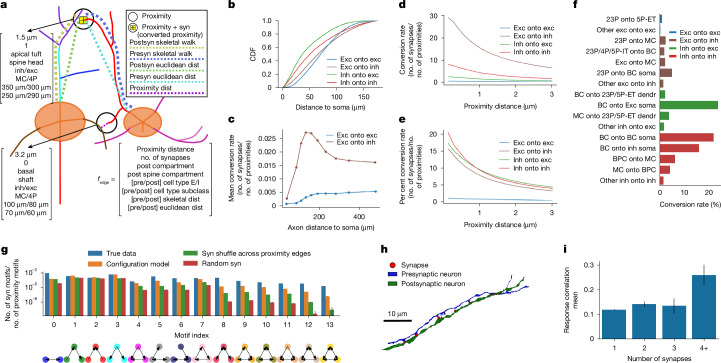
Connectivity analysis enabled by NEURD. **a**, Schematic illustrating two proximities between a pair of neurons (axon passing within 5 μm radius of a dendrite). Only one proximity has a synapse, thus the ‘conversion rate’ is 50%. **b**, Cumulative density function (CDF) of the postsynaptic dendritic skeletal walks for different connection types, demonstrating that excitatory inputs occur further along the dendrite from the soma (see Supplementary Fig. 23 for more details). **c**, Mean conversion rate as a function of distance along the axon (see Supplementary Fig. 23 for more details). **d**,**e**, Conversion rates (synapses/proximities) for different excitatory and inhibitory combinations. The *x*-axis represents the maximum distance that is considered a proximity. **f**, Conversion rates for different cell-type subclasses and compartments are largely consistent with previous studies (MICrONS; cell-type labels generated from a GNN classifier; see Supplementary Table 1 for glossary and Supplementary Fig. 24 for more conversion rates). **g**, The frequency (mean ± s.d.) of reciprocal connections or edge-dense three-node motifs was enriched compared with null distributions where synaptic degree distribution is held the same but edges are shuffled (orange), where the synaptic edges are shuffled across existing proximity edges (green) or where synapses are randomly shuffled (red); 250 random graph samples for each null distribution comparison (see Supplementary Fig. 25 for more details and inhibitory/excitatory-only graphs). **h**, Example multi-synaptic connection (*n* = 7 synapses) from an excitatory to inhibitory neuron (H01). **i**, Distribution of response correlation mean (±s.e.m.) between pairs of functionally matched excitatory neurons (MICrONS). Response correlation is significantly larger for pairs of neurons with 4 or more synapses connecting them (*n* = 11 pairs) compared with those with 1, 2 or 3 synapses (*n* = 5,350, 280 or 34 pairs, respectively; see Supplementary Fig. 27 for more details). See Supplementary Table 2 for all associated *n* values.

NEURD computes a large number of features at the branch (node), stem (subgraph) or whole-neuron (graph) level (Fig. 2c,d). These multi-scale features make it straightforward to translate neuroscience domain knowledge into neuron or compartment-level operations and queries. The most important context for this translation is automatic proofreading (Fig. 3), and NEURD also includes workflows for common tasks such as cell-type classification (Supplementary Fig. 12), morphological analysis (Fig. 4) and connectivity analysis (Fig. 5).

## Automated proofreading

Node, graph and subgraph features can be queried to identify patterns of features that are commonly found at merge errors in the reconstruction (for example, sharp discontinuities in width between adjacent dendritic nodes, or axonal branches that ‘double-back’ sharply towards the soma). Once the error location is identified, all nodes downstream of the error are stripped from the mesh and returned to the ‘sea of unconnected fragments’ in the volume, excluding them from any subsequent analysis. The NEURD proofreading workflow is easily extensible, and the user can define which graph filters to apply in which order. For any error correction, the rule and relevant parameters that determined the edit are stored for subsequent evaluation and use, potentially providing a rich set of training data for machine learning approaches.

To illustrate this process, we provide a small set of heuristic proofreading rules implemented as graph filters (Fig. 3a, Supplementary Fig. 3 and Supplementary Methods) that yielded good performance on merge error correction in both volumes, but especially in the MICrONS dataset. Manual validation of these rules was performed in the context of standard proofreading and multi-soma splitting using the NeuVue Proofreading Platform^25^ by the proofreading team at Johns Hopkins University Applied Physics Laboratory (APL). We provided APL with suggested error locations in the MICrONS volume, and experienced proofreaders evaluated each proposed split for accuracy (Supplementary Fig. 4a–c). This validation set included multi-soma splitting, axon-on-axon and axon-on-dendrite merge errors, and enabled us to measure both the accuracy of these proofreading rules and the speed benefits of a semi-supervised approach compared to a fully manual effort. We were also able to optimize these rules on the basis of proofreader feedback, and we identified specific rules and parameter thresholds could be applied with high confidence to correct merge errors without human intervention. Validation of this ‘high-confidence’ subset of axon-on-axon merges and axon-on-dendrite merges yielded a 99% and 95% agreement between the algorithm’s split operations and those performed by a human proofreader (Supplementary Fig. 4e). We applied nearly 150,000 of these high-confidence automatic edits in bulk to the publicly available MICrONS segmentation volume. Using NEURD suggestions in a semi-supervised manner to guide the challenging process of splitting multi-soma segments increased the speed of this process more than threefold compared with manual methods (Supplementary Fig. 4d and Supplementary Methods).

In the MICrONS dataset, we identified hundreds of thousands of merge errors corresponding to dozens of metres of incorrectly merged axons and dendrites within the 1 mm^3^ volume (Supplementary Fig. 5). Corrections in the H01 dataset were an order of magnitude smaller owing to fewer cells and the less complete initial reconstructions in that volume, but were still substantial (Supplementary Fig. 5). Merge errors were more prevalent in some regions owing to sectioning artifacts (Supplementary Figs. 6g–l and 7h–n) or to intrinsic differences in the morphology of neurons across layers (Fig. 3b–d and Supplementary Figs. 6a–f and 7a–g). For example, high- and low-degree axon edits in the MICrONS dataset were frequently made in upper layer 5, potentially owing to higher quantities of inhibitory neuropil, whereas dendritic double-back and width jump errors were more frequently located near the top layers of the volume, owing to merges between fine distal apical tufts of excitatory cells (Supplementary Figs. 6 and  7).

We compared the outcome of automatic proofreading (all edits, not just the high-confidence subset) to manual proofreading ‘ground truth’ on a test set of cells in the MICrONS (*n* = 122 excitatory and *n* = 75 inhibitory) and H01 (*n* = 49 excitatory and *n* = 18 inhibitory) volumes (see Supplementary Fig. 30 for manually proofread cell locations).

The precision of the synaptic data—that is, the number of actually true synapses that were labelled as true divided by the total number of synapses labelled as true—was substantially higher after proofreading (for example, 0.87 after compared to 0.13 before for MICrONS excitatory axons, a more than sixfold increase). For the same axons, this increase in precision was achieved with only a 40% reduction in recall (number of correctly identified synapses divided by the number of true synapses). Low precision can be catastrophic for downstream analyses, whereas low recall can support many analyses on the basis of the assumption that the observed synapses are a representative sample of the full distribution (precision and recall are summarized in Fig. 3e,f and Supplementary Figs. 8–10). The precision and recall of our automated method is captured visually in the plots in Fig. 3h–k. Comparing ‘before’ and ‘after’ proofreading performed with NEURD, red false positives are almost entirely removed, at the cost of a thin margin of false negatives (grey) cutting into the green true positives. For example, for the axons of the the 122 excitatory neurons in the MICrONS dataset, NEURD correctly removed 24,430 false synapses and incorrectly removed 1,420 true synapses, leaving 2,216 true synapses and 324 false synapses. Full numbers are available in Supplementary Table 2.

Because our automated proofreading procedure only removes data, recall is measured on the basis of the true synapses in the automatic segmentation (after merge errors were removed manually), and does not include synapses from any manual extensions. Recall was especially high for dendrites (99% for MICrONS single-soma neurons; Supplementary Fig. 9), reflecting the high performance of the initial segmentations. Overall recall was lower for axons (approximately 60% for both excitatory and inhibitory cells in the MICrONS dataset), indicating that NEURD incorrectly removed a larger number of axonal segments compared to dendrites. Performance on the H01 dataset was also reduced compared with MICrONS, because the less-extensive reconstruction was associated with fewer merge errors overall. Extensive, sometimes centimetre-scale arbors remained after removing merge errors (Fig. 3g and Supplementary Fig. 11). In summary, both from the perspective of synapses (Fig. 3h–k and Supplementary Figs. 8i–p and  10i–p) and skeletons (Supplementary Figs. 8e–h and  10e–h), our automated proofreading approach can be applied at scale to remove merge errors with accuracy approaching that of manually cleaned cells (Supplementary Fig. 11).

## Cell-type classification

Densely reconstructed electron microscopy volumes hold great promise for understanding the connectivity between different neural subtypes^26–32^. Because electron microscopy provides limited access to genetic markers, cell types must be identified by morphological features or connectivity (if sufficient proofreading is performed). The relationship with molecularly defined cell classes can sometimes be inferred from extensive previous work relating morphological features to transcriptomic classes^33–35^, including from our consortium^36^. Previous studies have demonstrated that rich information enabling cell-type classification is available even in local nuclear and peri-somatic features^37,38^, small segments of neural processes^30^, views of single-nuclei segments at different resolutions^39^ and the shape of postsynaptic regions^40^. NEURD provides an additional rich and interpretable feature set at the level of non-branching segments that can be used for accurate cell-type classification via a number of different approaches, as we describe below.

As expected^41^, we found that a logistic regression model trained on just two spine and synapse features separates excitatory and inhibitory cells with high accuracy, using the same parameters for classification across both the MICrONS and H01 dataset (Supplementary Fig. 12a,b; MICrONS, *n* = 3,985 excitatory and *n* = 897 inhibitory; H01, *n* = 5,800 excitatory and *n* = 1,755 inhibitory). To test whether NEURD graph objects could be used to distinguish even finer cell types, we turned to graph convolutional networks (GCN) (Supplementary Fig. 12c–f). We trained a simple GCN on the dendritic subgraph of a variety of hand-labelled cell types in the MICrONS volume (*n* = 873 total cells), which represent a relatively complete set of cell-type classes for this volume and are more thoroughly described in refs. ^26,37^. We focused on the dendrites in this volume because of their high recall from the initial segmentation and the high precision after automated proofreading (Fig. 3f). Most of the embedding space was covered by the labelled dataset (Supplementary Fig. 12c) and cells outside the labelled dataset had soma centroids at expected laminar depths (Supplementary Fig. 12d), even though no coordinate features were used in the GCN classifier.

We evaluated cell-type classification performance on a held-out test set using a GCN with access to the entire dendritic graph (*n* = 178 test neurons; mean class accuracy = 0.82; class accuracy: layer 2/3 pyramidal (23P, 0.94), layer 4 pyramidal (4P, 0.69), layer 5 intratelencephalic (5P-IT, 0.60), layer 5 near-projecting (5P-NP, 1.00), layer 5 pyramidal track (5P-ET, 0.86), layer 6 corticothalamic (6P-CT, 0.58), layer 6 intratelencephalic (6P-IT, 0.62), basket cell (BC, 0.85), bipolar cell (BPC, 0.89), Martinotti cell (MC, 1.00) and neurogliaform cell (NGC, 1.00); Supplementary Fig. 12e, also see actual counts for training, validation and test in Supplementary Fig. 14a–c). We also evaluated the classification performance using only disconnected dendritic stems (*n* = 1,023 test stems; mean class accuracy = 0.66; class accuracy: 23P (0.73), 4P (0.78), 5P-IT (0.33), 5P-NP (0.78), 5P-ET (0.78), 6P-CT (0.56), 6P-IT (0.49), BC (0.80), BPC (0.70), MC (0.79) and NGC (0.50); Supplementary Fig. 12f; counts for training, validation and test in Supplementary Fig. 14d–f). The information present in disconnected individual dendritic stems (branching segments connected to the soma) is thus sufficient to perform fine cell-type classification nearly as well as graphs representing entire neurons, consistent with previous literature classifying cells based on more local features^30,37,38^ (the cell-type abbreviation glossary is provided in Supplementary Table 1). Because the classifier is a deep learning model, the output from the final softmax layer can be used as a confidence measurement, making it possible to restrict downstream analyses to high-confidence cell-type labels.

## Morphological analysis

The features extracted by NEURD—including features of different compartments (Supplementary Fig. 19a), the geometry of axonal and dendritic compartments (Supplementary Fig. 17a) and spine features (Supplementary Fig. 19b)—provide a rich substrate for morphological analysis (Supplementary Fig. 15).

In particular, extensive work has linked spine morphology to synaptic strength and stability, making them important targets for understanding plasticity and connectivity in neural circuits. A variety of methods have been developed to automate spine detection in 2D or 3D image data using fully automatic^42–46^ or semi-automatic^47^ approaches. NEURD offers an accurate spine detection workflow that achieves high accuracy with a fully automated mesh segmentation approach. Precision and recall for spines with a skeletal length larger than 700 nm was 90% or higher (Supplementary Fig. 1). In addition, NEURD segments the spine head from the neck (when possible) and computes statistics about the individual head and neck submeshes, creating a feature-rich dataset for testing hypotheses about spine morphology that can then be conditioned on postsynaptic compartment type or the cell type of the presynaptic or postsynaptic cell. As expected, the spine head volume and synaptic density volume were the only strongly correlated spine features^48,49^ (Supplementary Fig. 20). The spatial distribution of uniform manifold approximation and projection for dimension reduction (UMAP) embeddings (2D projection) for feature vectors of spines sampled from the MICrONS and H01 dataset showed a similar structure, with spines that share similar features embedded in similar locations and a somewhat consistent embedding pattern for inhibitory and excitatory spines in the two volumes. This similarity suggests that H01 and MICrONS spines sample from a similar landscape of diverse spine shapes (Supplementary Fig. 19c,d), consistent with previous work examining the distribution of non-parametric representations of postsynaptic shapes across diverse neural subtypes^40^.

We attempted to replicate and extend several other findings observed in previous studies of the MICrONS and H01 datasets regarding the subcellular targeting of synaptic inputs. First, we computed the number of synapses onto the axon initial segment (AIS) of neurons at different depths. Replicating a previous report, in the MICrONS volume, superficial L2/3 pyramidal cells received the largest number of AIS synapses, with up to 2 to 3 times the innervation of the lower cortical layers^27,50,51^ (Fig. 4a). However, in the H01 dataset, this laminar inhomogeneity in AIS synapses was much less prominent, with more similar numbers of AIS inputs observed across all depths (Supplementary Fig. 16h). Additionally, similar to AIS synapses, we found a marked difference in the distribution of somatic synapses across depth between the MICrONS and H01 dataset (Supplementary Fig. 16e,f). Finally, the overall frequency of somatic synapses were also distinct across the two volumes, consistent with previous literature describing fewer somatic synapses in the human compared to mouse^52^ (Fig. 4b); however, we found the opposite trend for the AIS, with fewer AIS synapses in the mouse volume compared with the human volume (Supplementary Fig. 16i).

In H01, deep layer pyramidal cells were previously observed to have a strong bias in the radial angle of their thickest basal dendrite^1^. We examined the MICrONS volume and did not observe a strong bias in thickest basal, even in deep layers (Supplementary Fig. 17b). Then, looking at H01, we were able to replicate the pattern of thickest basal dendrite direction preferences in deeper layers (Supplementary Fig. 17c). However, we also found that this pattern appeared to continue into more superficial layers, extending the previous finding. In deep layers, the difference between the thickest and second-thickest dendrite was nearly twice the difference in more superficial layers, making this effect more salient.

The diversity of pre-computed features offered by NEURD enabled us to identify several interesting morphological features that differ across cell types, including many that have been reported previously in other studies. For example, the spindly, non-branching basal dendrites of near-projecting (NP) cells^26,29^ are clearly distinct from extensively branching basal dendrites of L2/3 pyramidal cells (Supplementary Fig. 15h), and neurogliaform cells are the most highly branched neurons with the largest number of leaf nodes (Supplementary Fig. 18a). Across all neurons, dendritic stems with larger numbers of leaf nodes had a larger initial dendritic diameter at their connection to the soma (Supplementary Fig. 18b,c), potentially reflecting developmental or metabolic constraints.

Synapses onto the dendritic shaft and synapses onto dendritic spines roughly correspond to inhibitory versus excitatory inputs^53–55^. In a histogram of shaft to spine synapses, NP cells were again located at the higher end of the distribution, whereas L4 and L2/3 pyramidal cells had the lowest shaft-synapse to spine-synapse ratio (Fig. 4g), suggesting that they receive a relatively larger fraction of excitatory (compared with inhibitory) input. Because NEURD also automatically segments both soma meshes and spine heads and necks, this enables comparison across cell types of features such as soma volume and somatic synapses (Supplementary Fig. 15b,f), spine neck length (Supplementary Fig. 15i), spine density (Supplementary Fig. 15a) and the relationship between spine synapse size and spine head volume, as in Fig. 4h and Supplementary Fig. 20.

## Connectivity and proximities

Next, we examined the connectivity graph in the MICrONS and H01 datasets after automatic proofreading. As expected, removal of false merges substantially reduced the mean in-degree (number of incoming synapses onto a neuron) and out-degree (number of projecting synapses from a neuron) across both volumes owing to the removal of merge errors, resulting in a sparsely sampled but high-fidelity graph (Fig. 3e,f). A variety of connectivity statistics including number of nodes and edges, mean in and out degrees, and mean shortest path between pairs of neurons along excitatory and inhibitory nodes are provided in Supplementary Fig. 22.

To facilitate the analysis of synapse specificity in sparse connectomes, we implemented a fast workflow for identifying axonal–dendrite proximities, regions where the axon of one neuron passes within a threshold distance (here 5 μm) of the dendrite of another neuron (Fig. 5a and Supplementary Methods). Previous studies have computed proximities from skeletons of simulated models^56^ or manually traced data^57,58^ with a similar logic. Proximities are necessary but not sufficient for the formation of a synapse^31,57,59,60^, and so the ‘proximity graph’ can serve as a valuable null distribution for comparing potential connectivity with synaptic connectivity between neurons: instead of looking at synapse counts between cells, which are dependent on the geometry and completeness of the neuropil, proximities make it possible to calculate ‘conversion rates’—the fraction of proximities that resulted in actual synaptic connections. NEURD also provides functions to compute ‘presyn skeletal walk’—the distance from a synapse to the soma of the presynaptic neuron along the axon, and ‘postsyn skeletal walk’—the distance from synapse to soma along the postsynaptic dendrite. Combined with cell typing, compartment labelling and spine annotation, these features enable powerful analyses of neural connectivity conditioned on the cellular identity and subcellular location of synapses on both pre- and postsynaptic partners (Supplementary Figs. 6 and 26).

Conversion rates between neural subtypes in the MICrONS dataset replicated previous results from connectivity measured via slice multi-patching and electron microscopy reconstructions, especially the prolific connectivity of basket cells onto both excitatory and inhibitory somas^26,61–63^ (Fig. 5f) and inhibitory–inhibitory relationships, including BCs inhibiting other BCs, MCs avoiding inhibiting other MCs, and BPCs preferentially inhibiting MCs^26,61,62,64^ (Supplementary Fig. 24).

The subcellular targeting of different inputs is apparent in plots of the postsyn skeletal walk distance to the soma for synapses arriving at the basal dendrite. As has been previously described^53,65,66^, inhibitory-onto-excitatory synapses tend to be found closer to the somatic compartment than excitatory-onto-excitatory synapses (Fig. 5b and Supplementary Fig. 23g). At an even smaller scale, with the spine head, spine neck or shaft classification propagated to synapses, we can study how excitatory and inhibitory inputs to spines display different scaling relationships between synapse size and spine head volume (Supplementary Fig. 21). We also show, as expected, that excitatory and inhibitory cells differ in the number and relative sizes of synapses on their target spine heads^66,67^ (Supplementary Fig. 21).

Conversion rates for excitatory-to-excitatory proximities were low in both H01 and MICrONS, consistent with previous findings of sparse pyramidal cell connectivity in the cortex^57,58,61,68^ (Fig. 5d,e). However, conversion rates were substantially higher for excitatory-to-inhibitory proximities (Fig. 5d,e), especially in H01, and especially for proximity distances less than 2 μm (unlike excitatory synapses onto excitatory cells, where spines presumably reduce the dependence on distance). Combining the presynaptic (axonal) skeletal walk features and proximity analyses revealed an interesting similarity in excitatory-onto-inhibitory connectivity between the MICrONS and H01 datasets, with conversion rates peaking in the more proximal axon a few hundred micrometres from the soma^30,69–71^ (Fig. 5c and Supplementary Fig. 23e), a pattern that could reflect a lateral inhibition motif. Conversion rates were also higher above (more superficial to) the presynaptic soma than below (deeper than) the presynaptic soma for excitatory-onto-inhibitory connections in both volumes (Supplementary Fig. 23b,c).

Large-volume electron microscopy connectomics offer tremendous potential opportunities to examine higher-order motifs on a large scale. We found that more densely connected triangle motifs were enriched in the MICrONS volume compared with several controls (Fig. 5g and Supplementary Fig. 25 for excitatory and inhibitory subgraphs). The over-abundance of densely connected triangle motifs that we observed is consistent with previous findings suggesting that this higher-order organization is enriched in the cortex^27,56,72–74^. A similar pattern was observed in the H01 dataset, consistent with previous modelling of connections and proximities in the dataset^56^. However, in the H01 volume several of the three-node motifs with larger numbers of connected edges were missing owing to the less complete reconstruction (Supplementary Fig. 25).

## Functional connectomics

A key advantage of the MICrONS dataset is the functional characterization of matched neurons in vivo prior to electron microscopy data collection. The relationship between function and synaptic connectivity is covered in detail in a separate paper^75^, which includes analysis using automatically proofread data from NEURD. Here, we aimed to provide an illustration of how automated proofreading can enable a specific functional connectomics analysis that would otherwise be very challenging. We identified pairs of excitatory neurons connected by one, two, three, or four or more synapses. Querying for these rare high-degree connections between pyramidal cells was only possible after automated proofreading, since of the original 10,000-plus pairs with four or more connections, approximately 97% were identified as merge errors during automatic proofreading. Connections were further restricted to synapses onto postsynaptic spines to guard against possible missed merge errors where an inhibitory axon segment might still be merged to an excitatory neuron. Examples of these multi-synaptic connections have been highlighted in the H01 dataset (Fig. 5h), and rare examples can also be found in the MICrONS dataset^76^ (Supplementary Fig. 27b). We were able to identify *n* = 11 of these pairs in exclusively automatically proofread neurons (no manual proofreading), where both neurons also had been characterized functionally (Supplementary Fig. 27c). The average response correlation was calculated for each group of pairs^75,77^ (Supplementary Methods). We found that neurons with 4 or more synapses had significantly higher response correlations (0.259 ± 0.042 (*n* = 11)) to visual stimuli than neurons with fewer than 4 synapses (1 synapse: 0.118 ±  0.002 (*n* = 5,350); 2 synapses: 0.140 ± 0.011 (*n* = 280); 3 synapses: 0.133 ± 0.030 (*n* = 34)) connecting them (Fig. 5i), consistent with a Hebbian ‘fire together–wire together’ rule governing high-degree connectivity in the cortex, and this same pattern was also observed for *n* = 12 pairs of manually proofread neurons. For autoproofread neurons, two-sample Kolmogorov–Smirnov test and *t*-test for comparing each multi-synaptic group’s null likelihood of being drawn from the same distribution as the 1-synapse group: 2 synapses Kolmogorov–Smirnov test statistic = 0.068, *P* = 0.17 and *t*-test *P* < 0.03; 3 synapses Kolmogorov–Smirnov test statistic = 0.139, *P* = 0.49 and *t*-test *P* = 0.60; 4 synapses Kolmogorov–Smirnov test statistic = 0.508, *P* < 10^−2^ and *t*-test *P* < 10^−2^ (and still significant after Bonferroni correction for multiple comparisons with a significance threshold of *P* < 0.02).

## Discussion

NEURD is an end-to-end automated pipeline that is capable of cleaning and annotating 3D meshes from large electron microscopy volumes and pre-computing a rich set of morphological and connectomic features that are ready for many kinds of downstream analyses. Building on existing mesh software packages, NEURD adds a suite of neuroscience-specific mesh functions for soma identification, spine detection and spine segmentation that are applicable across multiple datasets, as well as workflows for skeletonization and mesh correspondence that complement existing tools. We demonstrate the utility of this integrated framework for morphological (Fig. 4 and Supplementary Figs. 15–20) and connectomic analyses (Fig. 5 and Supplementary Figs. 21–27), most of which involved queries against pre-computed features, as well as demonstrating how these features can be combined to ask new questions (Fig. 4h and Supplementary Figs. 16i,j, 21g,h, 23d,e and 27d). The set of features generated by NEURD is easily extensible, and our hope is that NEURD will make these daunting datasets more accessible for a larger group of researchers.

Several previous studies have proposed post hoc methods for automated proofreading including merge and split error detection and correction. Some of these methods make use of convolutional neural networks (either supervised^78,79^ or unsupervised^80^), reinforcement learning methods^81^ or other machine learning approaches^82–84^. Others make use of heuristic rules applied to neural skeletons^85,86^, and at least one approach uses both skeleton heuristics and convolutional neural networks^87^. Compared with automated methods applied directly to the electron microscopy segmentation that may be heavily memory-intensive, NEURD benefits from the lightweight features computed from mesh representations, enabling analysis to scale to larger volumes more easily. Methods based on the electron microscopy segmentation have the advantage that intracellular features can be used to aid proofreading, and these methods could potentially still be utilized upstream of NEURD. A key strength of the coarse-scale graph with pre-computed annotations is the flexible querying across multiple scales. For instance, distinguishing whether a thin, aspiny projection from a dendrite is the true axon or a merge error might require both local features and also additional information about the neuron type, the distance from the soma, and the spine density of the parent branch.

Our present implementation does not address some types of errors in automated segmentation. For example, it cannot presently handle merge errors that result in a co-linear segment of skeleton that we interpret as a single non-branching segment. Second, because the present implementation of NEURD is focused on removing false merges, it is unable to fix incomplete neural processes (Supplementary Fig. 8c). Motivated by the low recall of many neurons in these volumes, we plan to try using NEURD to perform automated extensions in the future by ‘over-merging’ candidate segments at a truncation point, and then correcting the resulting ‘merge error’ to choose the best candidate for extension.

We performed extensive validation of our automated proofreading approach to determine the precision and recall of our error correction, but as a general disclaimer it is important to note that some of the results that we have presented here might change if the same neurons were manually proofread and fully extended. For any particular scientific question, researchers using these volumes will need to weigh the relative importance of precision, recall and the number of neurons that it is feasible to proofread using manual or automated methods. Finally, our results currently present findings from only two mammalian volumes, but there is nothing in principle preventing the application of NEURD to large electron microscopy volumes from other species in the future.

Combining automated proofreading to generate a clean but incomplete graph with proximities to serve as a null distribution is a powerful approach that can begin to reveal principles of pairwise and higher-order connectivity motifs in incomplete reconstructions. We observe a general overexpression of densely connected triangle motifs in comparison to proximity controls and some standard null models, as previously reported^56,72–74^. However, the ability to expand this work to include cell-type colourings of these motifs and add proximity-based controls will enable investigation of more complicated motif questions, unleashing the power of these datasets for addressing questions about higher-order circuit connectivity. As additional reconstructed volumes are released spanning species and brain areas, our ability to extract scientific insights will depend critically on integrated and scalable frameworks that make neurons and networks accessible for analysis, with a rich feature space suitable for machine learning approaches to understanding these complex datasets.

### Reporting summary

Further information on research design is available in the Nature Portfolio Reporting Summary linked to this article.

## Online content

Any methods, additional references, Nature Portfolio reporting summaries, source data, extended data, supplementary information, acknowledgements, peer review information; details of author contributions and competing interests; and statements of data and code availability are available at 10.1038/s41586-025-08660-5.

## Supplementary information

Supplementary Information This file contains Supplementary Methods, Supplementary Figs. 1–30 and Supplementary Table 1.

Reporting Summary

Supplementary Table 2 Figure data size (*N*). Table that gives more thorough details on the *N* size used in all experiments/plots of every figure in the main figures and the supplementary figures (as well as subclass *N* size, *P* values for Pearson’s correlation coefficient when relevant).

Supplementary Table 3 Neuron feature documentation. Describes the numerous features computed by the NEURD pipeline, documenting the resolution of the statistic, how to access, and at what point in the decomposition process the statistic is computed/available.

## Data Availability

All MICrONS data have already been released on BossDB (https://bossdb.org/project/microns-minnie; also see https://www.microns-explorer.org/cortical-mm3 for details). All H01 data are public, with access instructions on their homepage (https://h01-release.storage.googleapis.com/data.html). Additionally, packages such as NAVIS exist to facilitate easier access to both datasets. Because all datasets are publicly available and the NEURD package is publicly available with extensive tutorials on how to run each stage in the pipeline, all data products of interest (used in the study and figures) can be reproduced on demand and thus are not provided through separate public API. However, for convenience, some data products (such as synapse spine labels and cell-type subclasses) are publicly available through CAVE.

## Code availability

Code, documentation and example tutorials are available at https://github.com/reimerlab/NEURD.git.
