# Supplementary material for: NEURD offers automated proofreading and feature extraction for connectomics
Source: Nature. 2025 Apr 9;640(8058):487–96. doi: 10.1038/s41586-025-08660-5 (PMC11981913; doi:10.1038/s41586-025-08660-5)
Supplement: Supplementary file 1 — This file contains Supplementary Methods, Supplementary Figs. 1–30 and Supplementary Table 1. [file 41586_2025_8660_MOESM1_ESM.pdf]

nature portfolio

https://doi.org/10.1038/s41586-025-08660-5

Supplementary information

NEURD offers automated proofreading and feature extraction for connectomics

In the format provided by the authors and unedited

Supplemental Methods

Data Management. For simplified data management and querying of input neuron reconstruction meshes, NEURD intermediate decomposition graphs, and all derived statistics and data products, we utilized the DataJoint Python package [1,2]

Mesh Preprocessing. NEURD operates on 3-D meshes which are represented in a standard form as lists of vertices and faces in 3-D coordinates. A connected mesh component is a set of faces and vertices in which all faces have at least one adjacent edge to another face. Segmentation algorithms may not output a single connected component as a mesh, but instead may generate several disconnected submeshes, each of which is a subset of faces that is a connected component. NEURD is generally robust to discontinuous meshes, meshes of different resolutions, and several kinds of meshing errors.

The resolution of meshes delivered as part of the MICrONS and H01 datasets was sufficiently high that we performed an initial decimation of the mesh (reduced to 25% for MICrONS and 18% for H01) to speed up subsequent computations while retaining all the detail necessary for morphological characterization even of fine axons and spine necks. This decimation was performed using the MeshLab Quadric Edge Collapse Decimation function [3]. Following decimation, we separated this decimated mesh into connected components. We next applied a Poisson Surface Reconstruction [3] to each connected component. This can be thought of as "shrink-wrapping" the mesh - it smooths discontinuities on the surface of the mesh and ensures that each connected component is "water-tight" (i.e. no gaps or missing faces). This pre-processing facilitates the subsequent decomposition steps.

Glia, Nuclei Removal. Glia and nuclei submeshes are identified and filtered away using ambient occlusion functions [3] to identify regions with a high density of inside faces. Inside faces are mesh faces that are almost fully surrounded by other mesh faces. For example, glia that are merged onto neurons appear as cavities filled with a high density of mesh faces, and are distinct from the hollow reconstructions of most excitatory and inhibitory neurons. Similarly, the mesh representation of the soma surrounds the nucleus mesh and thus nuclei are almost entirely made up of inside faces. Therefore to identify glia and nuclei in the reconstructed meshes, we look for large connected components with high percentages of inside faces as candidates. To determine whether mesh faces are internal or external, we simulate an external "light source" that emits from all angles and we compute the exposure each face receives. This metric is thresholded to classify faces as either inside or outside faces, and submeshes made up mostly of inside faces are candidates for removal. We then apply additional thresholds on the candidate submeshes volume and number of faces to classify them as a glia mesh, nuclei mesh or neither. Finally, for glia we include all floating meshes within the bounding box of the submesh or within a search radius (3000 nm) of any faces of the submesh. This post-hoc mesh agglomeration serves to clean up the areas around glia segmentation, which can be very unconnected and non-standard.

Soma detection. Soma detection is run on any segment containing at least one detected nucleus (note that nucleus detection was performed previously as part of the segmentation and annotation workflow Consortium et al. [4], Shapson-Coe et al. [5]). To detect the soma, we first perform a temporary heavy decimation of the mesh to remove small features and facilitate detection of the large somatic compartment. We then segment this low-resolution mesh into contiguous submeshes using the CGAL mesh segmentation algorithm [6]. This function not only provides the specific faces in each submesh but also an estimate of the width of the submesh as an SDF value (Shape Diameter Function, a measure of diameter at every face of the mesh surface Yaz and Loriot [6]). We then filter all the resulting submeshes for soma candidates by restricting to those within a set size (number of faces), SDF range, bounding box length and volume threshold. We restrict to candidates that are sufficiently spherical to represent the general shape of a soma, but liberal enough to account for somas that are partially reconstructed (for example at the edge of the volume). Once we identify candidate somas in the low-resolution mesh, we return to the original mesh representation (prior to starting soma detection) and apply a final size and width threshold. Given the initial restriction to segments with at least one detected nucleus, if we are not able to detect at least one soma after this process, we relax the thresholds slightly and iterate until a soma is detected or a threshold limit for number of attempts is reached.

Decomposition. With the glia and nuclei submeshes identified, we filter those away from the original mesh, which may cause splitting into additional connected components. We identify connected components containing at least one soma submesh (note that some segmentations may contain multiple somas prior to soma splitting). Mesh fragments that are not connected to somas may be floating meshes inside the soma (which are filtered away using the same ambient occlusion methods described for glia above), or detached mesh pieces of neural processes that can be stitched to the neuron representation later. For each of the soma-containing meshes we filter away the soma submeshes and identify connected components of these meshes as stems. Any stem submesh must contain at least one set of connected adjacent edges and common vertices shared with a soma submesh ("border vertices"). We construct a connectivity graph where edges only exist between stem and soma nodes if there exists border vertices between the stem and the soma. Through the graph constructed in this manner, stems that provide paths between multiple somas can be easily identified for subsequent splitting (see below).

Each of the stem submeshes is then processed into a skeleton - a 3-D "line-segment" representation that is a set of vertices and edges. We use the Meshparty package for this initial round of skeletonization because of its efficient implementation, and

because it provides both a width estimate and a correspondence between the faces of the original mesh and each vertex in the skeleton[7]. This skeleton is then further divided into branches (non-branching subskeletons). The corresponding meshes of branches with an average width greater than a threshold are re-skeletonized with a higher-fidelity method that yields skeletons which pass through the hollow centers of the mesh to provide a better estimation of the location and curvature of the surrounding mesh. This is particularly important for some neural processes, for example wide apical trunks where a skeleton that is not centered within the mesh could be displaced nearly a μm from the actual center of the trunk. This higher-fidelity method is performed using the CGAL Triangulated Surface Mesh Skeletonization algorithm[8].

For mesh correspondence and width determinations of all skeletons, the NEURD algorithm employs a custom mesh correspondence workflow based on the following steps: First, each non-branching segment of the skeleton is divided into smaller pieces, and for each piece a cylindrical search radius is used to identify the mesh correspondence. The closest distance between the skeleton and corresponding mesh faces is computed at multiple points along the skeletal segment, and these are averaged to get a mean radius. Finally, all mesh correspondences of sub-branches are combined into the mesh correspondence of the branch. Concatenating the widths along the sub-branches forms a width array along the branch, with the entire branch width determined from the average of the array. This method results in one face of the original branch possibly corresponding to more than one branch's mesh correspondence, so the algorithm employs a final graph propagation step from unique mesh correspondence faces to allow branches to claim the previously conflicting faces.

The procedure described above yields a collection of disconnected non-branching skeletal segments as well as their associated mesh correspondence. The skeleton of finer-diameter processes is the initial MeshParty[7] skeleton which tracks along the mesh surface, while larger-diameter processes have skeletons that track through the center of their volume. These pieces are then all stitched together into a single connectivity graph where each non-branching segment is a node, and the edges between them represent their connectivity. Any conflicts in the mesh correspondence of adjacent nodes at stitching points is again resolved, yielding a smooth and connected mesh representation of the entire stem where each mesh face is associated with a single node (non-branching segment). This entire process is repeated for every stem submesh connected to the soma. Finally, all floating meshes outside the soma are decomposed in the same manner as the stems, and then appended to the existing skeleton if they have any faces within a threshold distance of another node (for example, in the MICrONS dataset the maximum stitch distance was set at 8 μm).

The soma(s) and decomposed stems of a neuron are then represented as a NetworkX graph object[9]. In the ideal scenario there is a single soma root node with multiple stem subgraphs, and each stem subgraph is a directed tree structure representing the connectivity between non-branching segments of the skeleton from the most proximal branch connecting to the soma to the most distal leaves of the axonal or dendritic process. In less ideal cases (which are common), cycles may exist in the skeleton due to self-touches of the axonal or dendritic process (close proximities of neurites that are incorrectly meshed together), and multiple somas may be included in a single segmented object. Handling of these cases is described in "Multi-Soma and Multi-Touch Splitting" below. The soma node contains the soma submesh and SDF values generated during the soma extraction, and each branch node in each stem stores the mesh correspondence, skeleton and width array for that node. Using these raw features, many more features of these branches can be extracted (for example spines), and additional annotations can be added (such as synapses).

Spine Detection. The non-branching segments produced by the mesh decomposition of each node present an ideal scenario for spine detection. Briefly, we started by using an existing mesh segmentation algorithms[10] which calculates a local estimate of the volume for each face of the mesh (SDF), applies a Gaussian Mixture Model (GMM) to the distribution of SDF values to enable a soft clustering of faces to k clusters, and finally minimizes an energy function defined by the alpha-expansion graph-cut algorithm to finish with a hard cluster assignment over the mesh. This last step takes a smoothness parameter controlling the likelihood that adjacent faces with concave edges will be more or less likely to be clustered together. We found that setting the number of clusters to 5 and smoothness value to 0.08 was optimal for both the MICrONS and H01 dataset to produce an over-segmentation of the branch mesh to serve as input for the next spine detection step. Then, we convert the branch segmentation into a graph representation (branch segmentation graph) where the nodes are submeshes of the segmentation and edges exist between submeshes with adjacent faces. The dendritic shaft subgraph is determined by establishing the longest contiguous shaft line path in the graph (from most likely node candidates defined by size, width and diameter thresholds), and then spine candidates are identified as subgraphs (not in the shaft path) based on size, volume, and distance from the mesh surface. The final product of this stage is a collection of individual spine submeshes with calculated spine statistics (volume, length, area). Based on these statistics, in some cases we perform an additional processing step that divides the submesh of larger spines into a spine head and spine neck. At the completion of this workflow, each mesh face in the node receives a spine label of head, neck, shaft or just "spine" (if no head and neck segmentation could be performed). Finally, the width of each branch is recomputed after removing spines that may have previously confounded that measurement. A step by step tutorial of how to optimize the parameters (and a complete explanation of each parameter available for tuning) for the spine detection and head/neck segmentation are included in the tutorials of the Github repository online.

Synapse Addition. Synapses from the reconstruction pipeline are mapped to the closest mesh face of the closest branch. Any annotations of the associated face (for example) spine head, spine neck or shaft can then be propagated to the synapse. In addition, the closest skeletal point on the associated branch object is computed to define an anchor point for the synapse on the neuron's skeleton. This anchor point enables computation of metrics such as skeletal walk distance to the closest spine, closest neurite branch point (upstream or dowstream) or skeletal walk distance to the soma.

Multi-Soma and Multi-Touch Splitting. After the initial decomposition, cycles may exist in the stem graphs due to self-touches in the decomposed mesh (regions where neurites pass very close to each other, resulting in inappropriate connectivity of faces in the mesh representation). Furthermore, stems may include edges with multiple somas if two or more somas are merged together in the same mesh object. This is a challenging problem that requires a general solution since stems can be both multi-touch and multi-soma of any degree. For example, some apical stems of neurons in the MICrONS dataset were initially connected to 9 or more somas due to close mesh proximities with apical tufts of other cells. The aim of this stage in the processing pipeline is to split the stem objects optimally, while attributing the correct portion of the stem mesh and skeleton to the correct neurite.

The workflow for splitting both multi-touches and multi-somas proceeds as follows: For every stem identified as having a multi-touch or multi-soma connection, the process first starts by identifying cyclic or soma-to-soma paths. The best edge to cut on the path is then determined using a series of heuristic rules that are applied in the order listed below:

1. Break any edge on the multi-touch or multi-soma path where the angle between the skeleton vectors of two adjacent branches on the path is larger than some threshold (reflecting the fact that neurite processes generally do not abruptly double-back on themselves).

2. Break any edge on the multi-touch or multi-soma path where more than two downstream branches exist at a branch point and the best match for skeletal branch angle and width is not on the multi-touch or multi-soma path.

3. Break any edge on the multi-touch or multi-soma path where there is a difference in width between two nodes along the path greater than a threshold amount.

After an edge is removed based on any of these rules, the process restarts and the graph representation is checked again to see if cyclic or multi-soma paths still exist. The process is repeated until no such paths exist. If no candidate edge is identified by using these rules then, depending on the user settings, the the stem may be completely discarded from the neuron object or cut at the very first or last branch.

This splitting algorithm is not guaranteed to optimally split all multi-soma or multi-touch paths, but residual errors from a sub-optimal split may be cleaned by further proofreading steps. As with any automated proofreading, the rule and relevant parameters that determined the edit are stored for subsequent evaluation and use.

Excitatory/Inhibitory Classification. Once each neuron object has a single soma, the NEURD workflow moves on to an initial round of coarse cell classification, determining whether each neuron is excitatory or inhibitory. Performing the classification at this point in the workflow enables the use of subsequent proofreading or annotation algorithms that are excitatory- or inhibitory-specific. For example, axon identification (see below) is much easier if the coarse E/I type of the cell is known beforehand. The cell class is determined via logistic regression on two features: postsynaptic shaft density (number of synapses onto dendritic shafts per μm of skeletal distance on the postsynaptic dendrite) and spine density (number of spines per μm of skeletal distance on the postsynaptic neuron). These two features enabled linearly-separable elliptical clusters for excitatory and inhibitory cells. To enable this classification prior to proofreading, we applied two restrictions to the unproofread graph that reduce potential confounds due to merge errors and ambiguity between axon and dendrite. First, we restrict to larger dendrites using a simple width threshold to exclude potential orphan axon merges, and second, we restrict to the proximal dendrite within a limited skeletal walk distance from the soma center. The latter reduces confounds due to dendritic merge errors which are more common at the distal branches (Fig. 3d). When compared to human E/I labels, the classifier results are shown in Supplemental Fig. 12a,b. These results are also robust against an approximate 10:1 and 1.8:1 excitatory to inhibitory class imbalance in the labeled MICrONS and H01 datasets respectively. Additionally, because a logistic regression is the classifier, there is a confidence score associated with the classification (which could be thresholded downstream to ensure higher fidelity labels).

Non-Neuronal Filtering. While all the neurons in the H01 dataset were hand-checked as neurons and manually annotated for cell types, the MICrONS dataset initially was not. Consequently, segments with nuclei in the MICrONS dataset could include blood vessels, glia cells, or agglomerations of orphan axons without a neuron mesh due to an incorrect nucleus merge, and we did observe this frequently in the version of MICrONS processed in this study (version 374). However this issue is now largely resolved in the most current data release with tables that indicate which segments are predicted as non-neuronal, using a method independent from NEURD, and with more accurate nuclei merging. To filter away non-neuronal segments without sacrificing a significant amount of valid neurons, we found a suitable filter using cell type classification (predicted by our logistic regression

model), number of soma synapses and the mean dendritic branch length (this may very well be a MICrONS-specific filter). Specifically, the filter excluded the following: all segments with less than 3 soma synapses, excitatory cells with less than 17 soma synapses and a mean dendritic branch length less than 35 μm, and inhibitory cells with less than 17 soma synapses and a mean dendritic branch length less than 28 μm. This filter then removed approximately 14,000 segments from all of our downstream analysis.

Axon Identification. The goal of this stage in the pipeline is to identify at most one connected component subgraph that represents the axon of the cell. In the absence of merge and split errors, identifying the axon would be a simple process of identifying the subgraph with presynaptic connections, but un-proofread datasets pose a number of challenges to this approach.

1. Due to partial reconstruction of cells, the true axon may not exist or only the axon initial segment (AIS) may exist. In this case there would be no true presynaptic connections from the cell.
2. Postsynaptic synapses on dendritic segments may be incorrectly labeled as presynaptic connections if the synapse classifier is incorrect.
3. Orphan axons may be incorrectly merged onto the cell's dendrite or soma. These frequent merge errors add incorrect presynaptic connections onto dendrites that make identifying the true axon subgraph more difficult. We observed in these volumes that if the algorithm simply chose the connected component subgraph with the highest presynaptic density, this would almost always be an orphan-axon-onto-dendrite merge error.

Our approach to axon identification is thus motivated by the following neuroscience "rules" which we implement as heuristic selection criteria. Note that in this and following sections "up" or "higher" refers to the pial direction, while "down" or "lower" refers to the white matter.

1. Axons can either project directly from the soma or from a proximal dendritic branch.
2. The axon is the only compartment (possibly excluding the soma) that forms presynaptic connections.
3. The width of axon segments are typically thinner than most dendritic branches
4. Axon segments do not have spines (although boutons may have similar features)
5. Axons receive postsynaptic inputs at the AIS, but these typically have low postsynaptic density compared to dendrites. (Note that we found the latter was not necessarily true in the H01 dataset and adjusted accordingly.)
6. For excitatory cells, the axon typically projects directly from the soma or from dendritic stems that originate from the deeper half of the soma.
7. For excitatory cells, the AIS starts at most 14 μm skeletal distance from the soma and the mean skeleton vector of the AIS typical projects downwards. The AIS does not split into multiple branches close to the soma.
8. For inhibitory cells the AIS can start much farther away (up to 80 μm skeletal distance from soma) and can come off the soma or dendritic branch. The inhibitory AIS has very low postsynaptic and presynaptic density.

NEURD identifies candidate axonal submeshes based on a combination of these heuristics applied in a cell-type-specific manner. For example, if the neuron being analyzed is excitatory, the search space of potential axonal stems is restricted to only those with a projection angle from the soma greater than 70 degrees relative to the top of the volume. Candidate AIS branches must fall within a maximum and minimum width range, they must have a synapse density below a threshold value, and they must be within a threshold skeletal distance from the soma that dictated by the cell type. If multiple potential candidates exist, the best potential axonal subgraph is selected based on longest skeletal length and closest proximity to the soma. Subgraphs that meet the heuristic criteria for axons but are not chosen as the actual axon of the cell in question retain a label of "axon-like" which facilitates subsequent proofreading.

An additional round of skeletonization is performed once the axon is detected. This re-skeletonization better captures fine details of the axonal trajectory and enables auto-proofreading methods to catch more subtle axon-to-axon merge errors.

Automatic Proofreading. The goal of the automatic proofreading stage is to identify merge errors and remove all downstream branches. NEURD implements a series of heuristic proofreading rules to identify merge errors based on graph filters - configurations of nodes and attributes that typically indicate merge errors. The graph filters are either directed one-hop or zero-hop configurations where one-hop configurations consider a node and its immediate adjacent nodes and zero-hop configurations consider only the node's features itself. These graph filters have parameters that can be tuned for axons or dendrites, excitatory or inhibitory cells, or different data sources (H01 vs MICrONS). For example, a graph filter for resolving graph configurations

of dendritic branches with 3 or more downstream nodes is useful for the H01 dataset which has a higher probability of dendritic merge errors than MICrONS. For each filter, the algorithm finds all branches that match the current error motif, and then the mesh, graph nodes and synapses associated with all those error branches are removed before proceeding to the next filter. Therefore, in the current successive order there is no overlap in error locations for different filters in the same run. Metadata for each correction is stored for subsequent review or for training non-heuristic models.

The following graph filters exist for proofreading axon submeshes. Those only used for excitatory cell types are indicated (visualizations shown in Supplemental Fig. 3).

1. High Degree Branching: The filter identifies any node (below a potential width threshold to exclude myelinated sections) with more than two downstream nodes. The filter assumes this configuration results from a single or multiple crossing axon(s) merged at an intersection point, adding 2 or more additional downstream nodes. The filter aims to identify the one true downstream node. Possible upstream to downstream node pairings are filtered away if the width, synapse density or skeletal angle differ by threshold amounts. If multiple downstream nodes are viable, the algorithm attempts to find a downstream candidate with the best match of skeletal angle or width. If no clear winner exists, the algorithm can either mark all downstream nodes as errors if the user wishes to be conservative, or can pick the best skeletal-match candidate. There are more rare scenarios where a myelinated axon has 2 collateral projections protruding very close to one another and these would be incorrectly filtered away, but a large majority of these occurances are simply merge errors.

2. Low Degree Branching Filter: The filter processes any subgraph with one upstream node and exactly two downstream nodes and is only attempted on non-myelinated axon sections (as determined by a width threshold). The method attempts to find one of the following subgraph features within this directed 3 node subgraph, and if a match occurs either the algorithm marks all of the downstream nodes as errors or attempts to determine the correct downstream pairing.

   (a) **Axon Webbing**: An error is detected by an overly-thin mesh at the branching point of an upstream to downstream node. The filter attempts to differentiate between natural branching with cell membrane that forms a "webbing like" appearance as opposed to merge errors where no such thickening occurs.

   (b) **T-Intersection**: An error is detected by the presence of downstream branches that are thicker than an upstream branch and the downstream branches are aligned and resemble a continuous non-branching axon segment.

   (c) **Double-Back** (excitatory only): An error is detected when a downstream node "doubles-back" towards the upstream node at an unnatural skeletal angle.

   (d) **Parallel Children (or Fork Divergence)**: An error is detected when the two downstream skeletons are nearly parallel without a natural gap between them.

   (e) **Synapse At Branching**: An error is detected if a synapse occurs very close to the branch point between upstream and downstream nodes; this usually indicates a merge of an orphan axon to a bouton segment.

3. Width Jump: Processes a subgraph with any number of downstream nodes (only applies to non-myelinated sections). Any downstream node with an absolute width difference between segments above a certain threshold is marked as an error.

The following graph filters exist for proofreading dendrite submeshes.

1. Axon on Dendrite: Nodes that were previously labeled “axon-like” during the process of axon identification (see above) that do not end up in the axon submesh are marked as errors.

2. High Degree Branching (H01 only, excitatory only, apical trunks excluded): An error is detected using the same algorithm as described above for the axonal High Degree Branching Filter except it is applied to dendritic nodes below a thresholded width.

3. Width Jump: An error is detected using the same graph filter as described for axons but with larger width difference thresholds

4. Double Back: An error is detected using the same algorithm and parameters as for axons above.

APL Validation of Multi-Soma Splitting and High Confidence Orphan Merge Edits. Our collaborators at APL (Johns Hopkins University Applied Physics Laboratory) helped extensively validate multiple aspects of the NEURD automated proofreading workflow. In particular, they provided information about the following:

1. Validation of specific edits in the context of multi-soma splitting.

2. Data about the time that proofreaders took to evaluate these split suggestions compared to other methods.

3. Validation of specific edits focused on axon-onto-dendrite or axon-onto-axon merges

This information made it possible to determine whether our suggestions for multi-soma split locations speed up the process (they do, more than three-fold), and if we could identify a set of heuristics and parameters for axon-on-dendrite and axon-on-axon merge corrections that could be executed with high confidence on the entire volume without human intervention (we identified two classes of edits with performance >95%, and more than 150,000 of them have been applied to the MICrONS volume to date; see Supplemental Fig. 4).

A key method for both validating and applying automatic edits was the functionality in Neuroglancer[11] which allows the placement of point annotations to define a split in the PyChunkedGraph segmentation[12,13]. To facilitate the proofreading process, APL created a web-based interface called NeuVue[14] that allows for the efficient queuing, review and execution of split suggestions in Neuroglancer. We built the logic required to translate mesh errors identified by NEURD into split point annotations that can be executed by the NeuVue pipeline. This capability allowed proofreaders at APL to not only evaluate error locations identified by NEURD, but also a proposed set of points that could be subsequently executed in the PyChunkedGraph to correct the error. For a more detailed description of the NeuVue review pipeline see[14].

For multi-soma split edits, we generated point annotations for suggested splits that would contribute to separating neurons with between 2 and 6 possible somas in a single segment. APL had both experts and trained student proofreaders review these edits. The classifications for each of the edits was one of the following:

1. “yes”: same split point annotations the proofreader would have chosen.

2. “yesConditional”: split point is correct, but point annotations required very minor adjustment.

3. “errorNearby”: split point is not correct but is very close by, and split point annotations require adjustment

4. “no”: the correct split location was not at or near the suggested location

The expert proofreaders reviewed 5134 unique suggestions with no overlap between proofreaders, while the student proofreaders reviewed 2355 suggestions with some redundancy so the same suggested edit was seen by multiple student proofreaders and a majority vote determined the classification of the edit. The results of reviewing the first approximately 4000 of those edits are shown in Supplemental Fig. 4a and the accuracy was determined to be 76.12% when the “yes”,”yesConditional” and “errorNearby” categories were considered true positive classes. Additionally, because each split suggestion had an associated heuristic rule and set of parameters that was used to generate the suggestion, we were able to show that some rules were much higher fidelity than others, and that the parameters could be tuned to achieve a higher classification accuracy (Supplemental Fig. 4b,c).

To compare against the performance achieved with NEURD suggestions, multi-soma splits were also performed by expert proofreaders using a tool that highlighted the path along the neural processes connecting two somas. This comparison enabled us to measure if the NEURD suggestions could potentially speed up the soma-splitting process. Because a single segment with a multi-soma merge could contain more than 2 somas and because different merges may require a different amount of work and number of cuts to be applied, we measured the overall time spent reviewing all edits and estimated the additional time that would have been required to make the slight adjustments required in the case of "yesConditional" (+30s) or "errorNearby" (+60s), prior to executing the edit. Note that “no” classifications added time to the review process without any possibility of contributing to an actual edit. Based on these metrics, we divided total time by total number of edits to determine the mean time per edit. We then compared this metric to the mean time per edit when proofreaders used a standard pathfinding tool that displayed the skeletal path connecting multiple somas, and they had to search along this path to identify errors manually. We observed a more than three-fold speed up when using NEURD suggestions (Supplemental Fig. 4d).

Finally, outside the context of multi-soma splitting, APL proofreaders evaluated two kinds of merge error corrections that strip orphan axons from both excitatory and inhibitory neurons: axon-on-dendrite, and high degree axon-on-axon. The feedback on each error from proofreaders using the NeuVue pipeline allowed us to determine a subset of parameters that was correlated with high accuracy. Thresholds for the axon-on-dendrite included minimum parent width, distance from the soma, and skeletal length of the error segment. Thresholds for axon-on-axon included a minimum skeletal length, and a branching pattern that resembled a two line segment crossing, where the segments are closer to perpendicular in order to make the correct connectivity more obvious. For the review of orphan merge errors, an additional label was included in the true positive class: "yesPartial", which indicated that part but not all of the merge was removed by the split point annotations. The feedback from this effort provided our collaboration with enough evidence to then apply nearly 150,000 of these high-confidence automatic edits back into the current dynamic segmentation of the MICrONS dataset (Supplemental Fig. 4e).

Automatic Compartment Labeling. After automatic proofreading removes as many merge errors as possible, compartment labeling is performed for excitatory cells, classifying graph submeshes as apical trunk, apical tuft, basal, and oblique. NEURD first attempts to identify the apical trunk based on the geometry relative to the soma and total skeletal length. Branches downstream of the end of the apical trunk are classified as the apical tuft, and branches off of the trunk with a skeletal angle

close to 90 degrees are labeled as oblique. If the criteria for a defined trunk is not met, then NEURD applies a generic "apical" label. Other dendrites are classified as basal. Additionally, for the MICrONS dataset if the soma center is close enough to the pia as defined by a depth threshold, there can be multiple generic "apical" stems protruding from the top of the soma if they each meet the required width and geometry thresholds.

Connectome-level features computed by NEURD. At the level of the connectome graph, nodes represent individual single-nucleus neurons and edges represent synaptic connections. In addition to the rich sub-cellular features that NEURD computes for the decomposition graph of each cell, NEURD provides a variety of features at the connectome graph level:

1. Node Attributes: a wide range of global properties measured for the individual cells (compartment skeletal lengths, synapses, bounding box, spine densities, synapse densities, average width, cell type, etc).

2. Edges: connections between neurons with a valid presynaptic connection and postsynaptic connection where neither were filtered away in the auto-proofreading stage

3. Edge Attributes: properties for each of the presynaptic and postsynaptic neurons (compartment, skeletal/euclidean distance to neuron's soma, size, spine label) and properties of the entire synaptic connection between neurons (euclidean/skeletal distance from soma of presynaptic neuron to soma of postsynaptic neuron, etc).

GNN Cell Typing. Using PyTorch geometric software[15] we implemented a Graph Neural Network architecture to build a supervised cell type classifier (including subclasses of excitatory and inhibitory cells) from dendritic graph structure in the NEURD decompositions. We trained this classifier using manual cell types from the Allen Institute for Brain Science[16]. To create an input graph for the classifier we first removed the soma node and filtered away the axonal subgraph and any dendritic stems with less than 25 μm of total skeletal length. Each node was annotated with the following feature set:

1. Skeleton features, where theta and phi refer to polar coordinates of the skeleton vector in 3-D (skeleton_length, skeleton_vector_upstream_theta, skeleton_vector_upstream_phi, skeleton_vector_downstream_theta, skeleton_vector_downstream_phi)

2. Width features (width, width_upstream, width_downstream synapse)

3. Spine features (n_spines, spine_volume_sum, n_synapses_post, n_synapses_pre, n_synapses_head_postsyn, n_synapses_neck_postsyn, n_synapses_shaft_postsyn, n_synapses_no_head_postsyn, synapse_volume_shaft_postsyn_sum, synapse_volume_head_postsyn_sum, synapse_volume_no_head_postsyn_sum, synapse_volume_neck_postsyn_sum, synapse_volume_postsyn_sum)

Note: for all the synapse_volume features the synapses were not scaled to nm^3 and instead were left in $voxels^3$. Therefore, for the MiCrONS dataset if the synapse volumes were already converted to nm^3 scale, then these synapse volume would need to be divided by 640 (the product of the 4x4x40 voxel to nm scaling).

For classifiers, the soma volume and number of soma synapses for the neuron are added to each node's feature vector and also the starting stem angle (2-D angle between the vector from the soma center to the stem's root skeleton point and the vector in the direction of the pia) is added to each node in every stem. For the stem-based classifier, classification is performed on each stem individually.

The GNN architecture used a 2 layer Graph Convolutional Network (128 hidden units for each layer, ReLU activation function) followed with one linear layer. The aggregation and update steps were implemented using self loops and symmetric normalization as shown here:

$$\mathbf{h}_u^{(k)} = \sigma\left(\mathbf{W}^{(k)} \sum_{v \in \mathcal{N}(u) \cup \{u\}} \frac{\mathbf{h}_v}{\sqrt{|\mathcal{N}(u)||\mathcal{N}(v)|}}\right)$$

where $\mathbf{h}_u^{(k)}$ is the embedding for node u at layer k, $\mathcal{N}(u)$ are the neighbors for node u, $\mathbf{W}^{(k)}$ is the learned weight matrix at layer k and σ is the chosen non-linearity. For graph pooling (to get one learned vector for each graph), the weighted average of all nodes after the final hidden layer was taken (weighted by the skeletal length of the node). Additionally, for the stem based classifier a max pooling vector was concatenated to the weighted average vector output. A 60%, 20%, 20% split for training, validation and test sets was used for labeled datasets of n = 873 whole neurons and n = 4,114 stems (Supplemental Fig. 14)

A full tutorial of how to load and perform inference runs on the pretrained full neuron and limb based GNN models is included on the github repository.

Proximities. Identifying axon-dendrite proximities makes it possible to determine how often a pair of neurons capitalizes on an opportunity to form a synaptic connection. Proximities are regions where the axon of one neuron passes within a few microns of the dendrite of another neuron. They can be annotated with the same features (dendritic compartment, neural subtype) as synapses, regardless of whether a connection was formed (Fig. 5a). Proximities are identified for all neuron pairs in the volume. To reduce the number of pairwise computations, NEURD first checks whether the bounding box of the presynaptic axon skeleton and postsynaptic dendrite skeleton have any overlap. In order to reduce computation time in the the MICrONS dataset, presynaptic neurons are further restricted to those with at least five axonal synapses, and in the MICrONS volume postsynaptic neurons are restricted to neurons with at least 1 mm of dendritic length (this latter restriction excludes approximately 1% of all MICrONS neurons).

The proximity calculation is performed by converting the axonal skeleton of the presynaptic neuron and the postsynaptic skeleton to an array of coordinates without edges (at one μm skeletal walk resolution). A local width and compartment label is associated with every point, and the soma of the postsynaptic neuron is converted to a uniform sampling of the surface mesh face centers for its skeletal representation. The cleaned synaptic connections between the pre and post neuron are retrieved (if there were any) and then the main proximity loop begins.

1. The closest distance between a presynaptic coordinate and postsynaptic coordinate is computed (where the distances can be adjusted for postsynaptic width by subtracting the local width from the euclidean distance). This minimum distance is the current proximity distance. If the current proximity distance exceeds the thresholded maximum proximity distance (set at 5 μm for both the MICrONS and H01 dataset), then the loop is exited and no more proximities are computed, but otherwise the workflow proceeds.

2. The following metrics are computed or collected for each proximity: The presynaptic and postsynaptic coordinate of the minimum distance pair, the distance between these coordinates (the proximity distance), the compartment labels and width at the postsynaptic coordinate, the presynaptic and postsynaptic skeletal walk distance, the number of spines and synaptic connections within a three μm radius of the presynaptic and postsynaptic coordinates.

3. After these features are collected, the skeleton points within a set radius (10 μm) of the presynaptic proximity coordinate are filtered away from the array of axon presynaptic coordinates.

4. All proximity information is saved and the loop continues until the current proximity distance exceeds the threshold.

Functional Connectomics. We considered pairs of synaptically-connected functionally-matched cells available in the MICrONS dataset, restricting to pairs where both neurons met a minimal set of functional quality criteria (test score greater than 0.2 and an oracle score (correlation of a neurons response to the leave-one-out mean response across a repeated image stimuli) greater than 0.3, see Ding et al. [17], Wang and Tolias [18]). Synaptic connections were discarded if they were not onto postsynaptic spines (to help guard against possible inhibitory merge errors resulting in increased connectivity between neurons). We then divided the pairs into groups based on whether they had 1, 2, 3 or 4+ synapses between them. The final number of functionally matched pyramidal pairs available from automatic proofreading alone were as follows: 1 synapse (5350), 2 synapses (280), 3 synapses (34) and 4+ synapses (11). We then investigated how the mean functional response correlation varies as a function of the four different multi-synaptic groups. The response correlation was calculated as detailed in [17,18] through the *in silico* response correlation of their model.

Analysis Subsets and Distributions. All morphological, connectomic and functional connectomic analyses excluded the manually proofread neurons used for validating NEURD proofreading, a small fraction of cells that errored out during the NEURD preprocessing pipeline due to a variety of factors (corrupted mesh, manifold and watertight properties unable to be automatically fixed, etc.), and non-neuronal cells that were filtered away as described in the "Non-Neuronal Filtering" section. Additionally, we excluded some neurons with very incomplete reconstructions from some analyses. For example, only a subset of automatically proofread neurons with an axon length longer than 50 μm were used in the investigation of questions concerning synapses onto the axon initial segment, in order to not skew results due to a reconstruction bias. Analyses of connectivity, etc between different cell subtypes only used neurons with high confidence labels from the GNN classifier (softmax output >0.7). Complete Ns for all analyses and different stages of the pipeline are included in the Supplemental Table 2.

Software

Python was the primary software used for this work. For automatic segmentation and deconvolution of calcium imaging data we used CAIMAN. For mesh processing we used Meshparty, Trimesh, Meshlab (and Meshlabserver) and CGAL (CGAL and MeshLab usage required custom python wrappers). For graphical visualizations we used Neuroglancer, Ipyvolume, Matplotlib and Seaborn. For code development and deployment we used Jupyter, Docker, and Kubernetes. For basic graph representations

and querying we used Networkx, Dotmotif, and NetSci. For storing and managing data we used DataJoint and CAVE. Our custom built analysis pipeline also used general tools for various tasks (like model training, statistical analysis, etc.) such as Numpy, Pandas, SciPy, Scikit-learn, PyTorch.

Bibliography

[88] Dimitri Yatsenko, Jacob Reimer, Alexander S. Ecker, Edgar Y. Walker, Fabian Sinz, Philipp Berens, Andreas Hoenselaar, Ronald James Cotton, Athanassios S. Siapas, and Andreas S. Tolias. DataJoint: managing big scientific data using MATLAB or Python. *bioRxiv*, page 031658, November 2015. doi: 10.1101/031658.

[89] Dimitri Yatsenko, Edgar Y. Walker, and Andreas S. Tolias. DataJoint: A Simpler Relational Data Model. *arXiv:1807.11104 [cs]*, July 2018. arXiv: 1807.11104.

[90] Paolo Cignoni, Massimiliano Corsini, and Guido Ranzuglia. MeshLab: an Open-Source 3D Mesh Processing System. *ERCIM News*, 2008(73), 2008.

[91] MICrONS Consortium, J. Alexander Bae, Mahaly Baptiste, Agnes L. Bodor, Derrick Brittain, JoAnn Buchanan, Daniel J. Bumbarger, Manuel A. Castro, Brendan Celii, Erick Cobos, Forrest Collman, Nuno Maçarico da Costa, Sven Dorkenwald, Leila Elabbady, Paul G. Fahey, Tim Fliss, Emmanouil Froudarakis, Jay Gager, Clare Gamlin, Akhilesh Halageri, James Hebditch, Zhen Jia, Chris Jordan, Daniel Kapner, Nico Kemnitz, Sam Kinn, Selden Koolman, Kai Kuehner, Kisuk Lee, Kai Li, Ran Lu, Thomas Macrina, Gayathri Mahalingam, Sarah McReynolds, Elanine Miranda, Eric Mitchell, Shanka Subhra Mondal, Merlin Moore, Shang Mu, Taliah Muhammad, Barak Nehoran, Oluwaseun Ogedengbe, Christos Papadopoulos, Stelios Papadopoulos, Saumil Patel, Xaq Pitkow, Sergiy Popovych, Anthony Ramos, R. Clay Reid, Jacob Reimer, Casey M. Schneider-Mizell, H. Sebastian Seung, Ben Silverman, William Silversmith, Amy Sterling, Fabian H. Sinz, Cameron L. Smith, Shelby Suckow, Marc Takeno, Zheng H. Tan, Andreas S. Tolias, Russel Torres, Nicholas L. Turner, Edgar Y. Walker, Tianyu Wang, Grace Williams, Sarah Williams, Kyle Willie, Ryan Willie, William Wong, Jingpeng Wu, Chris Xu, Runzhe Yang, Dimitri Yatsenko, Fei Ye, Wenjing Yin, and Szi-chieh Yu. Functional connectomics spanning multiple areas of mouse visual cortex, August 2021. Pages: 2021.07.28.454025 Section: New Results.

[92] Alexander Shapson-Coe, Michał Januszewski, Daniel R. Berger, Art Pope, Yuelong Wu, Tim Blakely, Richard L. Schalek, Peter Li, Shuohong Wang, Jeremy Maitin-Shepard, Neha Karlupia, Sven Dorkenwald, Evelina Sjostedt, Laramie Leavitt, Dongil Lee, Luke Bailey, Angerica Fitzmaurice, Rohin Kar, Benjamin Field, Hank Wu, Julian Wagner-Carena, David Aley, Joanna Lau, Zudi Lin, Donglai Wei, Hanspeter Pfister, Adi Peleg, Viren Jain, and Jeff W. Lichtman. A connectomic study of a petascale fragment of human cerebral cortex. Technical report, bioRxiv, May 2021.

[93] Ilker O. Yaz and Sébastien Loriot. Triangulated Surface Mesh Segmentation. In *CGAL User and Reference Manual*. CGAL Editorial Board, 5.5.2 edition, 2023.

[94] Sven Dorkenwald. sdorkenw/MeshParty, March 2022. original-date: 2018-09-11T23:38:46Z.

[95] Xiang Gao, Sébastien Loriot, and Andrea Tagliasacchi. Triangulated Surface Mesh Skeletonization. In *CGAL User and Reference Manual*. CGAL Editorial Board, 5.5.2 edition, 2023.

[96] Aric A. Hagberg, Daniel A. Schult, and Pieter J. Swart. Exploring Network Structure, Dynamics, and Function using NetworkX. In Gaël Varoquaux, Travis Vaught, and Jarrod Millman, editors, *Proceedings of the 7th Python in Science Conference*, pages 11 – 15, Pasadena, CA USA, 2008.

[97] Andreas Fabri and Laurent Rineau. CGAL and the Qt Graphics View Framework. In *CGAL User and Reference Manual*. CGAL Editorial Board, 5.5.2 edition, 2023.

[98] Eric Perlman. Visualizing and Interacting with Large Imaging Data. *Microscopy and Microanalysis*, 25(S2):1374–1375, August 2019. ISSN 1431-9276, 1435-8115. doi: 10.1017/S1431927619007608. Publisher: Cambridge University Press.

[99] Sven Dorkenwald, Nicholas L Turner, Thomas Macrina, Kisuk Lee, Ran Lu, Jingpeng Wu, Agnes L Bodor, Adam A Bleckert, Derrick Brittain, Nico Kemnitz, William M Silversmith, Dodam Ih, Jonathan Zung, Aleksandar Zlateski, Ignacio Tartavull, Szi-Chieh Yu, Sergiy Popovych, William Wong, Manuel Castro, Chris S Jordan, Alyssa M Wilson, Emmanouil Froudarakis, JoAnn Buchanan, Marc M Takeno, Russel Torres, Gayathri Mahalingam, Forrest Collman, Casey M Schneider-Mizell, Daniel J Bumbarger, Yang Li, Lynne Becker, Shelby Suckow, Jacob Reimer, Andreas S Tolias, Nuno Macarico da Costa, R Clay Reid, and H Sebastian Seung. Binary and analog variation of synapses between cortical pyramidal neurons. *eLife*, 11:e76120, November 2022. ISSN 2050-084X. doi: 10.7554/eLife.76120. Publisher: eLife Sciences Publications, Ltd.

[100] Sven Dorkenwald, Claire E. McKellar, Thomas Macrina, Nico Kemnitz, Kisuk Lee, Ran Lu, Jingpeng Wu, Sergiy Popovych, Eric Mitchell, Barak Nehoran, Zhen Jia, J. Alexander Bae, Shang Mu, Dodam Ih, Manuel Castro, Oluwaseun Ogedengbe, Akhilesh Halageri, Kai Kuehner, Amy R. Sterling, Zoe Ashwood, Jonathan Zung, Derrick Brittain, Forrest Collman, Casey Schneider-Mizell, Chris Jordan, William Silversmith, Christa Baker, David Deutsch, Lucas Encarnacion-Rivera, Sandeep Kumar, Austin Burke, Doug Bland, Jay Gager, James Hebditch, Selden Koolman, Merlin Moore, Sarah Morejohn, Ben Silverman, Kyle Willie, Ryan Willie, Szi-chieh Yu, Mala Murthy, and H. Sebastian Seung. FlyWire: online community for whole-brain connectomics. *Nature Methods*, 19(1): 119–128, January 2022. ISSN 1548-7105. doi: 10.1038/s41592-021-01330-0. Number: 1 Publisher: Nature Publishing Group.

[101] Daniel Xenes, Lindsey M. Kitchell, Patricia K. Rivlin, Rachel Brodsky, Hannah Gooden, Justin Joyce, Diego Luna, Raphael Norman-Tenazas, Devin Ramsden, Kevin Romero, Victoria Rose, Marisel Villafane-Delgado, William Gray-Roncal, and Brock Wester. NeuVue: A Framework and Workflows for High-Throughput Electron Microscopy Connectomics Proofreading, July 2022. Pages: 2022.07.18.500521 Section: New Results.

[102] Matthias Fey and Jan Eric Lenssen. Fast Graph Representation Learning with PyTorch Geometric, May 2019. original-date: 2017-10-06T16:03:03Z.

[103] Casey M. Schneider-Mizell, Agnes Bodor, Derrick Brittain, JoAnn Buchanan, Daniel J. Bumbarger, Leila Elabbady, Daniel Kapner, Sam Kinn, Gayathri Mahalingam, Sharmishtaa Seshamani, Shelby Suckow, Marc Takeno, Russel Torres, Wenjing Yin, Sven Dorkenwald, J. Alexander Bae, Manuel A. Castro, Paul G. Fahey, Emmanouil Froudakis, Akhilesh Halageri, Zhen Jia, Chris Jordan, Nico Kemnitz, Kisuk Lee, Kai Li, Ran Lu, Thomas Macrina, Eric Mitchell, Shanka Subhra Mondal, Shang Mu, Barak Nehoran, Stelios Papadopoulos, Saumil Patel, Xaq Pitkow, Sergiy Popovych, William Silversmith, Fabian H. Sinz, Nicholas L. Turner, William Wong, Jingpeng Wu, Szi-chieh Yu, MICrONS Consortium, Jacob Reimer, Andreas S. Tolias, H. Sebastian Seung, R. Clay Reid, Forrest Collman, and Nuno Maçarico da Costa. Cell-type-specific inhibitory circuitry from a connectomic census of mouse visual cortex, February 2023. Pages: 2023.01.23.525290 Section: New Results.

[104] Zhuokun Ding, Paul Fahey, Stelios Papadopoulos, Eric Wang, Alexander Kunnin, Brendan Celli, Christos Papadopoulos, Andersen Chang, Jiakun Fu, Zhiwei Ding, Saumil Patel, MICrONS Consortium, Emmanouil Froudarakis, Sebastian Seung, Forrest Collman, Nuno Ma\textbackslashc{c}arico da Costa, Clay Reid, Edgar Walker, Xaq Pitkow, Jacob Reimer, and Andreas Tolias. Functional connectomics reveals general wiring rule in mouse visual cortex, March 2023.

[105] Eric Wang and Andreas Tolias. Foundation model of the cortex, March 2023.

[106] R. Azouz, C. M. Gray, L. G. Nowak, and D. A. McCormick. Physiological properties of inhibitory interneurons in cat striate cortex. *Cerebral Cortex (New York, N.Y.: 1991)*, 7(6):534–545, September 1997. ISSN 1047-3211. doi: 10.1093/cercor/7.6.534.

[107] Yasuo Kawaguchi, Fuyuki Karube, and Yoshiyuki Kubota. Dendritic branch typing and spine expression patterns in cortical nonpyramidal cells. *Cerebral Cortex (New York, N.Y.: 1991)*, 16(5): 696–711, May 2006. ISSN 1047-3211. doi: 10.1093/cercor/bhj015.

[108] Leila Elabbady, Sharmishtaa Seshamani, Shang Mu, Gayathri Mahalingam, Casey Schneider-Mizell, Agnes Bodor, J. Alexander Bae, Derrick Brittain, JoAnn Buchanan, Daniel J. Bumbarger, Manuel A. Castro, Erick Cobos, Sven Dorkenwald, Paul G. Fahey, Emmanouil Froudarakis, Akhilesh Halageri, Zhen Jia, Chris Jordan, Dan Kapner, Nico Kemnitz, Sam Kinn, Kisuk Lee, Kai Li, Ran Lu, Thomas Macrina, Eric Mitchell, Shanka Subhra Mondal, Barak Nehoran, Stelios Papadopoulos, Saumil Patel, Xaq Pitkow, Sergiy Popovych, Jacob Reimer, William Silversmith, Fabian H. Sinz, Marc Takeno, Russel Torres, Nicholas Turner, William Wong, Jingpeng Wu, Wenjing Yin, Szi-chieh Yu, Andreas Tolias, H. Sebastian Seung, R. Clay Reid, Nuno Maçarico Da Costa, and Forrest Collman. Quantitative Census of Local Somatic Features in Mouse Visual Cortex, July 2022. Pages: 2022.07.20.499976 Section: New Results.

[109] Casey M Schneider-Mizell, Agnes L Bodor, Forrest Collman, Derrick Brittain, Adam Bleckert, Sven Dorkenwald, Nicholas L Turner, Thomas Macrina, Kisuk Lee, Ran Lu, Jingpeng Wu, Jun Zhuang, Anirban Nandi, Brian Hu, JoAnn Buchanan, Marc M Takeno, Russel Torres, Gayathri Mahalingam, Daniel J Bumbarger, Yang Li, Thomas Chartrand, Nico Kemnitz, William M Silversmith, Dodam Ih, Jonathan Zung, Aleksandar Zlateski, Ignacio Tartavull, Sergiy Popovych, William Wong, Manuel Castro, Chris S Jordan, Emmanouil Froudarakis, Lynne Becker, Shelby Suckow, Jacob Reimer, Andreas S Tolias, Costas A Anastassiou, H Sebastian Seung, R Clay Reid, and Nuno Maçarico da Costa. Structure and function of axo-axonic inhibition. *eLife*, 10:e73783, December 2021. ISSN 2050-084X. doi: 10.7554/eLife.73783. Publisher: eLife Sciences Publications, Ltd.

[110] Gregg A. Wildenberg, Matt R. Rosen, Jack Lundell, Dawn Paukner, David J. Freedman, and Narayanan Kasthuri. Primate neuronal connections are sparse in cortex as compared to mouse. *Cell Reports*, 36(11):109709, September 2021. ISSN 2211-1247. doi: 10.1016/j.celrep.2021.109709.

[111] Sharmishtaa Seshamani, Leila Elabbady, Casey Schneider-Mizell, Gayathri Mahalingam, Sven Dorkenwald, Agnes Bodor, Thomas Macrina, Daniel Bumbarger, JoAnn Buchanan, Marc Takeno, Wenjing Yin, Derrick Brittain, Russel Torres, Daniel Kapner, Kisuk lee, Ran Lu, Jinpeng Wu, Nuno daCosta, Clay Reid, and Forrest Collman. Automated Neuron Shape Analysis from Electron Microscopy, May 2020. arXiv:2006.00100 [cs].

[112] K. M. Harris and J. K. Stevens. Dendritic spines of CA 1 pyramidal cells in the rat hippocampus: serial electron microscopy with reference to their biophysical characteristics. *The Journal of Neuroscience: The Official Journal of the Society for Neuroscience*, 9(8):2982–2997, August 1989. ISSN 0270-6474. doi: 10.1523/JNEUROSCI.09-08-02982.1989.

[113] Jon I. Arellano, Ruth Benavides-Piccione, Javier DeFelipe, and Rafael Yuste. Ultrastructure of Dendritic Spines: Correlation Between Synaptic and Spine Morphologies. *Frontiers in Neuroscience*, 1(1):131–143, October 2007. ISSN 1662-4548. doi: 10.3389/neuro.01.1.1.010.2007.

[114] Petra Wahle, Eric Sobierajski, Ina Gasterstädt, Nadja Lehmann, Susanna Weber, Joachim HR Lübke, Maren Engelhardt, Claudia Distler, and Gundela Meyer. Neocortical pyramidal neurons with axons emerging from dendrites are frequent in non-primates, but rare in monkey and human. *eLife*, 11:e76101, April 2022. ISSN 2050-084X. doi: 10.7554/eLife.76101. Publisher: eLife Sciences Publications, Ltd.

[115] Katherine L. Villa and Elly Nedivi. Excitatory and Inhibitory Synaptic Placement and Functional Implications. In Kazuo Emoto, Rachel Wong, Eric Huang, and Casper Hoogenraad, editors, *Dendrites*, pages 467–487. Springer Japan, Tokyo, 2016. ISBN 978-4-431-56048-7 978-4-431-56050-0. doi: 10.1007/978-4-431-56050-0_18.

[116] Yang-Sun Hwang, Catherine Maclachlan, Jérôme Blanc, Anaëlle Dubois, Carl C. H. Petersen, Graham Knott, and Seung-Hee Lee. 3D Ultrastructure of Synaptic Inputs to Distinct GABAergic Neurons in the Mouse Primary Visual Cortex. *Cerebral Cortex (New York, N.Y.: 1991)*, 31(5):2610–2624, March 2021. ISSN 1460-2199. doi: 10.1093/cercor/bhaa378.

[117] M Megıas, Zs Emri, T. F Freund, and A. I Gulyás. Total number and distribution of inhibitory and excitatory synapses on hippocampal CA1 pyramidal cells. *Neuroscience*, 102(3):527–540, February 2001. ISSN 0306-4522. doi: 10.1016/S0306-4522(00)00496-6.

Supplemental Figures

a MICrONS Mesh Processing Validation

category	precision	recall	f1	# datapoints
soma	1.000	1.000	1.000	195
axon	0.995	0.968	0.981	192
glia	1.000	1.000	1.000	188
spine	0.896	0.870	0.883	22226
spine (> 0.7 um)	0.942	0.978	0.960	18030

b H01 Mesh Processing Validation

category	precision	recall	f1	# datapoints
soma	0.990	0.990	0.990	102
glia	1.000	1.000	1.000	100
axon	0.942	0.875	0.907	76
spine	0.831	0.765	0.796	7945
spine (> 0.7 um)	0.892	0.953	0.922	5851

Supplemental Fig. 1. Mesh Processing Pipeline Validation a (MICrONS) Validation scores of automatic submesh (compartment) identification in comparison to human labels. This dataset was produced by randomly presenting mesh segments to human proofreaders who evaluated automatic annotations as true positive, true negative, false positive, or false negative (TP,TN,FP,or FN) for each structure. Only glia merges larger than the volume of a 5 μm radius sphere were considered glia merges in this processing step. The "spine (> 0.7 μm)" row reports the agreement between the automatic spine detection and human spine labeling for spines with a skeletal length greater than 0.7 μm. Below this threshold there was disagreement even among human proofreaders about whether small protrusions should be classified as a spine or not. Precision = true positives / (true positives + false positives), Recall = true positives / (true positives + false negatives), F1 = (precision * recall)/(precision + recall). The F1 statistic serves as a weighting between the recall and precision to ensure a degenerative solution is not achieved (Ex: marking all samples as positives in order to maximize recall at the detriment of precision). **b** Identical validation scores for H01 dataset.

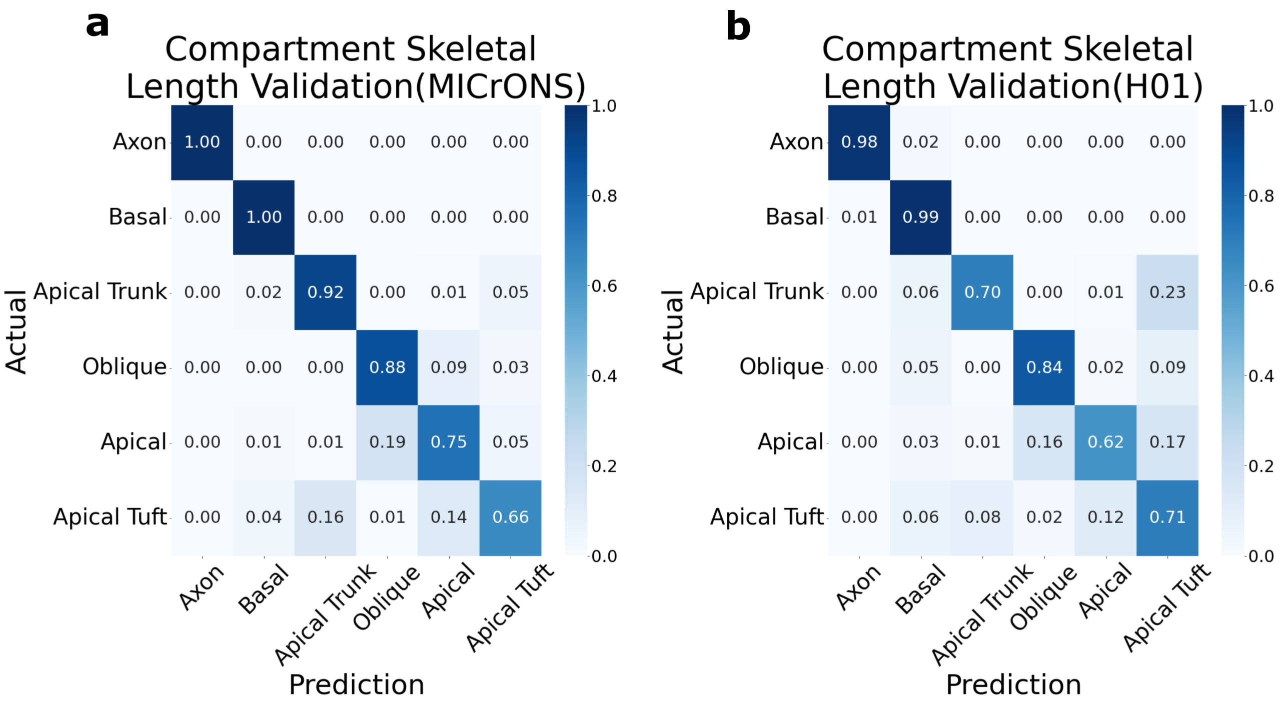

Supplemental Fig. 2. Automatic Submesh (Compartment) Labeling Validation a (MICrONS) Confusion matrix comparing automatic submesh labeling to human labels (random sampling of 158 processed cells). Here the TP,FP,TN,FN metrics are computed using skeletal length agreement for each compartment. Therefore, cells with longer stems or cells with more stems of a certain compartment type more heavily influenced the scores due to the skeletal length weighting. **b** (H01) Confusion Matrix comparing automatic submesh labeling to human labeled submeshes (random sampling of 89 processed cells). Across both datasets, compartment labeling was nearly perfect for separating the axon, basal, and apical supercategory (the union of oblique, apical, apical tuft and apical trunk) compartments, but was less consistent for separating the sub-compartments of the apical supercategory.

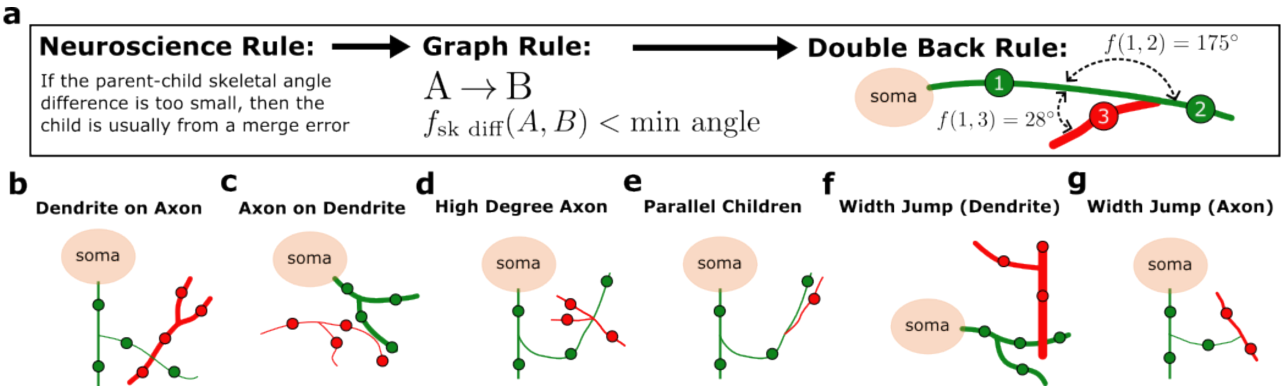

Supplemental Fig. 3. Automatic Proofreading Rule Visualizations a Example implementation of domain knowledge as a subgraph rule to automatically identify and remove merge errors. Most of the same rules can be applied across excitatory and inhibitory cells in the MICrONS and H01 volume as-is, or with small changes in parameters. **b-g** Visual representation of other subgraph rules implemented for the automatic proofreading stage as described in the "Methods-Automatic Proofreading" section.

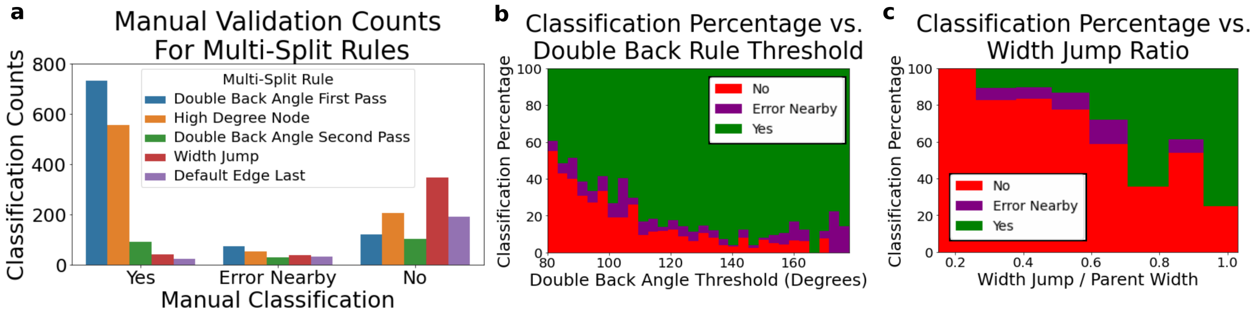

d

Time Per Edit During Multi-Soma Splitting
Auto Proof vs. Soma to Soma Path Guided

Method	Time (s)	# of Edits	Time per Edit (s/edit)	Speed Ratio vs No Auto Proof
Soma to Soma Path Guided	10285126	19035	540.33	1.00
Auto Proof Guided	816796	5736	142.40	3.79

e

High Confidence Merge Edits Validation

Merge Type	Accuracy (Excitatory)	# of Datapoints (Excitatory)	Accuracy (Inhibitory)	# of Datapoints (Inhibitory)
High Degree Axon	0.99	201	0.99	296
Axon on Dendrite	0.94	869	0.96	107

Supplemental Fig. 4. Proofreading Validation All validation was performed by the proofreading team at Johns Hopkins University Applied Physics Laboratory (APL). In an initial round of validation, suggested error locations were evaluated in the context of splitting multi-soma cells in the MICrONS volume. As a result we were able to measure both the accuracy of these proofreading rules and the speed benefits of a semi-supervised approach compared to fully-manual proofreading. Additionally, the accuracy of two automatic proofreading rules with high-confidence parameters (axon on dendrite, high degree axon on axon merges) were evaluated. **a** Validation of split locations predicted by automatic multi-split algorithm. "Yes" (indicates that the proposed split can be executed immediately), "Error Nearby" indicates that the split location is correct within 20 μm, but that the human proofreader slightly modified the suggested split points (this threshold was determined so that every suggestion in this category would prove useful to and increase the speed of human proofreaders after their attention is drawn to the relevant location), and "No" indicates that the true split location was far from the predicted location or no merge error was detected by the human proofreader). The heuristic splitting rules are applied in the order indicated by the legend. The automated proofreading accuracy varied substantially over the different heuristic rules with an overall accuracy of 76.12 % when "Yes" and "Error Nearby" are considered true positives. The best-performing rules can be selected for different datasets. **b** Even for a single rule, thresholds can be tuned to optimize performance. Manual classification of split locations predicted by the "Double Back" rule as a function of the angle measured at each predicted location illustrates that a higher accuracy could be achieved by setting a higher threshold for this algorithm. **c** Manual classification of split locations predicted by the width jump rule as a function of the width jump at each predicted location, illustrating another example where interpretable thresholds can be adjusted for higher precision. **d** Elapsed time statistics as humans performed manual tasks of splitting multi-soma neurons either using a tool that showed the path along the mesh between two somas or using the suggested split locations from our automatic multi-split algorithm. The speed at which humans could apply cuts in the correct locations more than tripled when using suggestions provided by the NEURD multi-split algorithm. Note: the validation is measured as the average amount of time for a single edit in the multi-soma splitting process; a single multi-soma split might require 20 or more edits to completely resolve the merge. **e** Accuracy of two automatic proofreading rules with high-confidence parameters (high degree axon on axon, axon on dendrite).

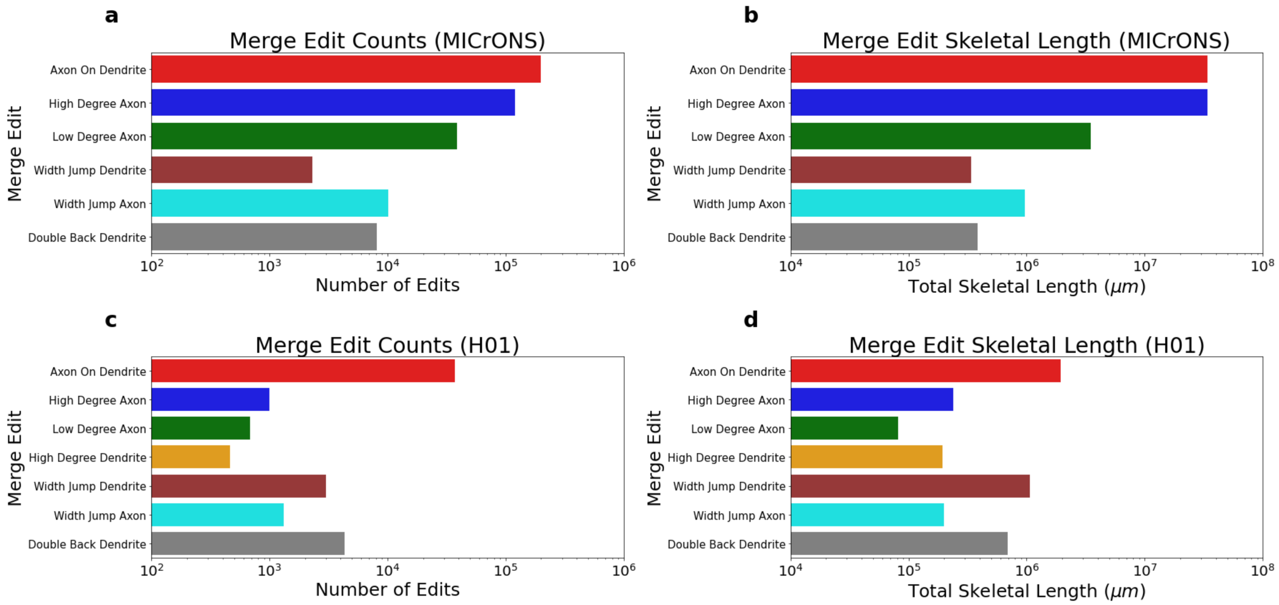

Supplemental Fig. 5. Counts and total skeletal length of merge errors corrected during automated proofreading. The different heuristic rules are presented from top to bottom in the order that they are implemented in the automated proofreading workflow. All error segments identified are then returned to the set of non-nucleus-associated fragments and are not included in the morphological or connectivity analyses. Note that errors identified by rules later in the workflow are excluded from these statistics if they are found on already-errored segments identified earlier in the workflow. **a** (MICrONS) Total number of separate locations where a specific heuristic rule corrected a merge error. **b** (MICrONS) Total skeletal length eliminated by each heuristic rule. **c** (H01) Total number of separate locations where a specific heuristic rule corrected a merge error. **d** (H01) Total skeletal length eliminated by each heuristic rule.

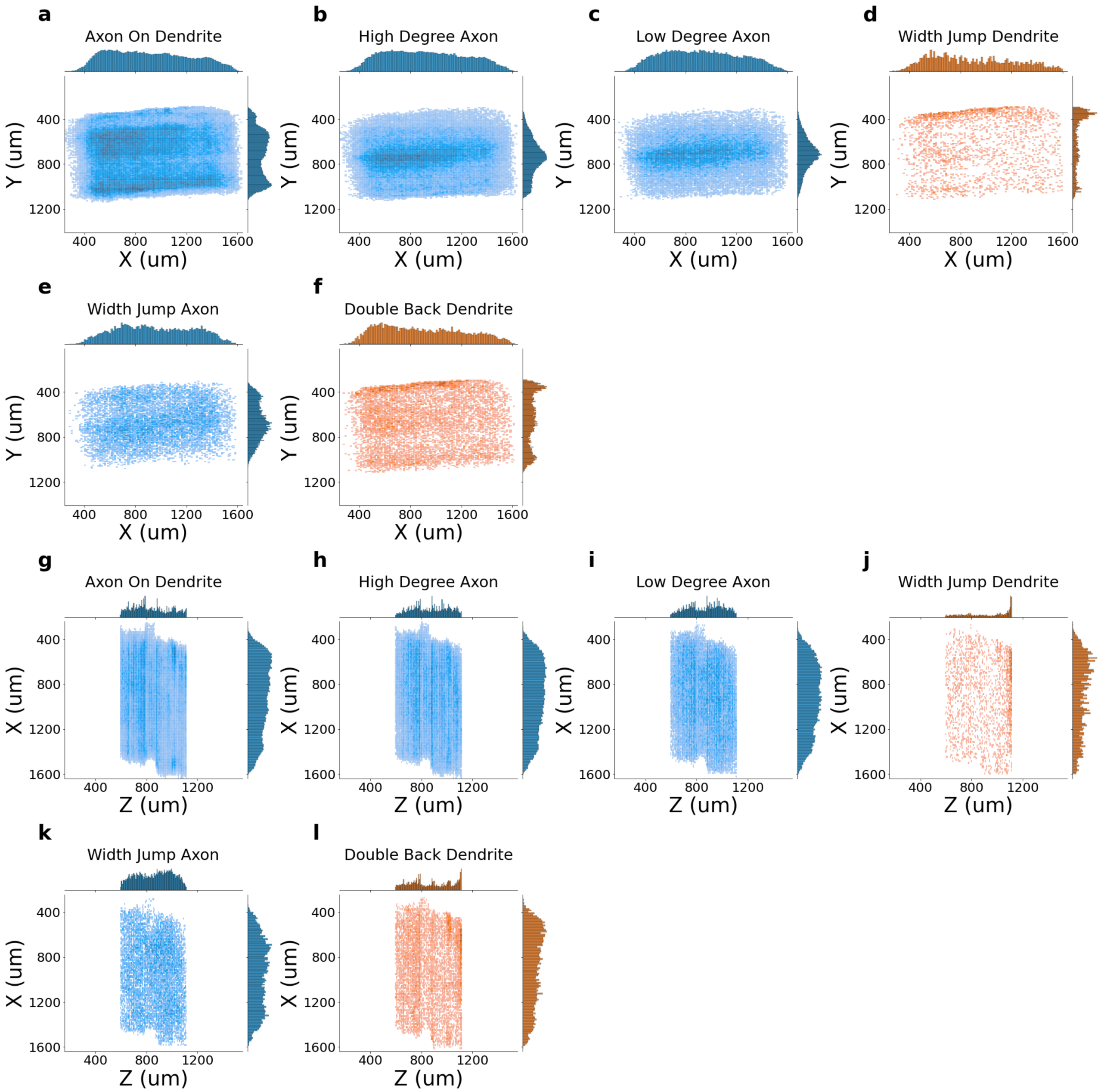

Supplemental Fig. 6. Spatial Distribution of MICrONS Merge Edit Locations. The distribution of locations show biases in the volume for certain types of merge edits. These spatial biases may be due to segmentation or slicing defects, or differences in the concentration of different kinds of neuropil throughout the volume. **a - f** X,Y merge edit locations for different heuristic rules **g - l** X,Z merge edit locations for different heuristic rules

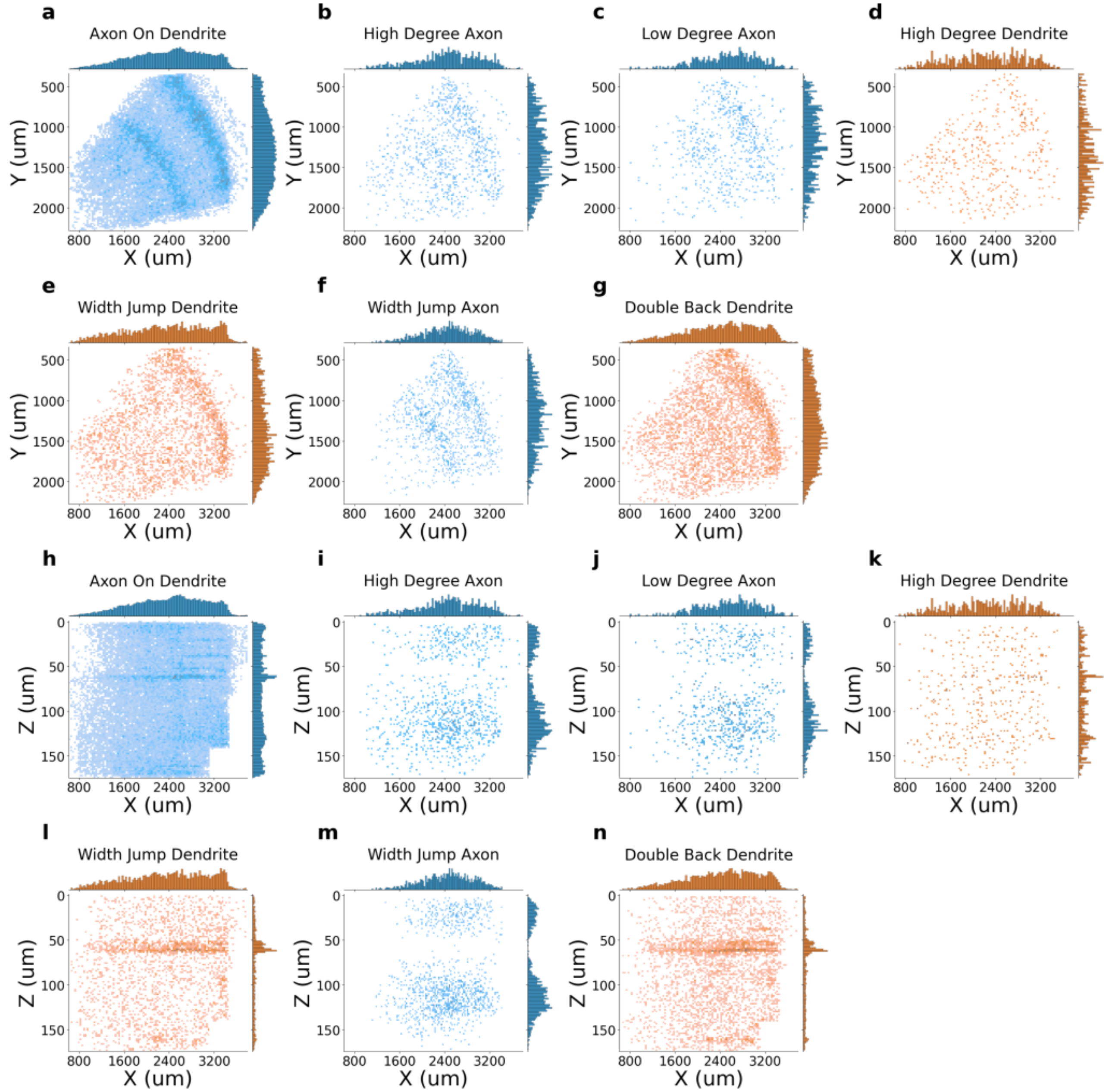

Supplemental Fig. 7. Spatial Distribution of H01 Merge Edit Locations. The distribution of locations show biases in the volume for certain types of merge edits. These spatial biases may be due to segmentation or slicing defects, or differences in the concentration of different kinds of neuropil throughout the volume. **a - g** X,Y merge edit locations for different heuristic rules **h - n** X,Z merge edit locations for different heuristic rules

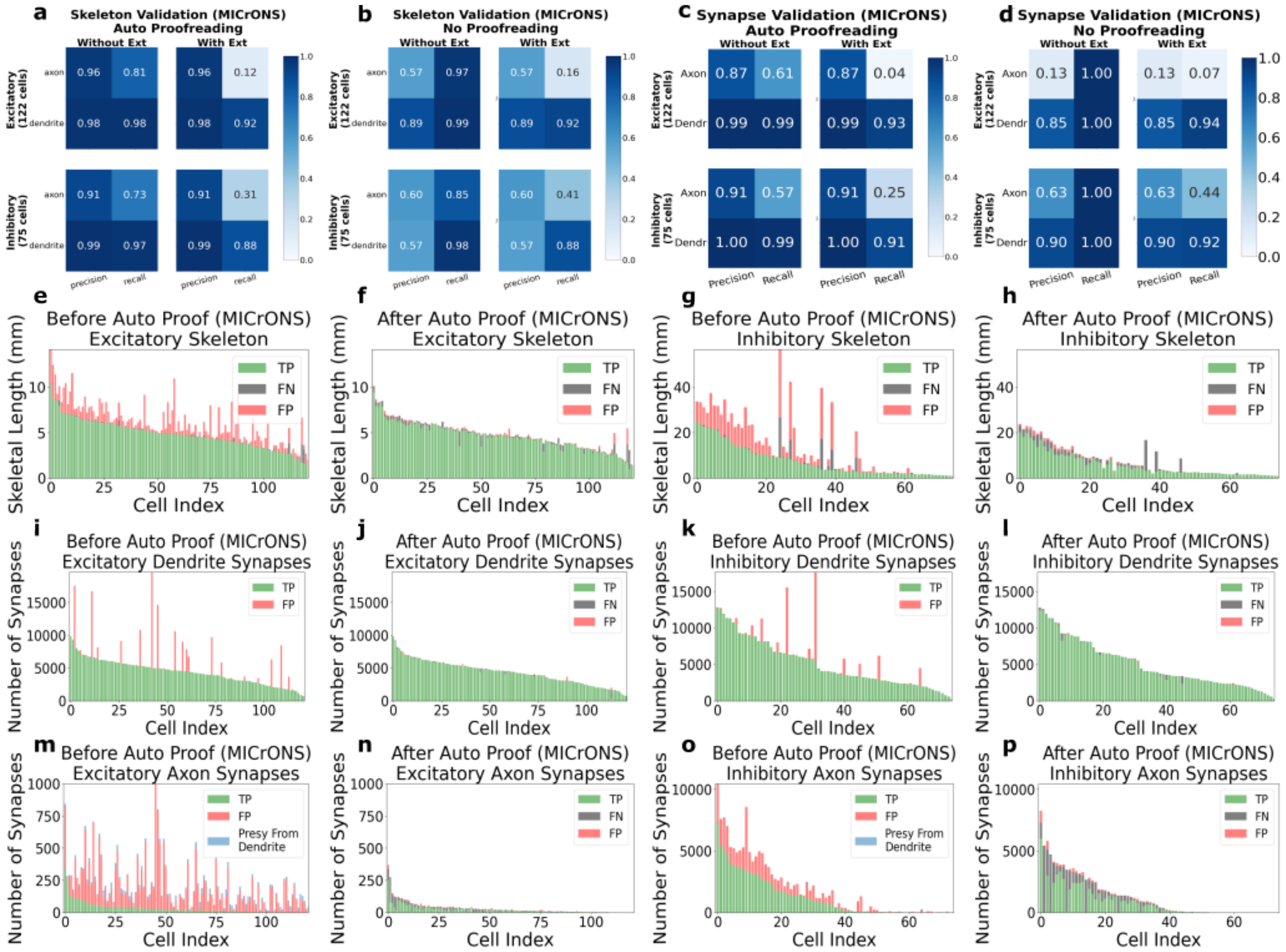

Supplemental Fig. 8. Supplemental MICrONS Auto Proofreading Validation. Validation metrics and visualizations of the automatic proofreading step when comparing the edits made by the automatic proofreading algorithm to edits made by human proofreaders. The two different columns ("With Ext" and "Without Ext") for the confusion matrices in panels a - d represent comparisons with two possible sources of human proofreading ground truth. The "With Ext" column refers to the skeleton or synapse state after automatic proofreading (panels a,c) or in the raw un-proofread segmentation (panels b,d) compared to the state after a human proofreader both cleaned the existing cell of merge errors and added back missing axon and dendrite segments. The "Without Ext" column performs this same comparison, but assuming that human proofreaders ONLY cleaned merge errors (without performing any extensions). The number of cells in the test set were 122 excitatory and 75 inhibitory. Histograms (panels e - p) give a visual representation of the metrics reported in the precision/recall tables (panels a - d). False Negative (FN) classifications can exist before automatic proofreading because of dropping axon/dendritic segments in the mesh and graph processing pipeline prior to the automatic proofreading step. Note: neurons with multi-soma merges are included in these visualizations and metrics. **a** The precision/recall metrics comparing the skeleton length of cells after automatic proofreading for the Axon/Dendrite compartments and for different exc/inh cell types when compared to human proofreading with extension and without. **b** The precision/recall metrics comparing the skeleton length of cells with no automatic proofreading. **c** The precision/recall metrics comparing the synapse counts of cells with automatic proofreading. **d** The precision/recall metrics comparing the synapse counts of cells with no proofreading. **e - h** TP/FN/FP classification of each test cell's skeletons before and after automatic proofreading for both excitatory and inhibitory cells, demonstrating a large percentage of the FP skeleton segments are removed after the process. **i - l** TP/FN/FP classification of each test cell's dendrite synapses (postsyns) before and after automatic proofreading for both excitatory and inhibitory cells, demonstrating a large percentage of the FP postsyns are removed after the application of dendrite proofreading heuristics. **m - p** TP/FN/FP classification of each test cell's axon synapses (presyn) before and after automatic proofreading for both excitatory and inhibitory cells, demonstrating a large percentage of the FP presyns are removed after the application of axon proofreading heuristics. Those axon presyns located not on the main axon but on dendritic segments are filtered away and designated as "Presyn From Dendrite", which does not include the heuristic rule of "Axon on Dendrite" but instead just filters away any presyns located on dendritic segments that were not filtered away using the heuristic rules.

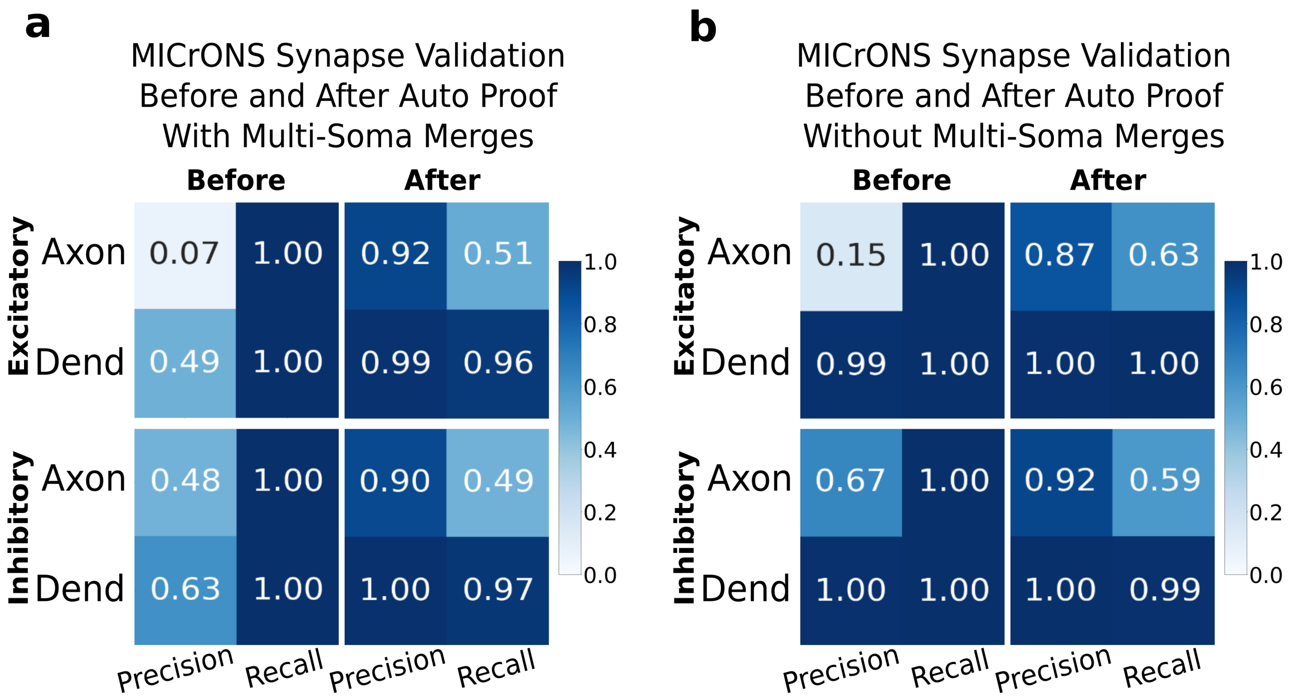

Supplemental Fig. 9. Supplemental MICrONS Multi-Soma Auto Proofreading Validation. The precision/recall metrics comparing the synapse counts of cells before and after automatic proofreading for the Axon/Dendrite compartments and for different excitatory/inhibitory cell types when compared to human proofreading. **a** Validation when only considering neurons with at least one soma merge to the main segment (19 excitatory, 12 inhibitory). **b** Validation when only considering neurons with no soma merged to the main segment (103 excitatory, 63 inhibitory).

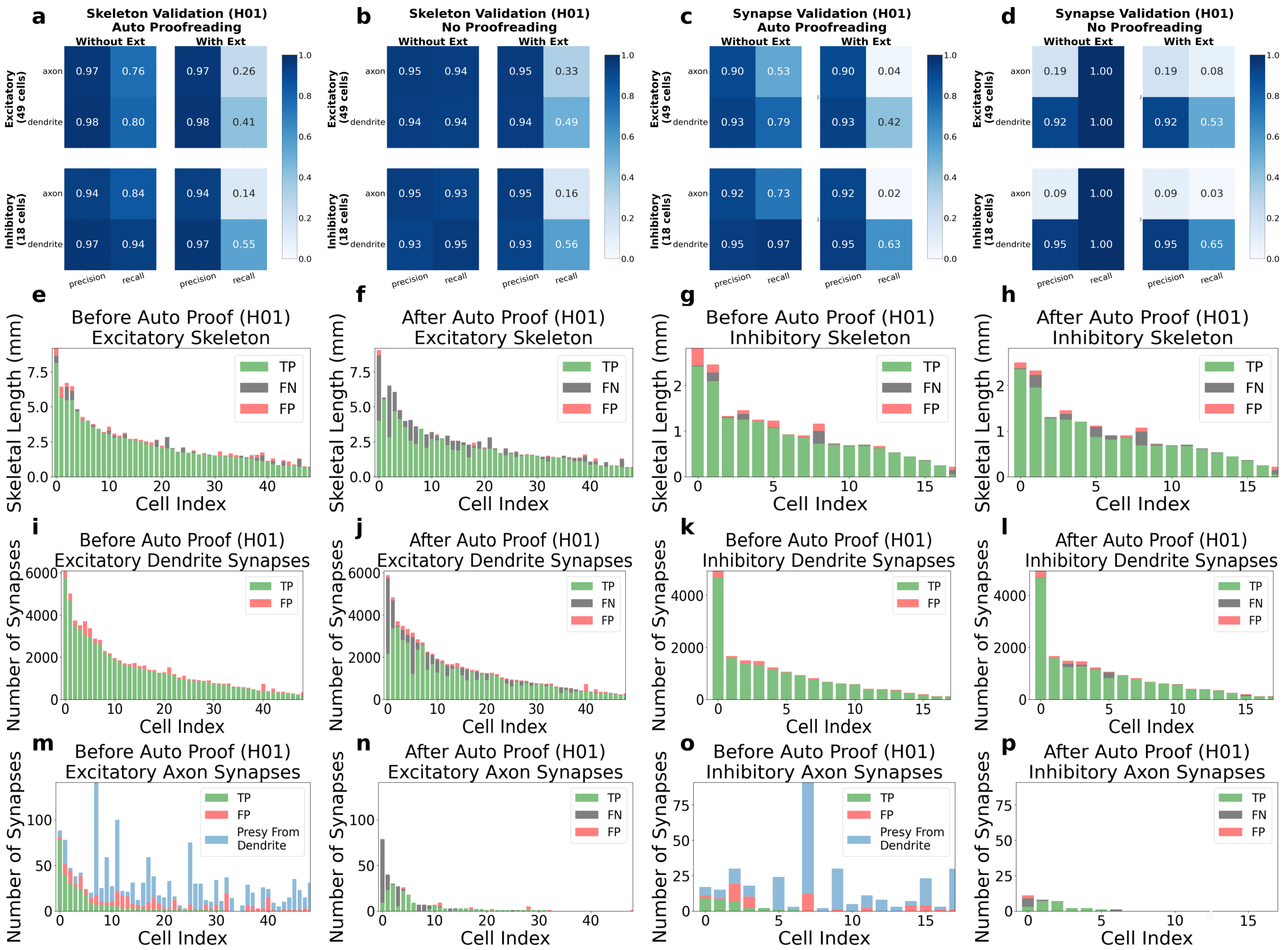

Supplemental Fig. 10. Supplemental H01 Auto Proofreading Validation. Validation metrics and visualizations of the automatic proofreading step when comparing the edits made by the automatic proofreading algorithm to edits made by human proofreaders. The two different columns ("With Ext" and "Without Ext") for the confusion matrices in panels a - d represent comparisons with two possible sources of human proofreading ground truth. The "With Ext" column refers to the skeleton or synapse state after automatic proofreading (panels a,c) or in the raw un-proofread segmentation (panels b,d) compared to the state after a human proofreader both cleaned the existing cell of merge errors and added back missing axon and dendrite segments. The "Without Ext" column performs this same comparison, but assuming that human proofreaders ONLY cleaned merge errors (without performing any extensions). The number of cells in the test set were 49 excitatory and 18 inhibitory. Histograms (panels e - p) give a visual representation of the metrics reported in the precision/recall tables (panels a - d). FN classifications can exist before automatic proofreading because of dropping axon/dendritic segments in the mesh and graph processing pipeline prior to the automatic proofreading step. Note: While perfectly extending all axonal and dendritic processes is not yet possible, the extent to which neurons were extended in the manually proofread set from the H01 dataset are much less extensively extended in comparison to those of the MICrONS dataset; therefore, the recall numbers for the "With Ext" categories in the H01 validation are much more likely an over-estimate in comparison with those of the MICrONS dataset. **a** The precision/recall metrics comparing the skeleton length of cells after automatic proofreading for the Axon/Dendrite compartments and for different exc/inh cell types when compared to human proofreading with extension and without. **b** The precision/recall metrics comparing the skeleton length of cells with no automatic proofreading. **c** The precision/recall metrics comparing the synapse counts of cells with automatic proofreading. **d** The precision/recall metrics comparing the synapse counts of cells with no proofreading. **e - h** TP/FN/FP classification of each test cell's skeletons before and after automatic proofreading for both excitatory and inhibitory cells, demonstrating a large percentage of the FP skeleton segments are removed after the process. **i - l** TP/FN/FP classification of each test cell's dendrite synapses (postsyns) before and after automatic proofreading for both excitatory and inhibitory cells, demonstrating a large percentage of the FP postsyns are removed after the application of dendrite proofreading heuristics. **m - p** TP/FN/FP classification of each test cell's axon synapses (presyn) before and after automatic proofreading for both excitatory and inhibitory cells, demonstrating a large percentage of the FP presyns are removed after the application of axon proofreading heuristics. Those axon presyns located not on the main axon but on dendritic segments are filtered away and designated as "Presyn From Dendrite", which does not include the heuristic rule of "Axon on Dendrite" but instead just filters away any presyns located on dendritic segments that were not filtered away using the heuristic rules.

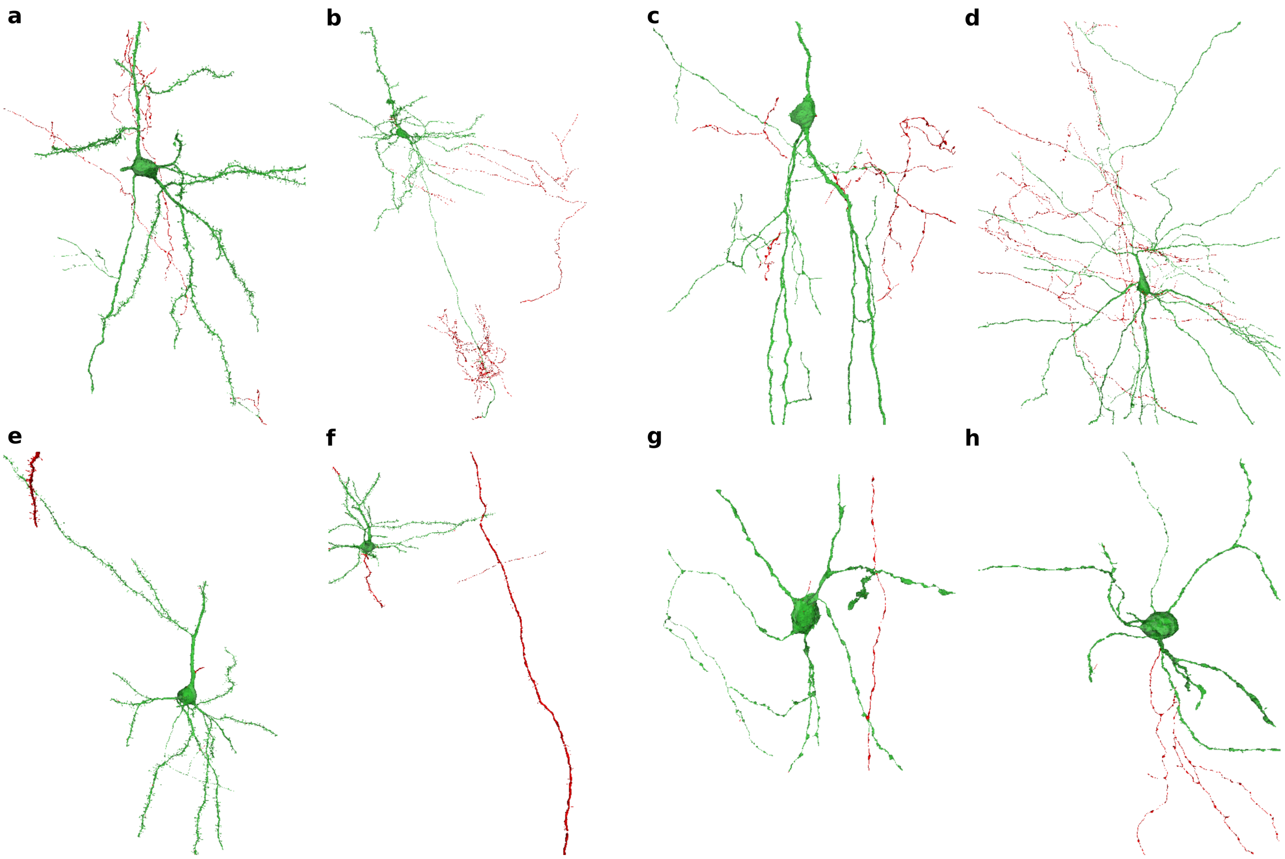

Supplemental Fig. 11. Proofread Neuron Examples with Merge Errors Labeled Examples of excitatory and inhibitory neurons from both the MICrONS and H01 dataset after automatic proofreading (green) with the removed merge errors shown (red). **a,b** (MICrONS) Example excitatory cells in the 50th and 90th percentile of merge error skeletal length removed. **c,d** (MICrONS) Example inhibitory cells in the 50th and 90th percentile of merge error skeletal length removed. **e,f** (H01) Example excitatory cells in the 50th and 90th percentile of merge error skeletal length removed. **g,h** (H01) Example inhibitory cells in the 50th and 90th percentile of merge error skeletal length removed.

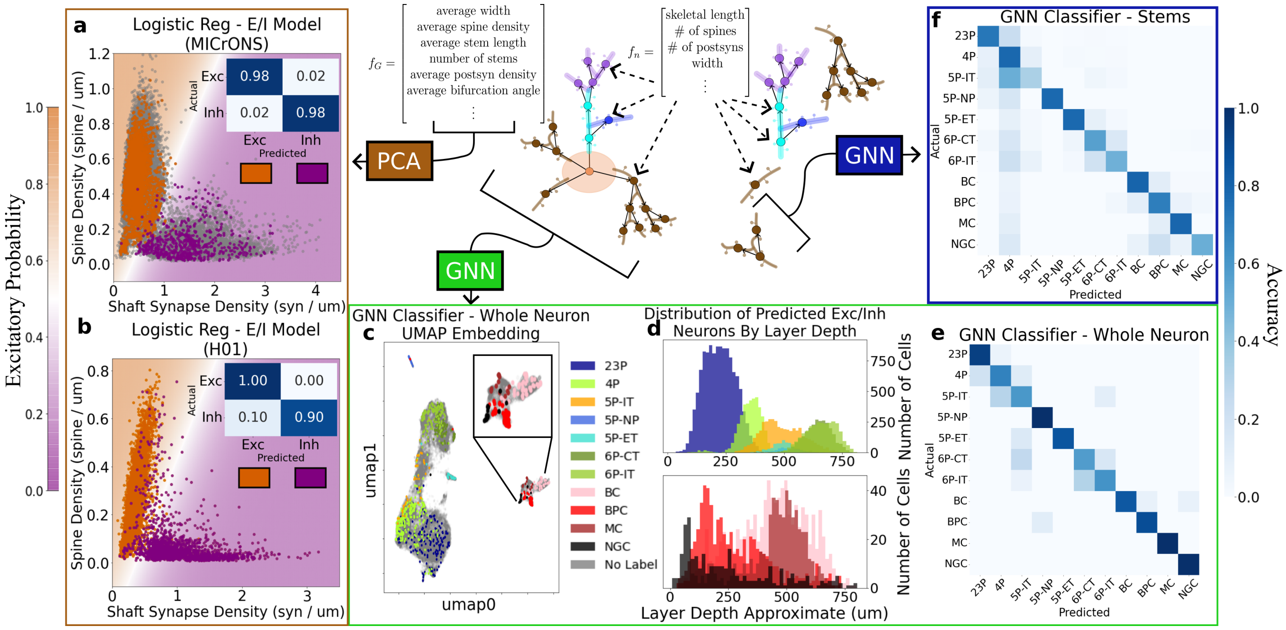

Supplemental Fig. 12. Graph decomposition enables cell-type classification. a,b Two interpretable features identified by PCA were highly informative for excitatory/inhibitory classification: spine density (number of spines per μm of skeletal length) and shaft synapse density (number of synapses not on a spine per μm of skeletal length). Consistent with previous studies[19,20], a logistic regression model trained on just these two features enabled linear discrimination of excitatory and inhibitory cells with high accuracy (same parameters for logistic regression model used for both MICrONS and H01 dataset). **c** A Graph Convolutional Neural Network (GCN) trained on manually-annotated cell types produces an embedding space with a continuum of excitatory neurons progressing from the top layers down to the bottom layers while keeping inhibitory neurons and some excitatory neurons with distinct morphology (5P-NP and 5P-ET) clearly separated (see Supplemental Table ?? for the cell-type abbreviation glossary). **d** The depth of predicted cell types outside of the training volume are consistent with their expected laminar distribution even though no coordinate features are used in the GCN classifier. **e** Confusion Matrix of the test dataset for the neuron GCN classifier tested on n=178 held out neurons: 23P (n=33), 4P (n=51), 5P-IT (n=10), 5P-NP (n=4), 5P-ET (n=7), 6P-CT (n=19), 6P-IT (n=29), BC (n=13), BPC (n=9), MC (n=1), NGC (n=2). **f** Cell classes could also be determined using a dendritic subgraph of only one stem (branching segment connected to the soma) nearly as well as when using the entire dendritic tree, suggesting that the GCN identifies somewhat local features that enable classification. GCN tested on n=1023 test stems: 23P (n=230), 4P (n=301), 5P-IT (n=48), 5P-NP (n=18), 5P-ET (n=45), 6P-CT (n=116), 6P-IT (n=137), BC (n=65), BPC (n=30), MC (n=19), NGC (n=14).

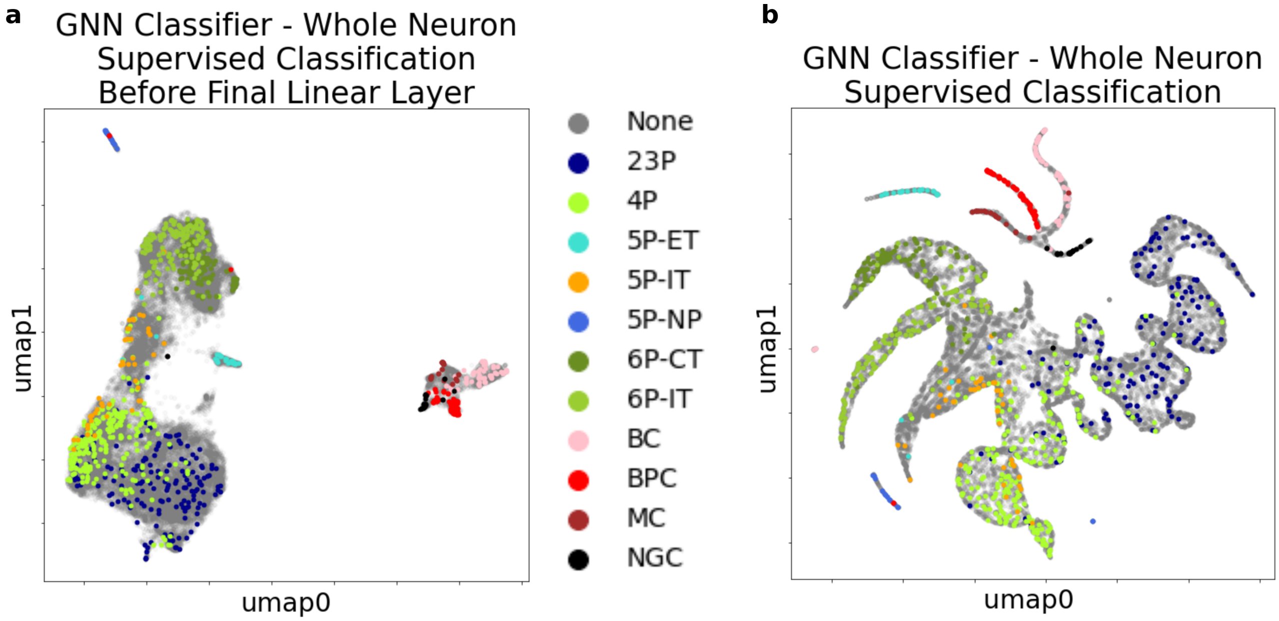

Supplemental Fig. 13. GNN Classifier Whole Neuron UMAP Embeddings. a Embeddings before the final linear layer and softmax function with hand labeled cells from [16] overlaid (these labels were used for the training and validation process of the GNN). Cell-type separation is evident at this stage indicating that the classifier has learned useful features prior to the readout. See Supplemental Table ?? for the cell-type abbreviation glossary. **b** Embeddings after the final linear layer with cell type labels from [16].

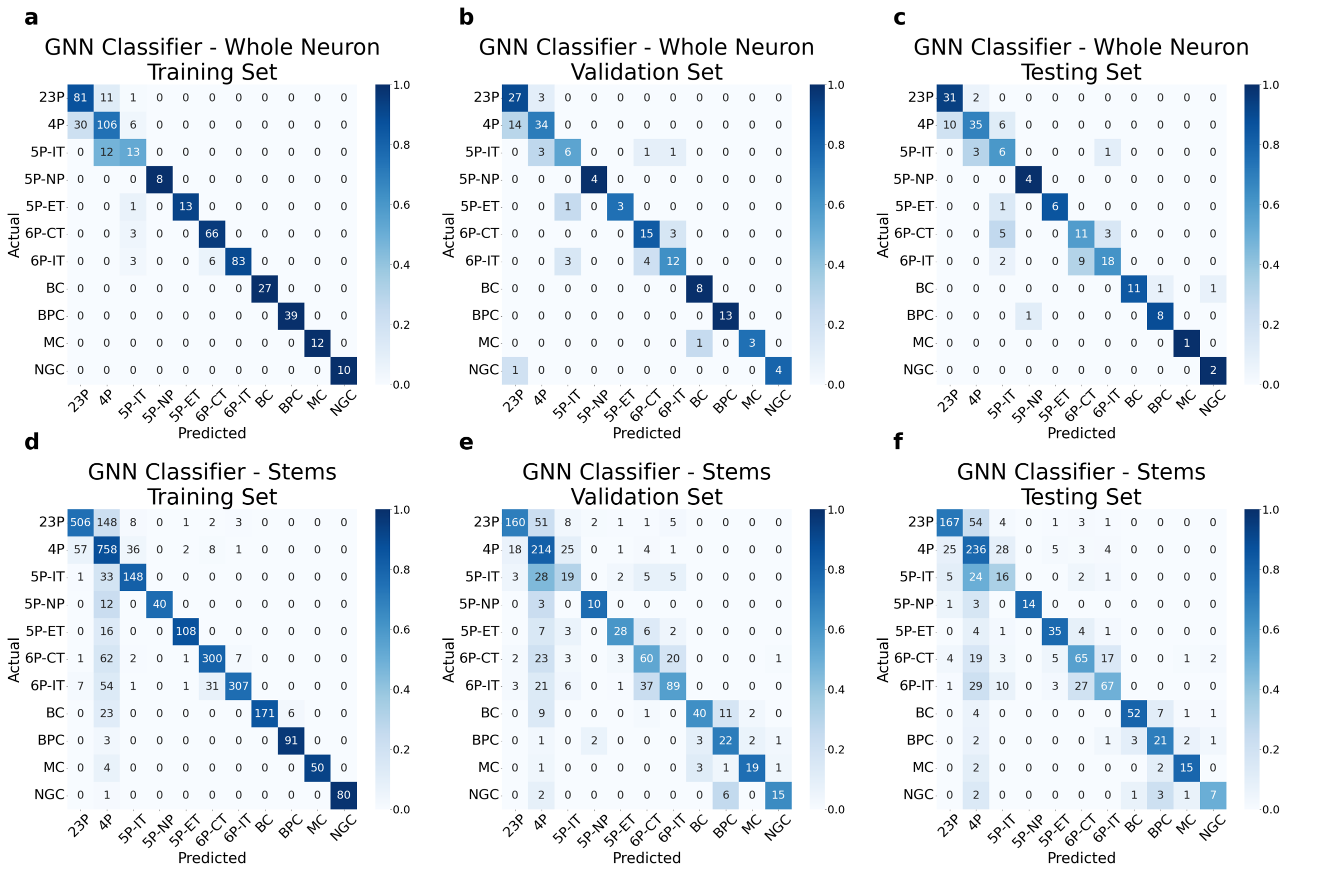

Supplemental Fig. 14. GNN Classifier Train/Validation/Test Confusion. Confusion matrix of predicted and actual neuron counts from each of the 60:20:20 Train/Validation/Test splits used in the supervised training process of the Graph Neural Network cell type classifier. All ground truth labels were from hand-annotated classes described in[16]. Color intensities are from normalized values in reference to the summation of a given row. See Supplemental Table ?? for the cell-type abbreviation glossary. **a-c** Confusion matrix (neuron counts) for the GNN Classifier using the entire neuron graph including the somatic root node. **d-f** Confusion matrix (stem counts) for the GNN Classifier applied to a single stem (a single projection from the soma).

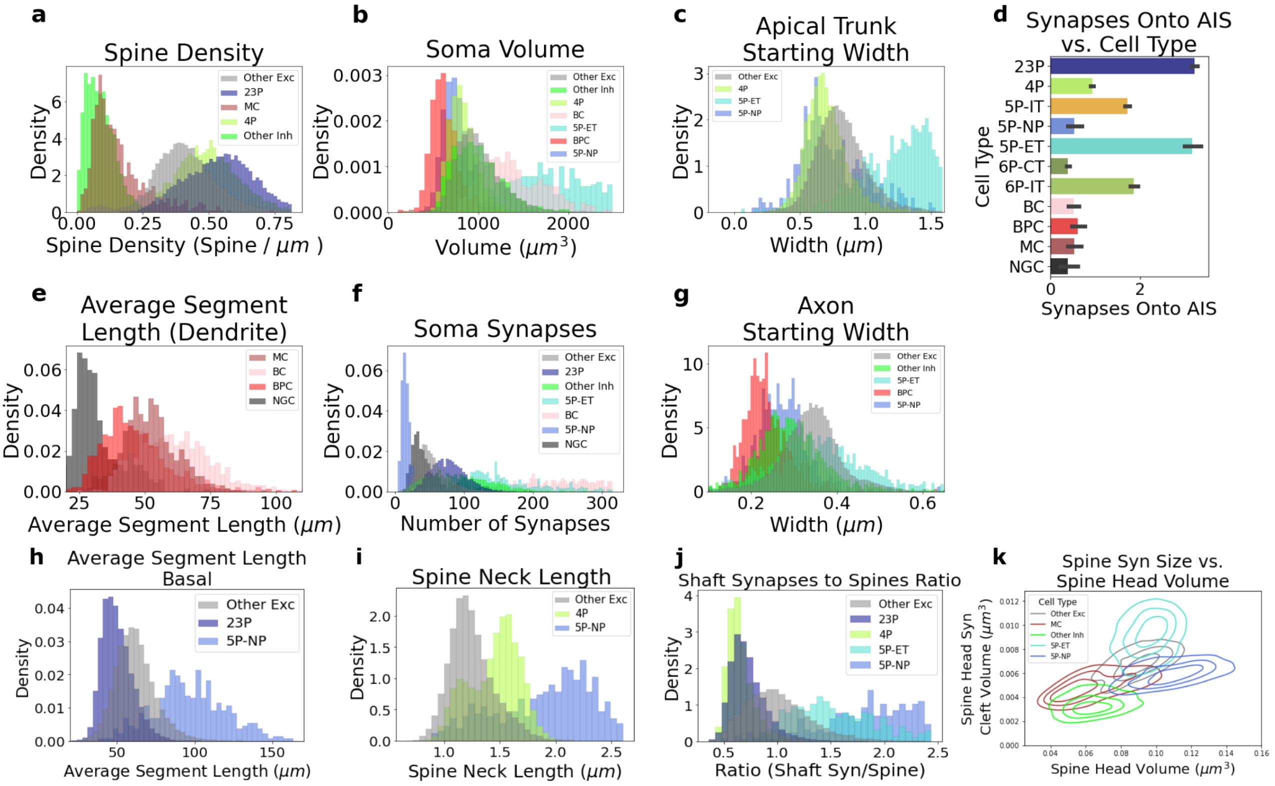

Supplemental Fig. 15. Various morphological features computed by NEURD. Histograms and bar graphs of a variety of salient features computed by NEURD, colored by the labels generated from the GNN classifier. Some plots are replicating previous work from[21]. See Supplemental Table ?? for the cell-type abbreviation glossary. **a** Spine density (number of spines per μm of skeletal length) distributions from automatic spine detection. As has been previously reported[19], layer 2/3 pyramidal cells are more densely spiny than layer 4, and MC spine density is higher than other inhibitory cells. **b** Soma volume computed during the soma detection step in the NEURD mesh processing pipeline. As expected, 5P-NP, 4P and BPC generally have smaller somas than other cells from their same excitatory or inhibitory class while 5P-ET and BC are larger than other cells in the same class[21]. **c** Width measurements generated from the average distance of the inner skeleton to the mesh surface (radius approximation) at the beginning of the apical trunk protrusion. Compared to other cell types, 4P and 5P-NP cells have smaller trunks, while 5P-ET are larger. **d** Mean number of synapses (+/- SD) onto the axon initial segment (AIS) for different cell types. As expected, 23P, 5P-ET, and 5P-IT cell types are more densely innervated on their AIS[22]. AIS is defined as within 10 μm - 40 μm skeletal distance of the soma, and error bars are standard deviation. **e** Average skeletal length of non-branching dendritic segments for stems of different cell type subclasses, illustrating that NGC have significantly shorter distances between branch points in their dendrites than other inhibitory cells. **f** Distributions of synapses onto the soma illustrating the expected larger average number of soma synapses for 5P-ET and BC and smaller numbers for 5P-NP and NGC[21]. **g** Distributions of radius approximation for the start of the axon protrusion from either a dendrite or the soma, showing smaller typical widths for 5P-NP and BPC and larger starting widths for 5P-ET. **h** Distribution of the average skeletal length of non-branching dendritic segments for stems of different cell-type subclasses. **i** Distribution of average spine neck length of different excitatory cell type subclasses. As expected, 5P-NP cells have the longest average neck lengths, but 4P cells also display significantly longer necks than other excitatory cell types. **j** The ratio of non-spine synapses to spine counts varies across cell types. **k** KDE plots (kernel density estimation, estimates the shape and distribution of the discrete dataset, quartile levels shown) of spines on different cell type subclasses, comparing each spine head's volume and the size of the largest synapse on that spine head. These plots reveal differences in both the distribution and scaling of synapse sizes and spine heads across cell types. For example Martinotti cells (red) have larger spine head synapses than other inhibitory spines (light green) and the head volume and synapse size scale at a rate more similar to other excitatory cells (grey).

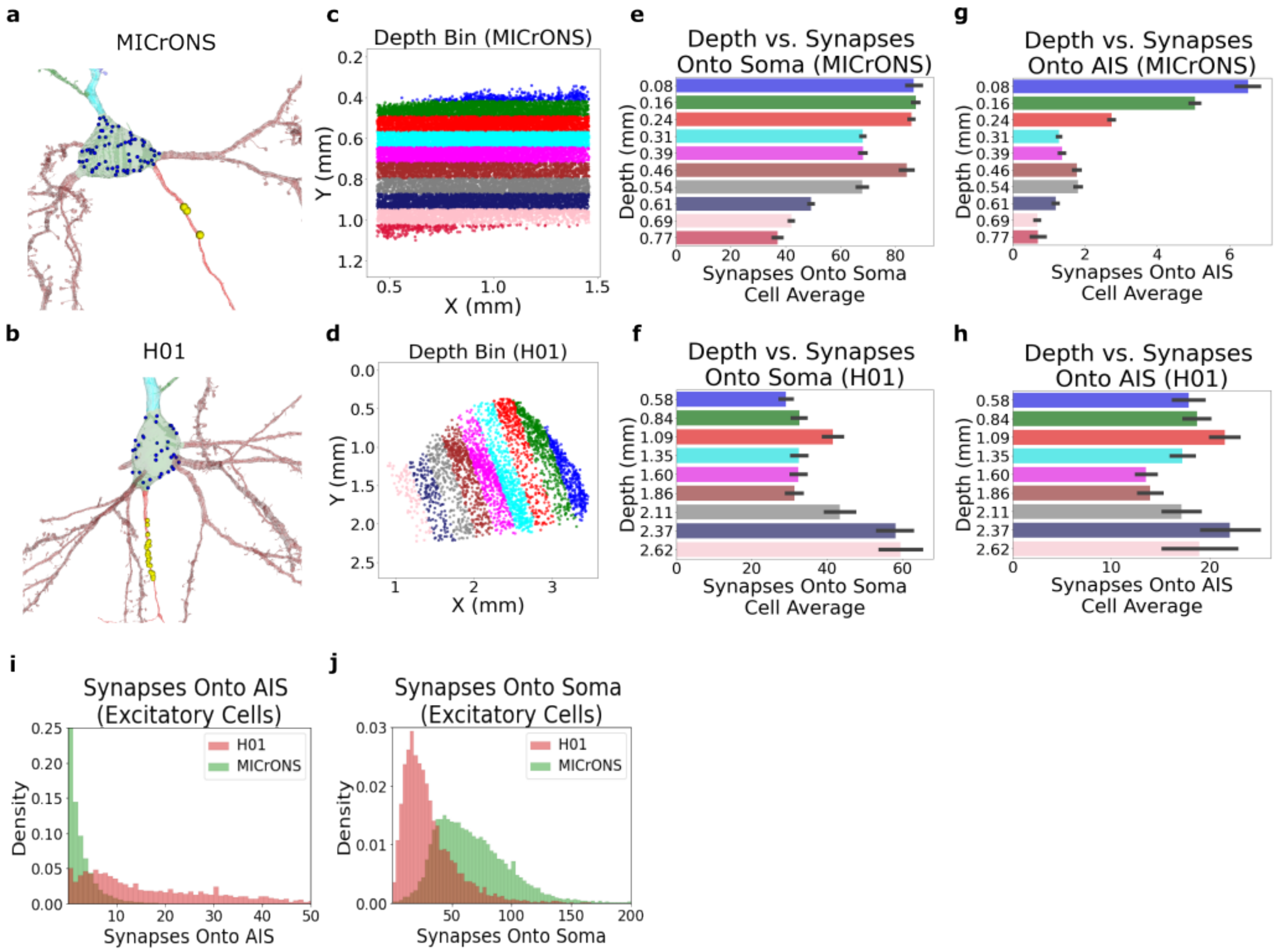

Supplemental Fig. 16. Comparison of Synapses onto AIS and Soma a-b Example neurons with synapses onto the axon (AIS synapses) in yellow and synapses onto soma in blue. Example neurons are both in the 75th percentile of the number of AIS synapses in their respective volumes. **c,d** Depth bins used for analysis of both synapses onto AIS vs depth and synapses onto soma vs. depth (this figure, panels e-h and Fig. 4a). Note, for the MICrONS dataset plots there is an offset of approximately 300 μm between the depth value and the y coordinate. **e,f** Average number of synapses onto the soma of cells varies across depth (mean +/- SD), decreasing in deeper layers of the MICrONS volume, but increasing in deeper layers of the H01 dataset. **g,h** Average number of synapses onto the axon initial segment (AIS) of cells at different laminar depths (mean +/- SD) for the MICrONS and H01 volume. **i** Distribution of the number of AIS synapses per cell compared across datasets, emphasizing the increased innervation of the AIS for neurons in the H01 dataset in comparison to MICrONS. **j** Distribution of the number of soma synapses per cell. As expected, neurons in the MICrONS volume have more identified synapses onto their soma, despite the smaller surface area of mouse somas compared to human[23].

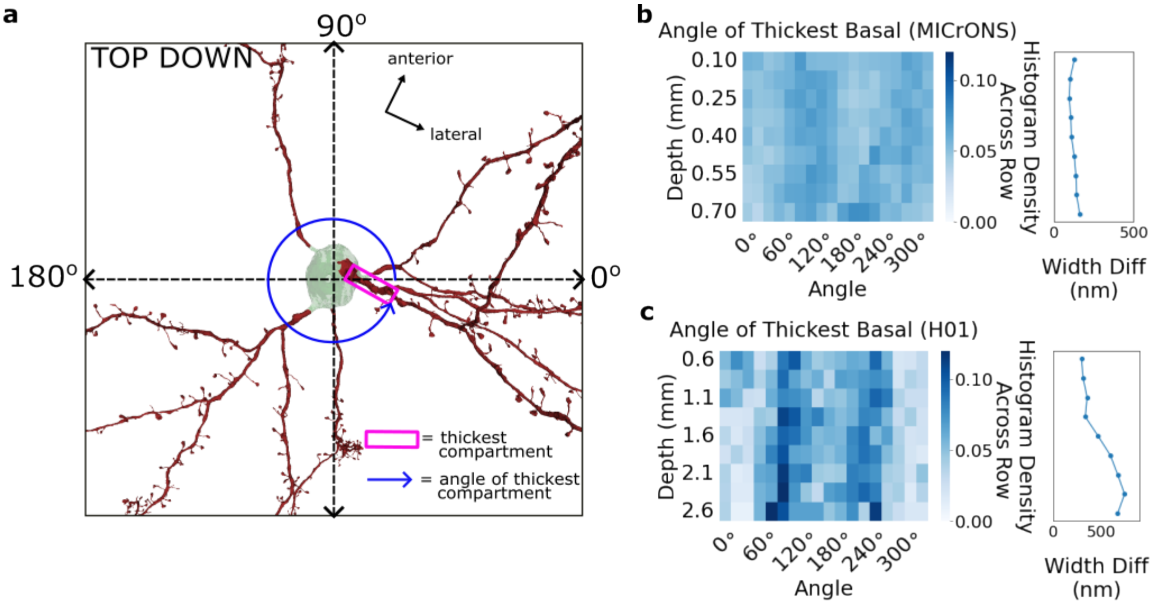

Supplemental Fig. 17. Thickest Basal Geometric Analysis. Using the easily accessible geometric features of neurons after decomposition, the thickest basal branch is identified and the distribution of xz projection angles for all neurons in the volume is compared across depths for the two volumes. **a** Top down view of a neuron to illustrate the geometric analysis: the thickest basal branch is boxed in pink and the xz angle of these branches are indicated with a blue circular angle marker. **b,c** Histograms showing the distribution of mean skeletal angle of the thickest basal stem. Each row is a normalized histogram for a specific depth bin. The H01 dataset shows more bimodal structure (especially in the deeper layers) than the MICrONS dataset, consistent with a previous report[5]. We find that this pattern is also visible in more superficial layers of H01, but is less obvious because the width difference between the widest and the second-widest basal branch is much larger in deep layers ("Width Diff" plots to the right of the heat maps).

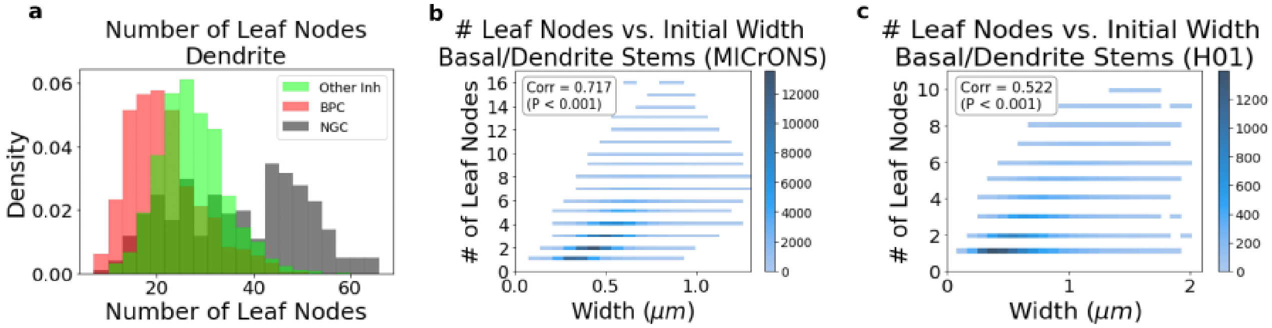

Supplemental Fig. 18. Neuron Dendritic Branching Characteristics. Measurements related to leaf nodes (terminating ends of the dendritic stem) excluding apical dendrites. **a** Distributions of the number of total leaf nodes for the non-apical dendrites of each neuron separated by inhibitory cell type. As expected, NGC cells have the most leaf nodes of any inhibitory cell type, while BPC have fewer leaf nodes compared to other interneurons. **b-c** Histogram for all the non-apical dendritic stems of every neuron in the volume comparing the initial width of the stem to the number of leaf nodes. For both the MICrONS (b) and H01 (c), there is a significant positive correlation (Pearson's, $p < 10^{-300} forboth)$).

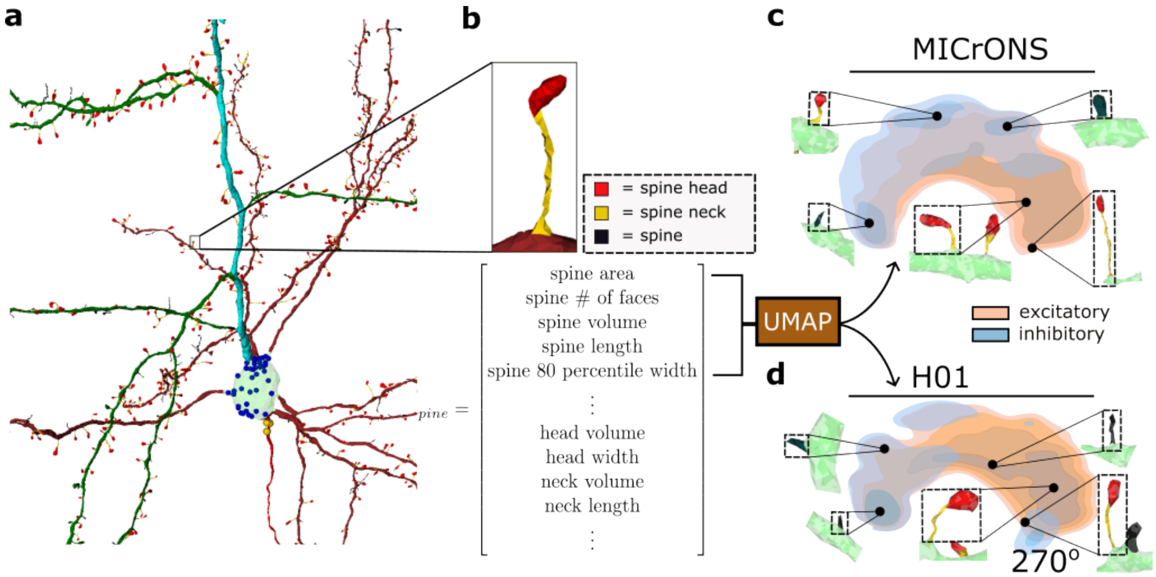

Supplemental Fig. 19. Spine Feature Extraction and UMAP distribution . **a** Cleaned and annotated neuron mesh; soma synapses in blue, axon initial segment (AIS) synapses in yellow, basal dendrite in brown, apical trunk in aqua, oblique branches in green. Spine heads in red, spine necks in yellow and non-segmented spine in black along dendritic segments. An random spine is identified with the black rectangle and expanded in the next panel. **b** Example spine submesh from the spine identification with the head and neck mesh segmentation shown and a vector of features extracted from the spine submesh. Most spines are annotated with interpretable features such as head volume, spine skeletal length, and spine neck length while some spines (shown in black) which are smaller (typically under 0.7 um) or unable to be segmented, lack the head/neck features. **c,d** Kernel density estimation of UMAP embedding (quartile levels shown) of spines sampled from MICrONS and H01 dataset using spine features from panel **b** (without head or neck features). The embeddings show a similar embedding structure between the two datasets in terms of spine shape and inhibitory/excitatory class, similar to previous work clustering a non-parametric representation of postsynaptic shapes[24].

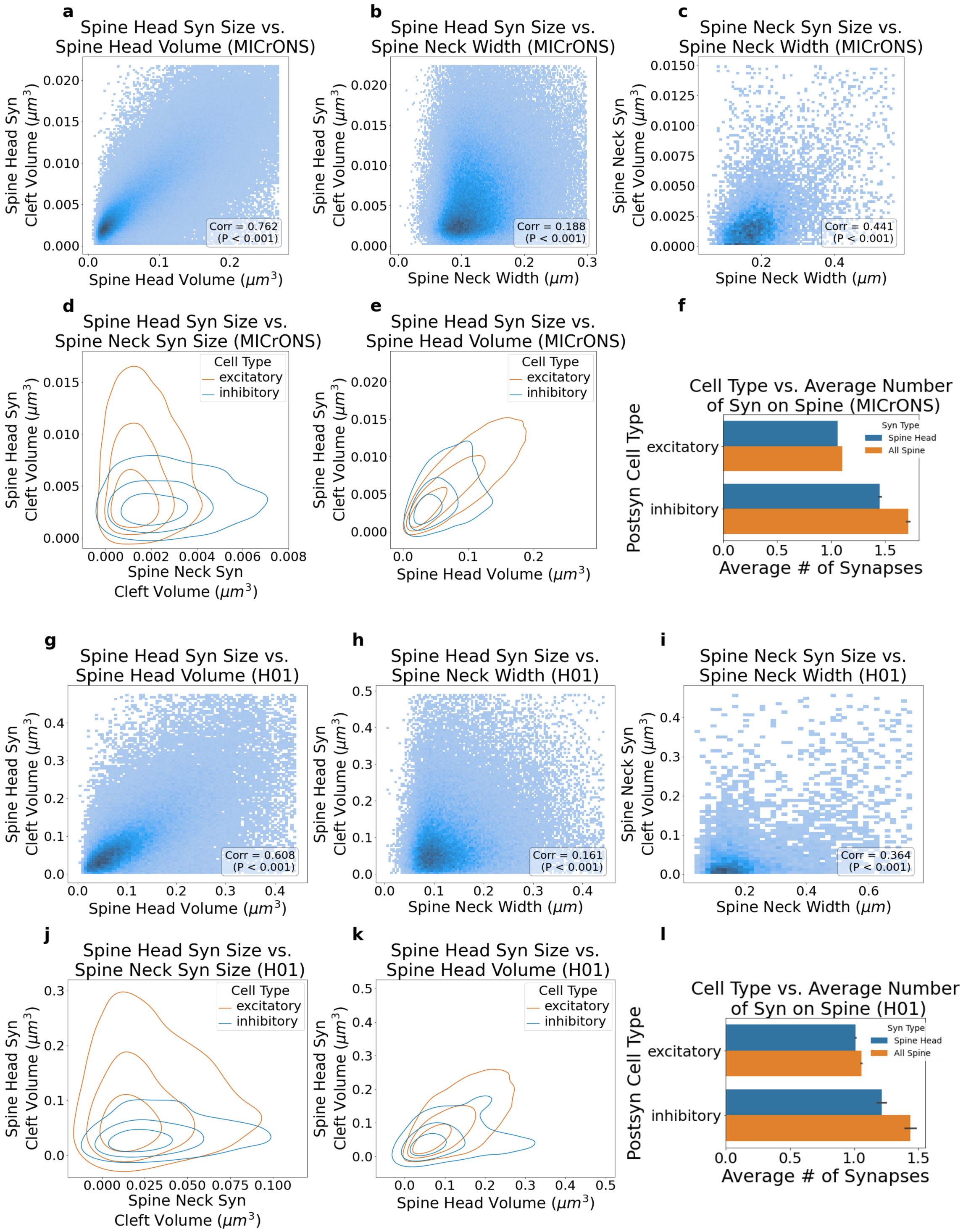

Supplemental Fig. 20. Postsynaptic Spine Feature Analysis Here we compare the distributions and correlations of certain spine features; replicating and expanding on previous work. The MICrONS dataset is analyzed in **a-f** and the same analysis is repeated for H01 in **g-l**. **a-b,g-h** As expected, for synapses onto the spine **head**, the size of the synaptic cleft and the volume of the spine head mesh are strongly positively correlated, while cleft size and neck width are positively but more weakly correlated[25,26] (Pearson's, $p < 10^{-300}$ for all). **c,i** For synapses onto the spine **neck**, the width of the spine neck and the synaptic cleft volume of synapses are positively correlated (Pearson's, $p < 10^{-300}$ for all). **d,j** KDE of the joint distribution (quartile levels shown) of the spine neck synaptic cleft volume with spine head synaptic cleft volume for different postsynaptic cell types (exc/inh), illustrating the different joint distributions for each cell type. For synapses onto spine **heads**, synaptic size has a wider range for excitatory cell spines than inhibitory cell spines in both volumes. For synapses onto spine **necks**, the range of synaptic size is larger for inhibitory cells in the MICrONS volume, but similar in the H01 dataset. **e,k** Spine head volume is positively correlated with spine head synaptic cleft volume for both excitatory and inhibitory neurons in both datasets. **f,l** Average number of synapses (mean +/- SD) on all spines and spine heads for different cell types, indicating that in both datasets inhibitory spines receive more synapses per spine than excitatory spines.

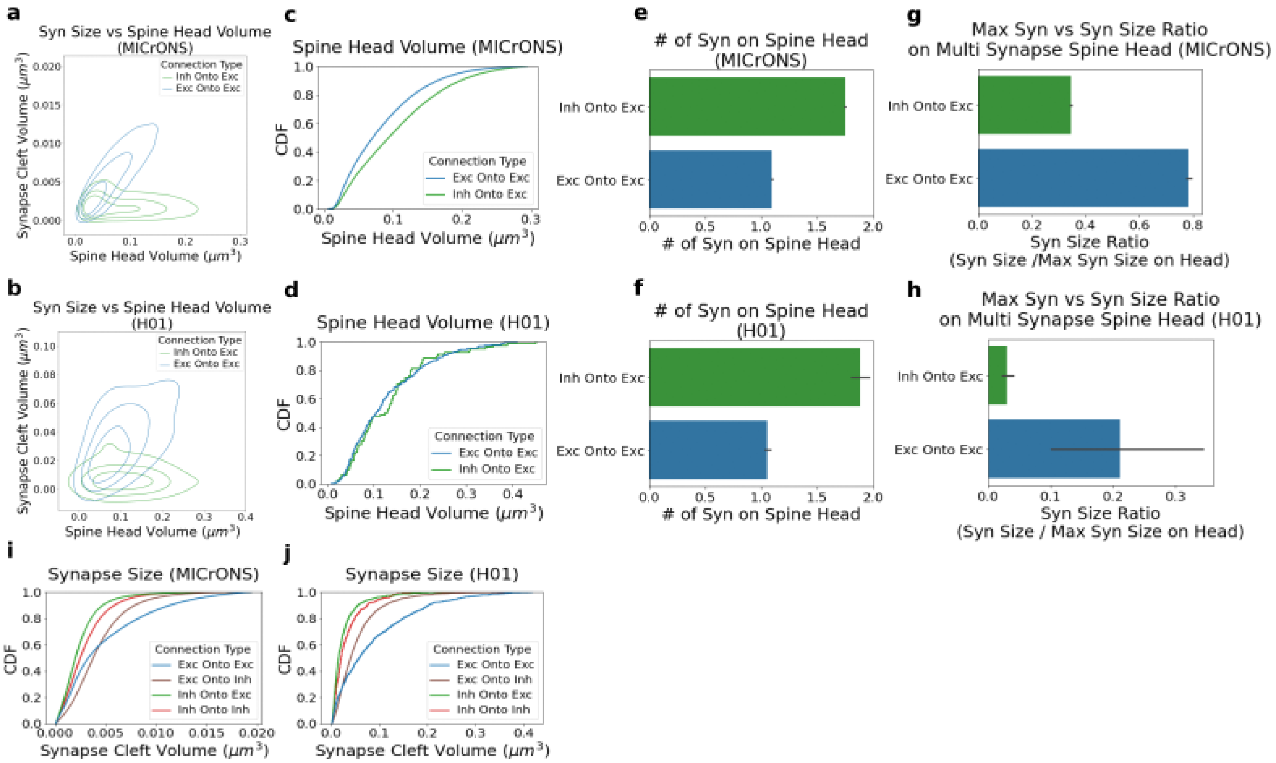

Supplemental Fig. 21. Spine and Synapse Connectivity Analysis. We revisited the spine analysis in Supplemental Fig. 20 taking into account information about the identity of the presynaptic neuron for each synapse. **a-b** KDE distribution (quartile levels shown) relating the postsynaptic spine head volume and the synapse cleft volume for synapses onto excitatory cells given different presynaptic cell types. In both datasets a significant positive correlation between spine head volume and synapse size is observed only when the source cell is excitatory but not when the source is inhibitory. **c-d** CDF of the spine head volume for postsynaptic excitatory cells given different presynaptic cell types. For the MICrONS dataset, inhibitory presynaptic cells typically target larger spine heads but this trend is not significant in H01. **e-f** As a possible explanation of why inhibitory cells target larger spine heads, a plot of the average number of synapses (mean +/- SD) on a spine head conditioned on the presynaptic cell type shows that spine heads targeted by inhibitory neurons generally have two synapses as opposed to a mean closer to one synapse per spine for excitatory synapses. **g,h** Expanding on the observation that inhibitory cells typically synapse onto spines with more than one synapse, for spines with multiple synapses, we plot the relative size of a spine head synapse to the size of the largest synapse on that same spine head (mean +/- SD) given different presynaptic cell types. We observe that the synapse from an inhibitory source is typically much smaller than the largest synapse on the spine head in both the MICrONS and H01 dataset. **i,j** CDF of the distribution of synapse cleft volumes for different connections types show a similar trend between the MICrONS and H01 dataset where the synapses with excitatory presynaptic cells are typically larger than inhibitory cells and synapses onto inhibitory cells are typically smaller than those onto excitatory.

a

Graph	MICrONS (Raw)	MICrONS (Auto)	H01 (Raw)	H01 (Auto)
# nodes (Exc/Inh)	44683 / 5104	44683 / 5104	2085 / 1236	2085 / 1236
# out edges (Exc/Inh)	2608436 / 1462930	263643 / 692756	12567 / 3924	5646 / 1236
mean in degree (Exc/Inh)	76.37 / 129.14	17.76 / 31.89	3.45 / 7.53	0.88 / 4.09
mean out degree (Exc/Inh)	58.38 / 286.62	5.90 / 135.70	6.03 / 3.17	2.71 / 1.00
mean axon sk length (μm)	1044 / 3256	421 / 1397	593 / 329	510 / 251
mean dendr sk length (μm)	3220 / 3150	2618 / 2330	3248 / 1369	2876 / 1229
% nodes in giant component	99.95	99.58	97.68	83.14
mean s.p. (shortest path)	3.61	4.78	9.20	4.37
mean s.p. undirected	3.06	3.87	6.47	9.73
95th percentile s.p.	5.00	7.00	16.00	9.25
95th percentile s.p. undirected	4.00	5.00	11.00	18.00
mean s.p. along Exc edges	3.90	8.33	13.15	1.00
mean s.p. along Exc edges undirected	3.24	5.31	7.39	9.43
mean s.p Exc-Exc/Inh-Inh	3.60 / 3.50	4.80 / 4.43	9.30 / 9.38	5.10 / 1.83
mean s.p Exc-Exc/Inh-Inh undirected	3.06 / 3.00	3.88 / 3.80	6.44 / 6.51	9.68 / 9.75

Supplemental Fig. 22. Connectome Network Statistics Table a Network statistics of the MICrONS and H01 connectomes where the nodes are entire single soma neurons and the edges are the synapses between them (neurons with manual proofreading and their associated synapses are excluded). The term "raw" refers to synaptic data before any processing with NEURD, "giant component" refers to the largest connected subgraph, "auto" refers to the connectome produced after the decomposition pipeline and automated proofreading, "sk" abbreviates "skeletal" when referring to skeletal distances, and "sp" abbreviates "shortest path". Once all neurons were cleaned and annotated from the NEURD decomposition process there were on the order of 10^8 and 10^7 individual presynaptic or postsynaptic connections for the MICrONS and H01 dataset respectively. However, in order for a connection to be included in the connectome, both the presynaptic and postsynaptic information must be present in the data, which was not the case for a majority of connections and is why the number of edges in the connectome is much smaller than number of individual synapses available in the cleaned datasets.

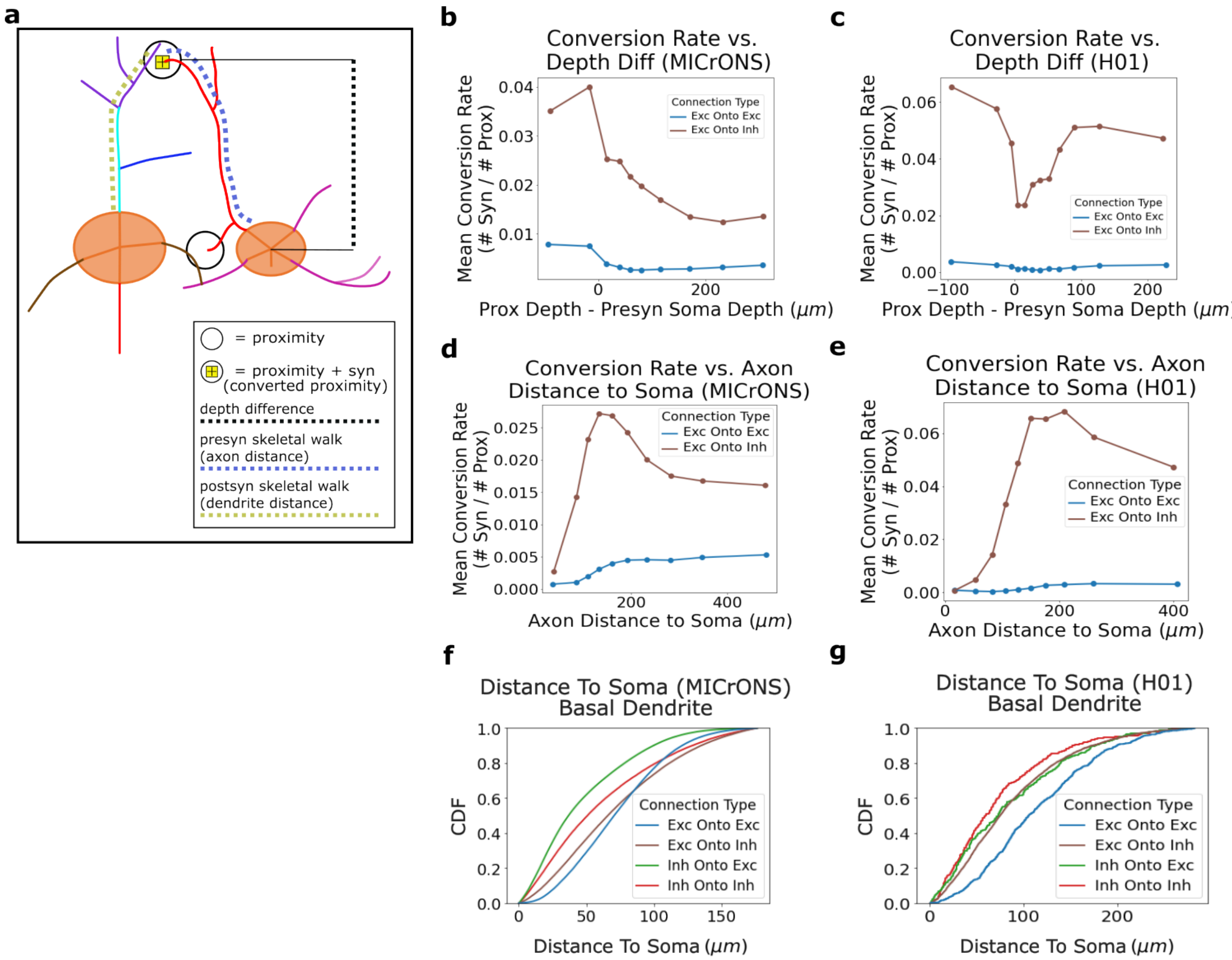

Supplemental Fig. 23. Path distances and relation to conversion conversion rate Features computed by the NEURD preprocessing pipeline make available path lengths and distances along x/y/z dimension or dendritic/axonal walk between presynaptic or postsynaptic soma centers and their synapse or proximity locations. For calculating conversion rates, proximities are binned (approximate equal depth bins) in terms of their relative depth to the presynaptic soma center (proximity depth - presynaptic soma depth) or axonal path distance and then the mean conversion rate (number of synapses / number of proximities) for that bin is computed for different connection types **a** Illustration showing a pair of cells with both a proximity with a converted synapse and one without from an inhibitory cell to an excitatory cell. Each of the paths or distances used in the later panels is shown with a dotted color path: depth difference (proximity depth - presynaptic soma depth; the example drawn would have a negative depth difference because the presynaptic soma is deeper in the volume than the proximity), presynaptic skeletal walk (axonal skeletal distance from the presynaptic soma center to the synapse or proximity), postsynaptic skeletal walk (dendritic skeletal distance from the postsynaptic soma center to the synapse or proximity). **b,c** Conversion rate as a function of relative proximity depth. In the MICrONS volume, the plot demonstrates that the conversion rate for excitatory connections onto both excitatory and inhibitory postsynaptic cells is greater when the proximity is above the soma (for both connection types); in the H01 volume, the plot demonstrates a greater conversion rate above the soma than below, but with an additional reduction in conversion rates close to the soma that is not seen in MICrONS. **d,e** Mean conversion rate as a function of distance from the synapse to the presynaptic cell along the axon. In both the MICrONS and H01 dataset the conversion rate peaks farther away from the soma and then gradually decreases when moving farther downstream. **f,g** Cumulative density function (CDF) of the postsynaptic skeletal walk distance distribution for different exc/inh connection combinations (apical and soma synapses are excluded). In both datasets, excitatory inputs are further along the dendrite from the soma.

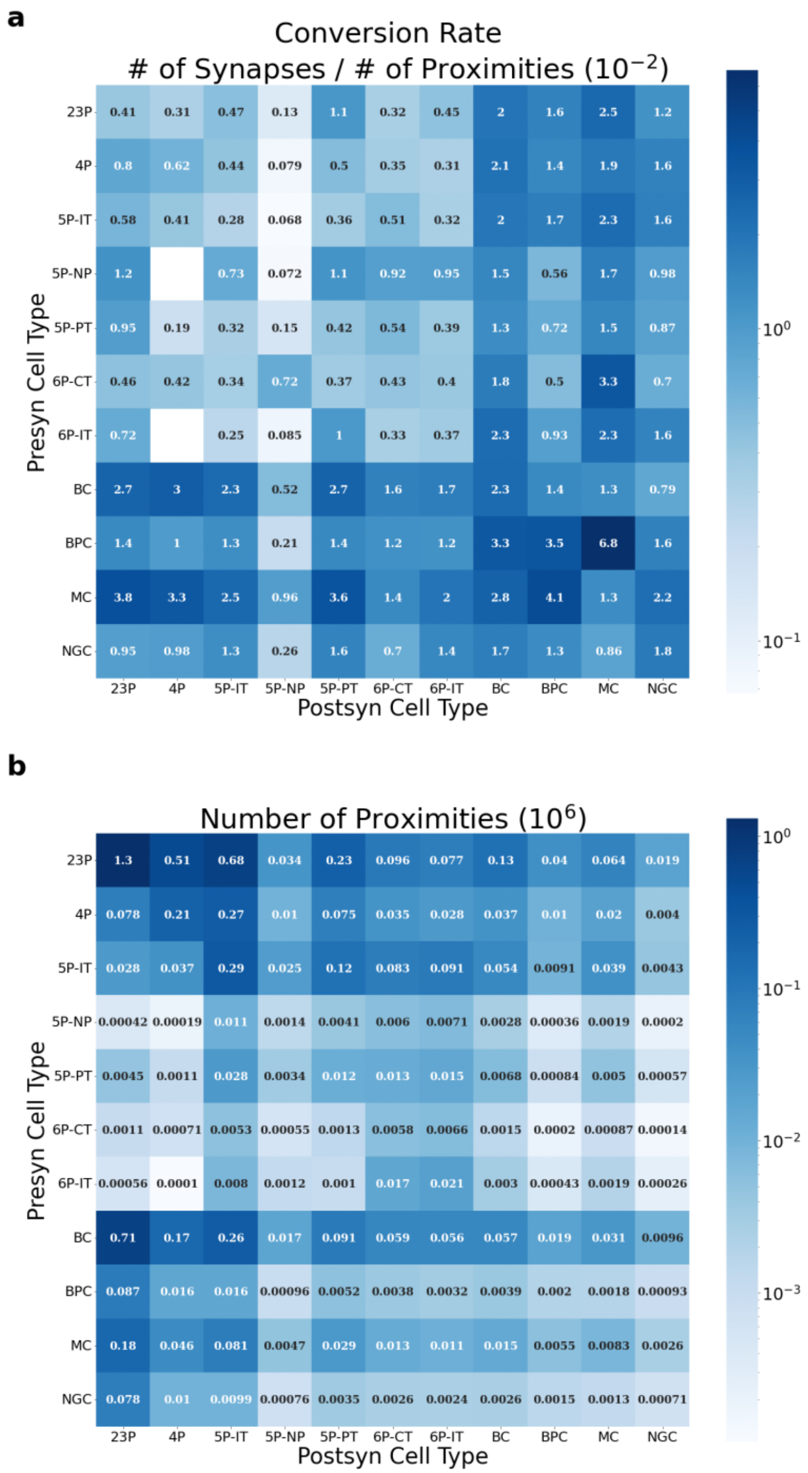

Supplemental Fig. 24. Conversion Rate Cell Type Matrix The conversion rates (number of synapses / number of proximities) for different presynaptic and postsynaptic cell type pairs. The cell type labels are determined by the GNN whole neuron classifier. Proximities are filtered to only include those with the following features: less than 3 μm proximity distance, dendrite only postsynaptic compartment, presynaptic proximity width less than 130 nm (to exclude myelinated axon), presynaptic and postsynaptic cell type labels with at least a 70% confidence for each from the GNN classifier. **a** Conversion rate for different cell type presynaptic and postsynaptic combinations **b** Number of proximities in dataset used to calculate the conversion rate.

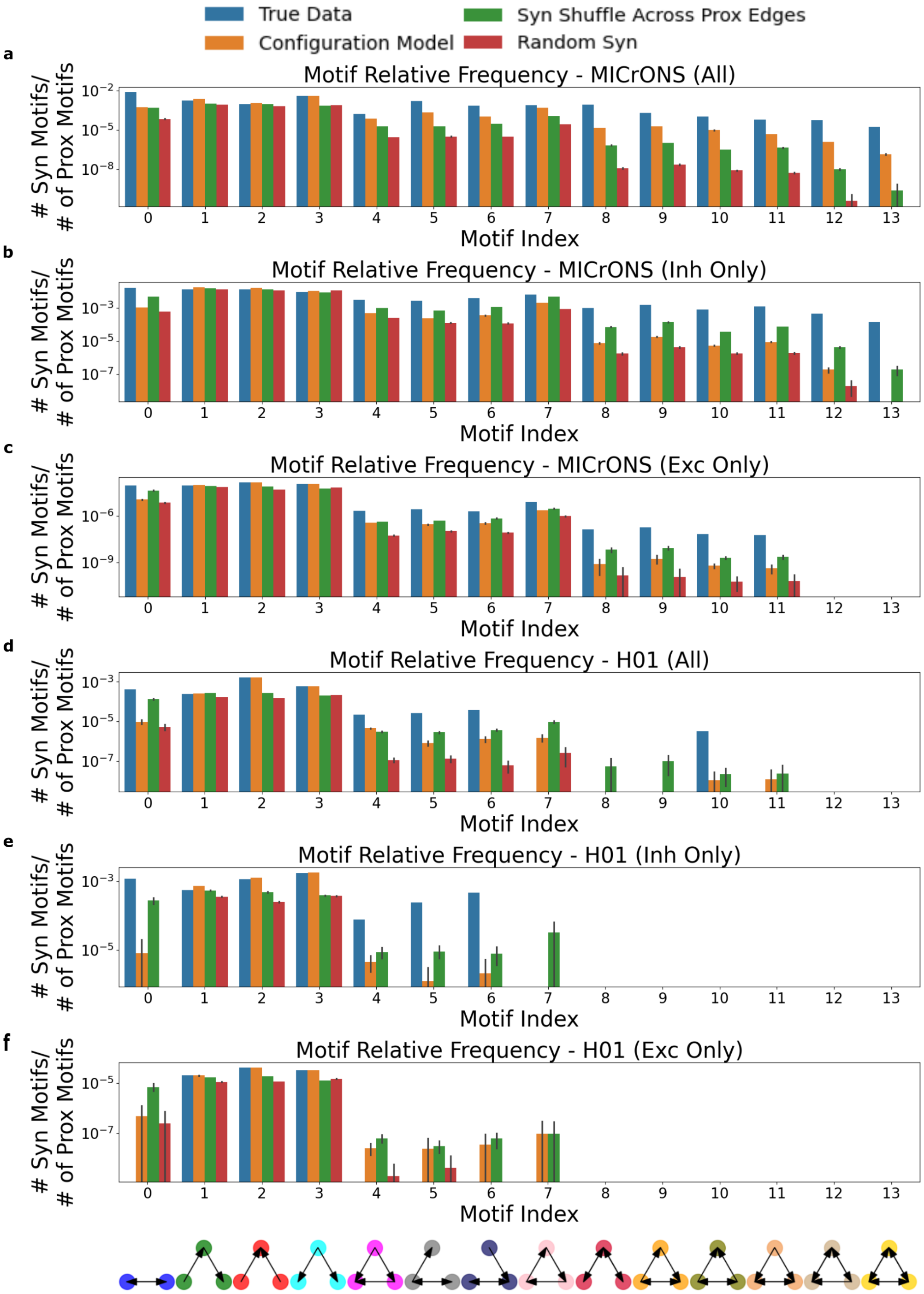

Supplemental Fig. 25. Higher-order Triangle Motif Analysis. Across all automatically-proofread neurons, we count the number of reciprocal connections and directed triangle motifs in the synaptic and proximity connectome and compare the observed ratios to null ratios from three different models: first, a model where synaptic degree distribution is held the same but edges are shuffled (configuration model), second, a model where the synaptic edges are shuffled only between neurons with an existing proximity edge, or third, a model where synapses are randomly shuffled between neurons regardless of proximity. In addition to testing on the entire connectome ("All"), we also performed the same set of comparisons on the inhibitory ("Inh Only") and excitatory ("Exc Only") subgraphs to test whether higher-order motif frequencies were consistent in these subpopulations. All plots display the mean +/- SD. **a-c** MICrONS dataset relative frequencies (duplicated from Fig. 5g) showing that the relative frequency of higher-order motifs in the synaptic connectome decreases as the number of edges in the motif increases (more higher-order), but are consistently higher than the null model controls (250 random graph samples for each null distribution comparison) for all subgraphs. **d-f** H01 dataset relative frequencies showing that the relative frequency of higher motifs in the synaptic connectome decreases as the number of edges in the motif increases. The observed motif frequencies are again higher than the null models (except in some more edge-dense 3 node motifs in the inhibitory and excitatory only subgraphs), but many of the motifs with more than three directed edges are not observed due to the more incomplete reconstruction of neurons in H01 (400 random graph samples for each null distribution comparison, more samples were computed than MICrONS because computation was faster with a smaller connectome).

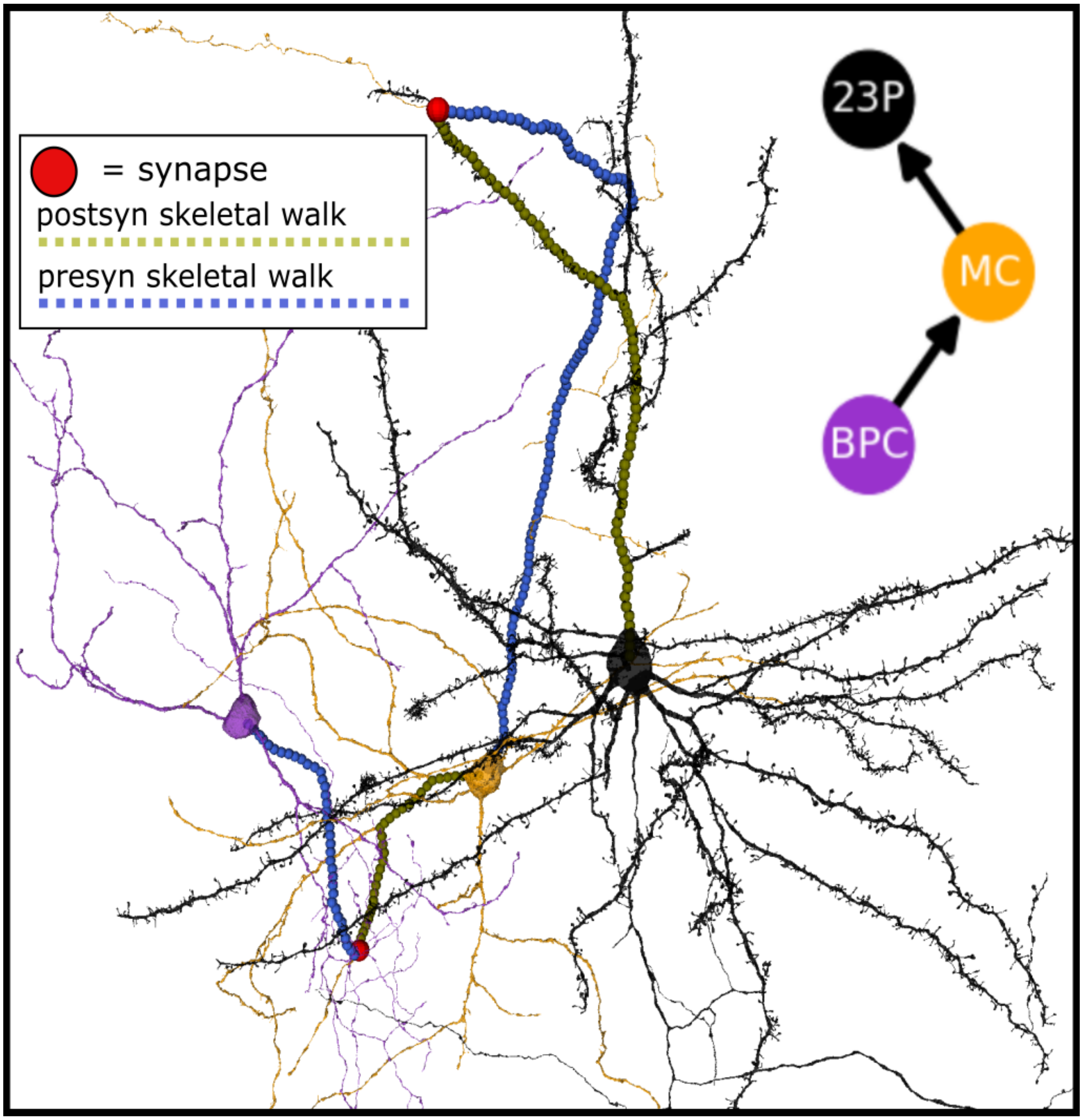

Supplemental Fig. 26. Higher-Order Cell Type Motif Search . Cell-type specific connections and motifs in the MICrONS dataset can be found by querying the annotated connectivity graph. NEURD allows for visualization of these connection paths and motifs so they can be quickly inspected.

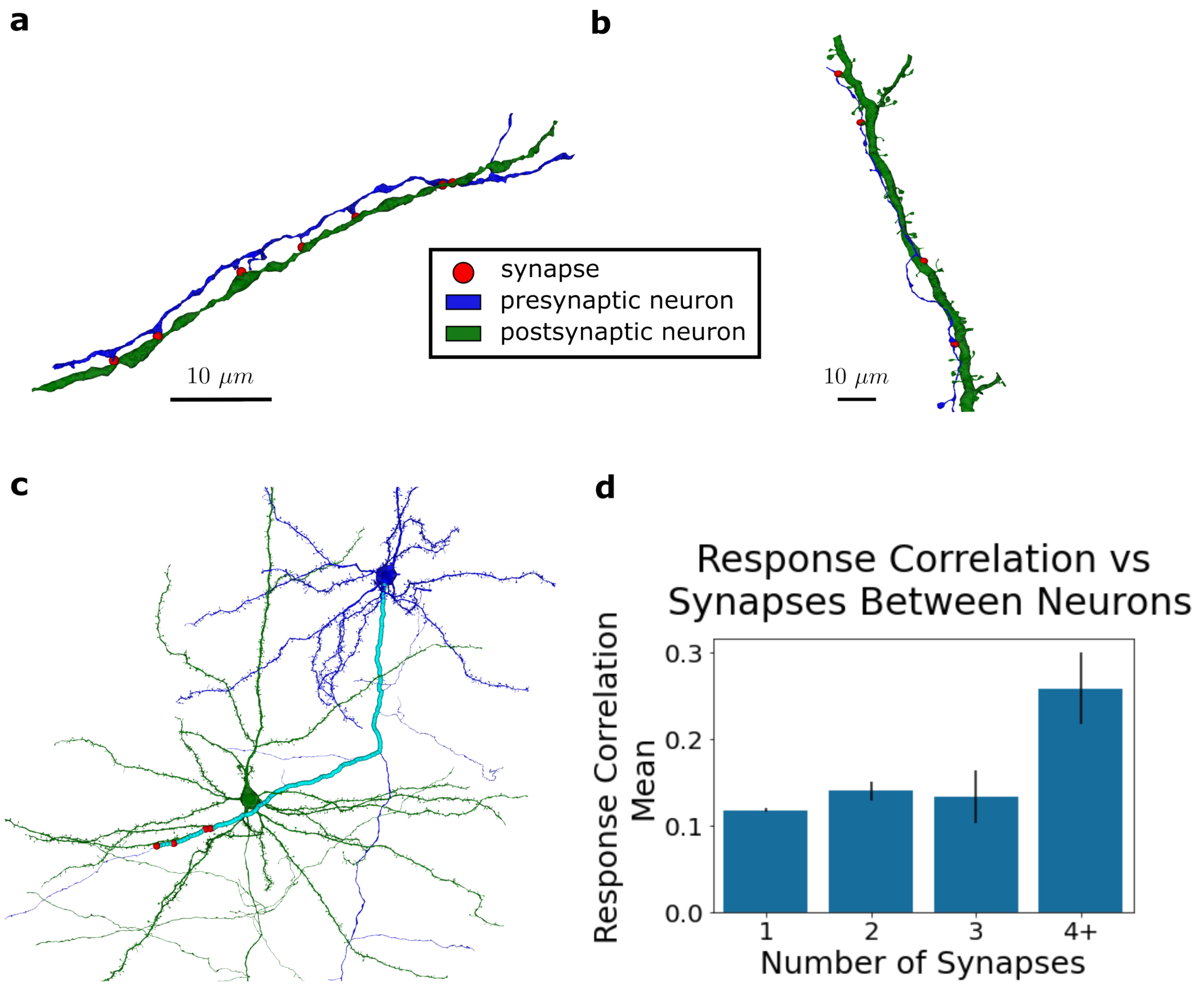

Supplemental Fig. 27. Functional Connectomics Illustration: High-degree cell pairs a Example multi-synaptic connection (n=7 synapses) from an excitatory to inhibitory neuron in the H01 dataset **b** Example multi-synaptic connection (n=4 synapses) from excitatory to excitatory neuron in the MICrONS dataset. **c** Example of a highly spatially clustered multisynaptic connection (n=4 synapses) on a postsynaptic basal dendrite from a neuron cleaned with automated proofreading in the MICrONS dataset (presynaptic skeletal walk shown in aqua, synapses shown in red) **d** Distribution of response correlation mean (mean +/- SEM) between pairs of functionally matched excitatory neurons in the MICrONS dataset. Response correlation is significantly larger for pairs of neurons with 4 or more synapses connecting them (n=11 pairs) compared to those with 1, 2, or 3 synapses (n=5350, 280, 34 pairs respectively).

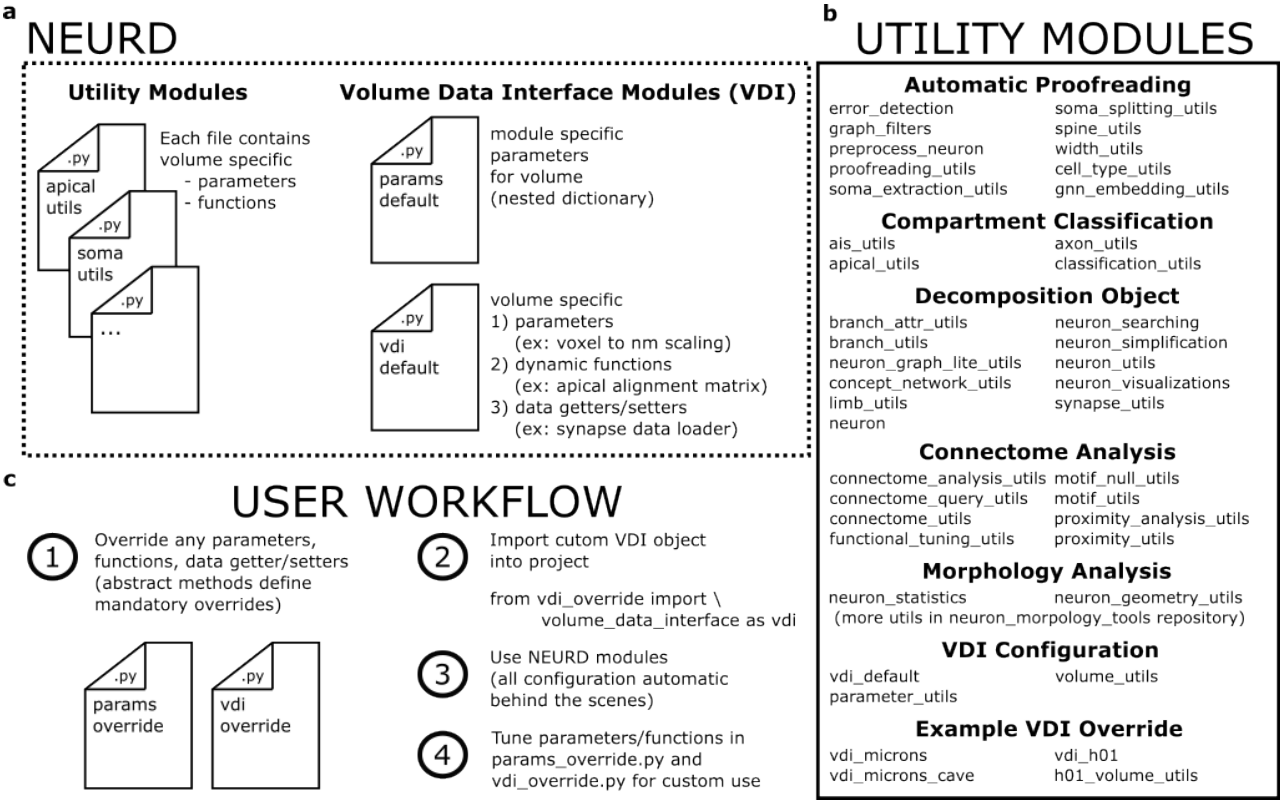

Supplemental Fig. 28. NEURD Code Package Overview. a The general structure of the code package modules. Most of the modules (.py files) are utility functions that define the functionality pertaining to a specific processing step (ex: *error_dection.py*) or neuron feature (ex: *spine_utils.py*). There are then two .py files that each define a "Volume Data Interface" (VDI). Together they set the user's settings/parameters for NEURD and facilitate data setting and fetching. The VDI modules have default implementations, but NEURD users can override these files for custom implementations (examples of how to override these VDI files are provided in the code documentation). **b** List of utility modules available in NEURD at the time of publication **c** Step by step procedure for how a user would override/configure, import and use the NEURD package

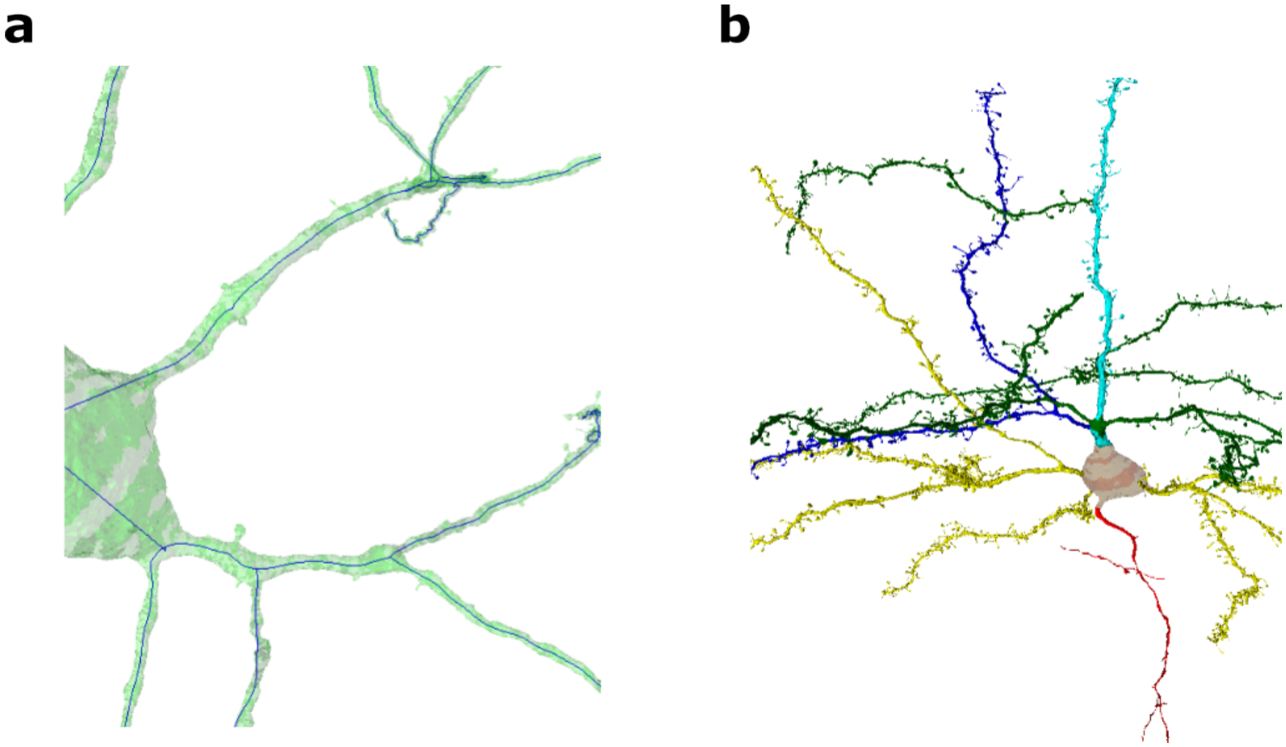

Supplemental Fig. 29. Skeleton and Compartment Visualization a A general example of a skeleton generated by the NEURD package where most thick dendritic segments are represented by a skeleton that tracks the inside middle of the segment, and thinner segments are represented with surface skeletons. The inhibitory neuron in this example is available for 3-D view and closer inspection using the NEURD tutorials on the GitHub repository. **b** A general example of compartment labeling of a neuron's mesh and skeleton. The excitatory neuron in this example is available for 3-D view and closer inspection using the NEURD tutorials on the NEURD GitHub repository.

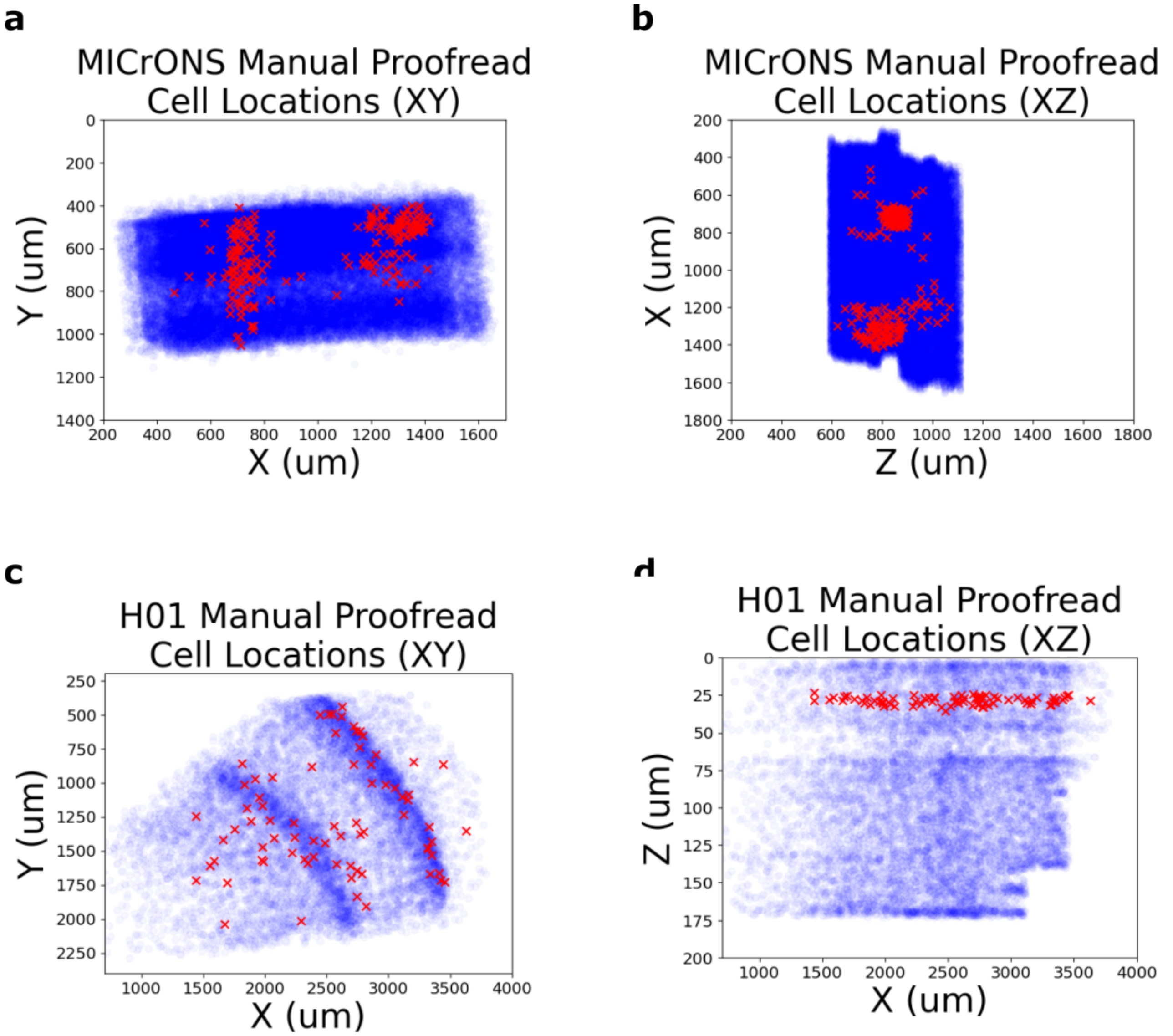

Supplemental Fig. 30. Manual Proofread Cell Locations a,b Locations in the volume (indicated by red "x" markers) of all manually proofread cells used in the MICrONS validation test set. All cells in the volume are plotted in the background in blue. **c,d** Locations in the volume (indicated by red "x" markers) of all manually proofread cells used in the H01 validation test set. All cells in the volume are plotted in the background in blue.

Supplemental Tables

Abbreviation	Cell Type	Excitatory/Inhibitory
23P	Layer 2/3 Pyramidal	Excitatory
4P	Layer 4 Pyramidal	Excitatory
5P-IT	Layer 5 Intratelencephalic	Excitatory
5P-NP	Layer 5 Near Projecting	Excitatory
5P-ET	Layer 5 Pyramidal Track	Excitatory
6P-CT	Layer 6 Corticothalamic	Excitatory
6P-IT	Layer 6 Intratelencephalic	Excitatory
BC	Basket Cell	Inhibitory
BPC	Bipolar Cell	Inhibitory
MC	Martinotti Cell	Inhibitory
NGC	Neurogliaform Cell	Inhibitory

Supplemental Table 1. Abbreviations and excitatory/inhibitory classifications for cell type subclasses in MICrONS dataset

Supplemental Table 2. See External Document "Supplemental Table 2 - Figure Data Size (N).xlsx"

Supplemental Table 3. See External Document "Supplemental Table 3 - Neuron Feature Documentation.xlsx"

Supplemental Discussion

Highly-annotated NEURD graphs provide a compact representation of many features that are useful for all kinds of morphological analysis. For example, a simple query reveals that the percentage of pyramidal cells with axons protruding from dendrites (rather than the soma) is higher in the mouse volume (17.8%) than the human volume (8%), which closely replicates the findings of an entire previous study focused on this question[27]. Several of the morphological properties of cell types shown in Fig. 4g-h and Supplemental Fig. 15 replicate observations from previous studies[21,20,28]. Additionally, using the spine metrics extracted by NEURD, we were able to replicate many of the findings of[25,26] concerning synapse size and spine head volumecorrelation (Pearson's), and we also show that these scaling rules and others depend on cell type (Fig. 4h, Supplemental Fig. 20). Looking at how synapses onto the AIS and soma vary across species, we find the expected lower rate of soma synapses on human neurons than mouse[23], and replicate the expected distribution of AIS synapses across depth in the mouse dataset[22]. We also find that the human dataset does not show a similar change over depth in AIS synapses, and that the human dataset AIS is more densely innervated than in the mouse volume (Supplemental Fig. 16g-h, Supplemental Fig. 16i). Finally, we demonstrate the use of a query combining geometric information and branch-level characteristics to replicate the previously-reported bias in the orientation of the thickest basal segment in the H01 dataset[5]. We extend this finding with an observation that this bias is actually consistent across all depths but is just less salient in upper layers because the relative size of the thickest and second-thickest basal dendrites changes smoothly across depth (Supplemental Fig. 17b,c).

Using the cell type node labels and the skeletal walk length edge features, we confirmed previous work[29,30] describing different distal and proximal preferences for different excitatory and inhibitory connection types (Fig. 5d-e, Supplemental Fig. 23f-g). Furthermore, using the proximity controls computed on the cleaned skeletons, we also were able to observe a consistent trend across datasets showing the propensity for forming connections from excitatory to inhibitory neurons peaks around 200 μm away from the soma, potentially consistent with a pattern of surround suppression (Supplemental Fig. 23d,e). Additionally, with the cell type node labels and spine compartment and synapse size edge labels, we confirmed a variety of expected findings about synaptic and spine head size: excitatory to excitatory connections have the largest synapses, synapse size correlates with spine heads for excitatory sources but not inhibitory sources, and spine heads with inhibitory synapses generally are multi-synaptic spines where the inhibitory synapse is typically much smaller than the largest synapse on the spine head (Supplemental Fig. 21).
