# Supplementary material for: NEURD offers automated proofreading and feature extraction for connectomics
Source: Nature. 2025 Apr 9;640(8058):487–96. doi: 10.1038/s41586-025-08660-5 (PMC11981913; doi:10.1038/s41586-025-08660-5)
Supplement: Supplementary file 2 — Reporting Summary [file 41586_2025_8660_MOESM2_ESM.pdf]

nature portfolio

Corresponding author(s): Jacob Reimer

Last updated by author(s): Apr 5, 2023

Reporting Summary

Nature Portfolio wishes to improve the reproducibility of the work that we publish. This form provides structure for consistency and transparency in reporting. For further information on Nature Portfolio policies, see our Editorial Policies and the Editorial Policy Checklist.

Statistics

For all statistical analyses, confirm that the following items are present in the figure legend, table legend, main text, or Methods section.

n/a	Confirmed	
☐	☒	The exact sample size (*n*) for each experimental group/condition, given as a discrete number and unit of measurement
☐	☒	A statement on whether measurements were taken from distinct samples or whether the same sample was measured repeatedly
☐	☒	The statistical test(s) used AND whether they are one- or two-sided<br>*Only common tests should be described solely by name; describe more complex techniques in the Methods section.*
☐	☒	A description of all covariates tested
☐	☒	A description of any assumptions or corrections, such as tests of normality and adjustment for multiple comparisons
☐	☒	A full description of the statistical parameters including central tendency (e.g. means) or other basic estimates (e.g. regression coefficient) AND variation (e.g. standard deviation) or associated estimates of uncertainty (e.g. confidence intervals)
☐	☒	For null hypothesis testing, the test statistic (e.g. *F*, *t*, *r*) with confidence intervals, effect sizes, degrees of freedom and *P* value noted<br>*Give P values as exact values whenever suitable.*
☒	☐	For Bayesian analysis, information on the choice of priors and Markov chain Monte Carlo settings
☒	☐	For hierarchical and complex designs, identification of the appropriate level for tests and full reporting of outcomes
☐	☒	Estimates of effect sizes (e.g. Cohen's *d*, Pearson's *r*), indicating how they were calculated

Our web collection on statistics for biologists contains articles on many of the points above.

Software and code

Policy information about availability of computer code

Data collection	For image acquisition, we used ScanImage 2017b. Stimuli were presented using PsychToolBox 3. The data collection process was automated with Labview.
Data analysis	Python 3.8 was the primary software and version used for this work. We used CAIMAN for automatic segmentation and deconvolution of calcium imaging data. For mesh processing we used CGAL (4.11.1000), meshlab (meshlabserver: 1.0.0), trimesh (4.4.1), and meshparty (1.16.14). For visualizations we used Neuroglancer (https://github.com/seung-lab/neuroglancer, tag v1.0.10 - v2.36) and ipyvolume (0.6.3). Our custom built analysis pipeline (https://github.com/reimerlab/NEURD.git, release 0.1.4) also used general tools like Numpy (1.24.4), Pandas (2.0.3), SciPy (1.10.1), scikit-learn (1.3.2), PyTorch (1.10.2+cpu), matplotlib (3.7.2), seaborn (0.13.0), Jupyter (ipykernel: 6.25.1), Docker (23.0.1), and Kubernetes (1.22.11). For basic graph representations and querying we used networkx (2.8.8), dotmotif (0.9.2b), and netSci (0.0.3). For storing and managing data we used DataJoint (0.12.9) and CAVE (4.12, 4.14, 4.16).

For manuscripts utilizing custom algorithms or software that are central to the research but not yet described in published literature, software must be made available to editors and reviewers. We strongly encourage code deposition in a community repository (e.g. GitHub). See the Nature Portfolio guidelines for submitting code & software for further information.

Data

Policy information about availability of data

All manuscripts must include a data availability statement. This statement should provide the following information, where applicable:

- Accession codes, unique identifiers, or web links for publicly available datasets
- A description of any restrictions on data availability
- For clinical datasets or third party data, please ensure that the statement adheres to our policy

All MICrONS data have already been released on BossDB (https://bossdb.org/project/microns-minnie, please also see https://www.microns-explorer.org/cortical-mm3 for details). All H01 data is as well public, with access instructions on their homepage (https://h01-release.storage.googleapis.com/data.html). Additionally, packages like NAVIS (https://navis-org.github.io/navis/) exist to facilitate easier access to both datasets. Therefore, because all datasets are publicly available and the NEURD package is publicly available with extensive tutorials on how to run each stage in the pipeline, all data products of interest (used in the study and figures) can be reproduced on demand and thus are not provided through another public API. However, for convenience some data products (such as synapse spine labels and cell type subclasses) are already publicly available through CAVE (https://caveconnectome.github.io/CAVEclient/).

Human research participants

Policy information about studies involving human research participants and Sex and Gender in Research.

Reporting on sex and gender	N/A
Population characteristics	N/A
Recruitment	N/A
Ethics oversight	N/A

Note that full information on the approval of the study protocol must also be provided in the manuscript.

Field-specific reporting

Please select the one below that is the best fit for your research. If you are not sure, read the appropriate sections before making your selection.

☒ Life sciences ☐ Behavioural & social sciences ☐ Ecological, evolutionary & environmental sciences

For a reference copy of the document with all sections, see nature.com/documents/nr-reporting-summary-flat.pdf

Life sciences study design

All studies must disclose on these points even when the disclosure is negative.

Sample size	No sample-size calculation was performed a priori. Sample sizes (number of connections tested) match or exceed previous studies of similar design. We are restricted to the singular EM volumes in the MICrONS and H01 datasets.
Data exclusions	Of the 14 released MICrONS scans, one scan was excluded a priori from the study due to experimental issues (responses to some stimuli were not collected due to water running out from the objective). For any morphological or connectivity analysis, neurons that errored out in our preprocessing pipeline (mostly due to corrupted, missing or difficult to process submeshes) were not present in downstream analyses. For the analysis involving functional data in the MICrONS dataset, neurons that did not pass the pre-established functional thresholds described in the paper were excluded from the analysis in order to only compare functional properties in neurons that were well characterized.
Replication	Due to the cost and time involved in producing the MICrONS and H01 volume, second volumes are not yet prepared to allow reproducibility testing.
Randomization	No randomization is performed since our study did not include multiple predefined experimental groups for sample allocation.
Blinding	No blinding is performed during data collection since our study did not include predefined experimental groups for sample allocation.

Reporting for specific materials, systems and methods

We require information from authors about some types of materials, experimental systems and methods used in many studies. Here, indicate whether each material, system or method listed is relevant to your study. If you are not sure if a list item applies to your research, read the appropriate section before selecting a response.

Materials & experimental systems

n/a	Involved in the study
☒	☐ Antibodies
☒	☐ Eukaryotic cell lines
☒	☐ Palaeontology and archaeology
☒	☐ Animals and other organisms
☒	☐ Clinical data
☒	☐ Dual use research of concern

Methods

n/a	Involved in the study
☒	☐ ChIP-seq
☒	☐ Flow cytometry
☒	☐ MRI-based neuroimaging
